# Lactate: elucidating its indispensable role in human health

**DOI:** 10.1186/s12943-025-02519-z

**Published:** 2026-01-05

**Authors:** Shengxin Zhang, Jie Wang, Ziyu Xu, Ziyang Cheng, Bin Shao, Jiayun Yu

**Affiliations:** 1 Department of Radiotherapy, Cancer Center, State Key Laboratory of Biotherapy, West China Hospital, National Clinical Research Center for Geriatrics, Sichuan University, Chengdu, 610041 China; 2 Department of Oncology, The First Affiliated Hospital of Zhengzhou University, Zhengzhou, Henan China; 3 Suzhou Medical College, Soochow University, Suzhou, China; 4 Department of Biotherapy, State Key Laboratory of Biotherapy and Cancer Center, West China Hospital, Sichuan University, Chengdu, Sichuan PR China; 5 Department of Cardiovascular Surgery and Cardiovascular Surgery Research Laboratory, West China Hospital, Sichuan University, Chengdu, Sichuan PR China

**Keywords:** Lactate metabolism, Lysine lactylation, Epigenetic regulation, Immune modulation

## Abstract

Lactate, traditionally considered just a byproduct of metabolism, is now understood to be a vital regulator in energy metabolism, immune function, and epigenetic changes. Besides serving as an alternative energy source through the “lactate shuttle,” it acts as a signaling molecule influencing both normal and abnormal processes in various organs. New research has emphasized its role in lactylation of histones and non-histones, a novel post-translational modification linking metabolic activity with gene expression and immune response. Lactate contributes to immunosuppression, angiogenesis, and the spread of tumors within the tumor microenvironment. Its accumulation is also linked to cardiovascular, metabolic, and neurodegenerative conditions. This shift in metabolism underscores lactate’s growing importance in both health and disease, presenting novel therapeutic opportunities, especially in the treatment of cancer and metabolic disorders. This review synthesizes emerging insights into lactate’s multifaceted roles and discusses promising therapeutic strategies targeting lactate metabolism, transport, and downstream signaling pathways, with an emphasis on candidates advancing toward clinical translation.

## Introduction

Lactate has long been viewed as a metabolic waste product, and its production was historically believed to be associated primarily with strenuous exercise or hypoxic conditions [1, 2]. However, this perspective has evolved significantly, with accumulating evidence showing that lactate is continuously produced and utilized even under well-oxygenated conditions in organs like the brain and heart [3].

Structurally, lactic acid consists of three isomers: D-lactic acid, L-lactic acid, and DL-lactic acid, with L-lactic acid being the dominant form in mammals [4]. Lactate is primarily produced via glycolytic metabolism. While this process is often associated with low-oxygen environments, it also occurs under aerobic conditions in metabolically active tissues such as the brain, heart, and resting skeletal muscle, where lactate dehydrogenase (LDH) facilitates the reversible conversion of pyruvate into lactate in the cytoplasm [3, 4]. In addition, glutamine metabolism may also contribute to lactate production in cancer cells. With a better understanding of the “Warburg effect”, it has been found that highly proliferative cells still prefer glycolysis for lactate production even in an aerobic environment [5]. This phenomenon is not restricted to tumor cells but is also manifests itself in a variety of non-neoplastic pathological conditions, such as pulmonary vascular remodeling, cardiac hypertrophy, and multiple sclerosis, and its metabolic inhibitors have shown potential for therapeutic use. This phenomenon is not limited to tumor cells, but also manifests itself in a variety of non-tumor pathological conditions, such as pulmonary vascular remodeling, cardiac hypertrophy, and multiple sclerosis, and its metabolic inhibitors have shown some therapeutic potential [6, 7].

Brooks proposed the theory of the “lactate shuttle”, indicating that lactate can also act as an energy source via oxidative pathways in different organs and cells in an aerobic environment, facilitating metabolic coordination across tissues [8, 9]. Lactate plays a crucial role in maintaining redox homeostasis by regulating the NAD⁺/NADH ratio. After its intracellular production, lactate can enter the circulation via monocarboxylic acid transporters (MCTs) and be further taken up by distal tissues for energy metabolism [10, 11]. Another key mechanism involves lactate acting as a signaling mediator via the G protein-coupled receptor GPR81 (also known as HCAR1). Moreover, the presence of lactate transporters and pannexin-based gap junctions in astrocytes highlights their unique role in central nervous system function [12–14].

While the foundational role of lactate in cellular metabolism has been well-established for years, recent discoveries have significantly expanded our understanding. In 2019, lactylation was identified as a novel post-translational modification (PTM) on both histone and non-histone proteins, establishing a direct link between lactate and gene expression regulation. This lactylation process, catalyzed by ‘writer’ proteins like acetyltransferase p300, and reversible by ‘eraser’ proteins such as histone deacetylases (HDACs), reveals lactate’s role as an epigenetic regulator and intercellular communicator [15].

Furthermore, lactate has emerged as a key player in immune regulation, with evidence pointing to its impact on immune cell function through mechanisms such as receptor signaling via GPR81 and histone lactylation, which are closely linked to its immune-modulatory effects [16]. Additionally, lactate metabolism exert its impact on the development of many diseases, including autoimmune diseases, cardiovascular diseases, and metabolic syndrome [17–19]. It is also increasingly recognized for its role in the microbiome, where it influences gut health and microbial balance, further extending its biological functions [20]. In the tumor microenvironment (TME), lactate even contributes to tumor progression through mechanisms such as immunosuppression, angiogenesis, metastasis promotion and epigenetic reprogramming [21, 22].

This review highlights recent advances in lactate biology, focusing on its multifaceted roles in energy metabolism, immune regulation, gene expression, and disease development across multiple organ systems. Lactate is now recognized not only as a metabolic intermediate but also as a signaling mediator and regulatory molecule that orchestrates diverse physiological and pathological processes through metabolic, immune, and epigenetic mechanisms. In light of these insights, this review further explores emerging therapeutic strategies targeting lactate metabolism, transport, and downstream immune or epigenetic pathways. Throughout this review, we will carefully delineate mechanistic insights derived from experimental models from evidence obtained in clinical settings, thereby providing a clear perspective on the translational path of lactate biology.

## Lactate: from byproduct to central metabolite

### Lactate production and metabolic origins

Lactate is widely recognized as a key regulator in cellular energy metabolism. Under physiological conditions, its concentration is typically maintained within a narrow range of 0.5 to 2.2 mmol/L. It is distributed and released from all parts of the body (muscle, skin, brain, and intestines). The clearance of lactate is mainly dependent on liver metabolism through Cori cycle while myocytes and kidney cells can also utilize it as the source of energy under specific conditions [23, 24].

Generally speaking, lactate production is typically triggered under hypoxic conditions, when cells shift from aerobic respiration to glycolysis using the same substrate pyruvate. Under aerobic conditions, pyruvate is converted to ATP via mitochondrial oxidation, whereas under hypoxia, it is transformed into lactate by LDH, which also plays a crucial role in gluconeogenesis and lactate biosynthesis [15, 25]. While glycolysis from glucose is the predominant source of lactate under most physiological conditions, certain metabolic contexts utilize alternative precursors. Notably, in some cancer cells under hypoxic conditions or with mitochondrial dysfunction, glutamine metabolism can contribute to lactate production via anaplerotic pathways such as reductive carboxylation [26, 27]. In this setting, glutamine-derived α-ketoglutaric acid (α-KG) is converted to isocitrate and then to citrate through reductive carboxylation, followed by the generation of pyruvate and lactate, providing a pathway for lactate synthesis independent of glucose metabolism [4, 27].

The quantitative impact of glutamine on the overall lactate pool varies significantly depending on the context, and it is typically smaller in comparison to glucose [28]. This is underscored by in vivo isotopic tracing studies in human lung cancers, which, despite revealing that circulating lactate can serve as a major respiratory fuel surpassing glucose in some tumors, simultaneously demonstrated that glucose, instead of glutamine, remains the primary substrate for carboxylation anaplerosis within tricarboxylic acid (TCA) cycle [29]. This highlights that while the glutamine-to-lactate pathway is operational, its overall significance in fuel economy is often secondary to glycolysis-derived carbon. The central role of lactate in systemic energy metabolism is further exemplified by the Cori cycle. Studies have shown that during intense physical activity, a significant portion of glucose production originates from lactate consumed via this cycle. This process not only demonstrates lactate’s function as a key gluconeogenic precursor but also solidifies its position as a central energy carrier, efficiently shuttling carbon between glycolytic and oxidative tissues [30].

Although lactate has been widely accepted as a key fuel source in mitochondrial metabolism, whether it can be directly oxidized in mitochondria is still controversial. Some studies have proposed the concept of mitochondrial lactate oxidation complex (mLOC), comprising mitochondrial LDH (mLDH), MCT1, and CD147, which may mediate the direct entry of lactate into mitochondria for subsequent utilization. Supporters of this model have observed co-localization of these proteins in mitochondrial membranes of oxidative tissues [8, 31]. However, other studies have challenged the presence or functional relevance of mLOC, arguing instead that lactate is likely to first be converted to pyruvate in the cytosol before being imported into mitochondria via the mitochondrial pyruvate carrier (MPC) [32]. Thus, while the lactate-TCA cycle-oxidative phosphorylation (OXPHOS) axis is well supported, whether lactate enters mitochondria directly or is first converted to pyruvate in the cytosol remains uncertain, and the preferred route may vary across different tissue types or metabolic conditions [2].

### Lactate as an alternative fuel

L-lactate functions as a key mediator acting as mitochondrial fuel, undergoing conversion to pyruvate and feeding into downstream oxidative metabolic pathways to sustain cellular energy output. This ‘lactate-TCA-OXPHOS’ metabolic axis reveals that lactate is not only a by-product of glycolysis, but also an important mitochondrial fuel for maintaining energy homeostasis in cells, including tumor cells, under fluctuating oxygen conditions [8, 28]. Besides, in skeletal muscles, lactate accumulation alters the NADH/NAD^+^ ratio in parallel with changes in the ADP/ATP ratio, thereby promoting ongoing oxidative phosphorylation. Similar metabolic responses are observed in brain tissues, suggesting lactate’s extensive role in metabolic regulation across multiple organs and its importance in maintaining redox balance [33].

Additionally, lactate can serve as an important substrate in metabolic activities and provides a supplemental energy source for oxidative metabolism. The NAD^+^/NADH ratio, as a dominant parameter, reflects fluctuations in intracellular lactate abundance, aligning with regulation by LDH and mitochondrial complexes [34]. LDH modulates lactate-pyruvate interconversion, which is accompanied by the electron shuttle, while mitochondrial complexes maintain oxidative respiration. Interestingly, the activation of the pyruvate dehydrogenase (PDH) complex results in a decrease in the NAD^+^/NADH ratio, indicating an increase in NADH production. When the demand for NAD^+^ in oxidative processes exceeds the rate of NAD^+^ regeneration in mitochondria, metabolic flux shifts toward glycolysis. This dynamic adjustment may subsequently modulate PDH activity [10].

### Lactate transportation

#### Mechanisms of lactate transmembrane

The most representative transporters responsible for both intercellular and intracellular lactate shuttle are MCTs [35]. Monocarboxylate transporters are capable of recognizing a wide range of substrates, including lactate, pyruvate, acetoacetate, and various ketone compounds. Among the identified isoforms, MCT1 through MCT4 have been most extensively examined in terms of their structural features and physiological roles [35, 36]. CD147 functions as an ancillary protein, facilitating the colocalization of MCT1/4 and promoting the membrane trafficking of MCT1 and MCT2 [37]. In preclinical tumor models, MCTs have been demonstrated to play key roles in tumor metabolism by regulating lactate exchange [38].

In addition to their transport functions, MCTs also participate in lactate-mediated signaling. For instance, lactate metabolism has been studied as a modulatory agent to induce specific signaling transduction, and lactate enters the cells through MCTs and engages with GPR81 receptors in an autocrine manner, thereby reducing cAMP generation and modulating the insulin-induced PI3K-AKT pathway [39–41]. Thus, lactate is increasingly recognized as a critical metabolic substrate that intersects with diverse cellular signaling networks [42].

#### Mechanisms of lactate transport

The mechanisms of lactate transport are primarily regulated by concentration gradients, proton coupling, and transporter expression patterns [8, 43]. Oxidative cells such as those in well-oxygenated tumor regions typically express MCT1, a high-affinity monocarboxylate transporter that promotes lactate uptake for mitochondrial oxidative phosphorylation [35]. Since MCTs function via passive diffusion, lactate transport depends on the existing concentration gradients of both lactate and hydrogen ions across the membrane. Therefore, MCT1 can mediate lactate uptake in oxidative cells where lactate is consumed, but under specific conditions where the intracellular lactate concentration is high, it can also mediate lactate efflux to maintain metabolic homeostasis [35, 44, 45].

Additionally, MCT2 is chiefly responsible for transporting lactate into metabolically active tissues with rich blood supply [46]. For instance, in the central nervous system, astrocyte-secreted lactate flows into neurons through MCT2 as a fuel source, thereby promoting neuronal plasticity [47]. Beyond its role in energy supply, MCT2 (encoded by SLC16A7) has recently drawn attention for its involvement in neurological and neurodegenerative diseases. In the central nervous system, MCT2 is recognized as a high-affinity lactate transporter located on neurons and serves as a key component of the astrocyte-neuron lactate shuttle (ANLS) pathway. Within this pathway, astrocytes export lactate to the extracellular space via MCT1 and MCT4, after which neurons take up lactate through MCT2 and convert it to pyruvate for entry into the TCA cycle to support oxidative metabolism [48, 49]. A growing body of evidence also suggests that dysregulation of the MCT2-mediated lactate shuttle contributes to neuronal metabolic stress and synaptic dysfunction, particularly in neurodegenerative conditions [49–51]. For instance, dysregulation of MCT2-mediated lactate shuttle has been observed in neurological diseases, such as Alzheimer’s disease, suggesting its potential role in neuronal survival and disease progression [52]. Nevertheless, whether similar MCT2-related dysregulation occurs in other neurological disorders remains to be fully established and requires further validation. Moreover, MCT4 is significantly upregulated in cells with dominant glycolytic activity. As a proton-coupled transporter, it facilitates the co-efflux of lactate and protons, thereby eliminating these glycolytic byproducts and helping to maintain intracellular pH homeostasis [53]. This reflects a key implication in malignancies, whose intrinsic environment is highly dependent on glycolysis, as characterized by the *Warburg* effect. The amplification of MCT1 and MCT4 has been demonstrated in solid tumors and represents a dominant force controlling glycolytic activities [54].

In addition, CD147, also known as basigin or EMMPRIN, is essential for ensuring the proper folding, trafficking, and functionality of MCT1 and MCT4 transporters [55]. Studies have shown that CD147 forms stable, non-covalent complexes with MCT1 and MCT4, serving as an essential chaperone for their proper folding and trafficking to the plasma membrane [36, 56]. CD147 inhibitors lead to intracellular retention of MCT1/4, disrupting lactate transport and intercellular lactate exchange [55].

#### The “lactate shuttle” theory and tumor metabolic symbiosis

It should be noted that lactate accumulation per se does not directly cause acidification; rather, the proton-coupled transport through MCTs is the main driver of pH alterations observed in the TME [57, 58]. The “lactate shuttle” concept is now recognized as a fundamental mechanism for metabolic coordination and signaling under aerobic conditions. This shuttle operates through several coordinated models. The first is metabolic symbiosis between tumor cells: hypoxic cells export lactate and protons via MCT4, while normoxic neighbors import it via MCT1 for oxidative phosphorylation, thereby optimizing intra-tumoral resource utilization [45]. Further extending this paradigm, the “reverse Warburg effect” describes a stromal-epithelial coupling where cancer-associated fibroblasts (CAFs), under oxidative stress, activate hypoxia-inducible factor 1-alpha (HIF-1α) and release lactate via MCT4, which cancer cells then import via MCT1 to fuel their oxidative metabolism, creating an acidic, lactate-rich niche that supports tumor growth [59].

Beyond its metabolic role, lactate also acts as a signaling molecule by entering vascular endothelial cells via MCT1, where it stabilizes HIF-1α and activates pro-angiogenic pathways such as NF-κB/IL-8, thereby promoting tumor angiogenesis [60–62]. The critical role of this multi-faceted lactate shuttle is underscored by its validation across multiple tumor models (e.g., breast, prostate, osteosarcoma), where elevated MCT4 expression in CAFs is closely associated with enhanced tumor aggressiveness. Based on robust preclinical data across multiple cancer types, targeting MCT1 and MCT4 emerges as a mechanistically rational therapeutic strategy. This is supported by the ongoing clinical development of inhibitors like AZD3965 (MCT1, Phase I), which will ultimately test this hypothesis in human patients [63–69].

## Lactate-induced epigenetic modifications

### Histone and non-histone lactylation

Lactate production is markedly enhanced when cellular oxygen demand substantially exceeds supply. While the majority of lactate is derived from pyruvate, a fraction may also originate from glutamine decomposition. In addition to the classical L-lactate generated via LDH, alternative pathways contribute to the intracellular lactate pool. For instance, tumor cells are suggested to potentially produce D-lactate through the glyoxalase system [70, 71]. This pathway involves the metabolism of methylglyoxal (MGO) into lactoylglutathione (LGSH) by glyoxalase I (GLO1) with glutathione (GSH), followed by hydrolysis via glyoxalase II (GLO2) to yield D-lactate. It is important to note, however, that the physiological relevance and quantitative contribution of this glyoxalase/MGO-derived pathway to overall lactate metabolism and related lactylation modifications remain to be fully established. Accumulated lactate contributes to cytoplasmic acidosis, prompting cells to activate compensatory mechanisms for exporting excess lactate into the extracellular space and circulation. These dynamic regulatory processes underscore the precision of lactate metabolic control and reflect the complex homeostatic mechanisms governing its metabolism [72].

**Fig. 1:**
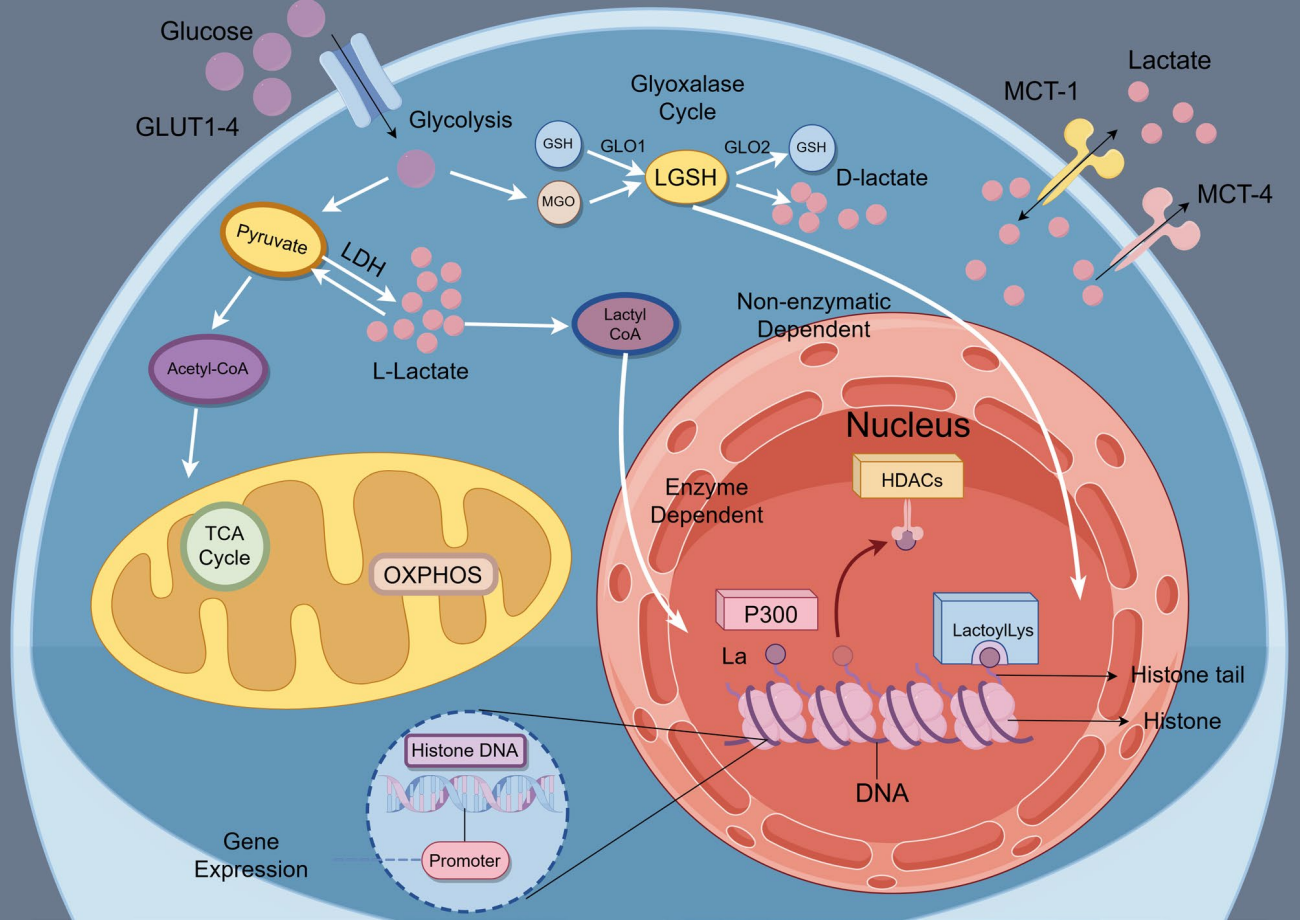
This schematic illustrates the process of lactate production and its involvement in lysine lactylation. During glycolysis, glucose is converted into lactate, which then enters cells through monocarboxylate transporters MCT1 and MCT4. Inside the cell, lactate serves as a precursor for lysine lactylation, either through enzymatic conversion to lactyl-CoA or via non-enzymatic pathways. The acetyltransferase p300 acts as the writer enzyme, while HDACs function as erasers. Specific reader proteins interpret these lactylation marks, ultimately influencing gene transcription and immune signaling processes. *Abbreviations*: *LDH* Lactate dehydrogenase, *OXPHOS* Oxidative phosphorylation, *TCA* Tricarboxylic acid cycle, *MCT* Monocarboxylate transporter, *Kla* Lysine lactylation, *HDACs* Histone deacetylases, *P300* E1A binding protein p300 (a histone acetyltransferase that acts as lactylation writer), *La* Lactate, Reader, protein domains that recognize modified histones and mediate downstream effects, *GLUT* glucose transporter, *GSH* Glutathione, *GLO1* Glyoxalase, *I* *GLO2* Glyoxalase II, *LGSH* Lactoylglutathione, *MGO* Methylglyoxal

Lactylation is a coping mechanism in response to lactate accumulation in the matrix. The most ubiquitously exhibited lactylation site is lysine. lysine lactylation (Kla) was elucidated to undergo transfer of lactyl groups from lactyl-CoA. L-lactate has been proposed to form lactyl-CoA, which may serve as a potential donor for enzymatic transfer by p300/CBP to lysine residues of histones, thereby generating Kla. Moreover, under conditions of GLO2 deficiency or LGSH accumulation, non-enzymatic transfer of lactoyl groups to lysine residues may occur, forming a putative modification termed LactoylLys. However, the presence of lactyl-CoA at physiological concentrations has not yet been directly demonstrated, and this pathway remains under debate [73]. It may represent a novel enzymatic signaling pathway; however, this prospect must be rigorously distinguished from the possibility that the signals are merely secondary modifications related to oxidative stress. The definitive establishment of the former necessitates direct validation of key intermediates such as lactyl-CoA [74]. This distinction is critical, as the field encompasses both specific enzymatic modifications and non-enzymatic chemical alterations that can occur under certain metabolic deficiencies or, more problematically, as technical artifacts during experimental procedures. Therefore, future research must prioritize the development and implementation of stringent methodological controls to delineate the precise contributions of enzymatic versus non-enzymatic mechanisms. Unraveling this interplay is paramount for understanding the true physiological and pathophysiological significance of protein lactylation. As summarized in Fig. 1, these distinct lactylation pathways highlight how metabolic intermediates feed directly into post-translational modifications.

Recent studies have highlighted Kla on both histone and non-histone proteins. Histone Kla is metabolically linked to glycolysis-derived lactate and functions predominantly in regulating gene expression through chromatin remodeling [75]. A seminal study by Zhang et al. further demonstrates that histone lactylation is a novel epigenetic mark that stimulates gene transcriptional activity on chromatin in response to elevated intracellular lactate levels, and is particularly prominent during M1-type macrophage polarization. It was shown that histone lactylation differentiates itself from conventional acetylation modifications in terms of temporal dynamics and induces the expression of genes involved in tissue repair, such as Arg1, during the later stages of macrophage activation [15].

In cancer, aberrant histone lactylation is frequently elevated and has been associated with poor prognosis, while functional inhibition of histone Kla has been reported to suppress tumor progression in vitro and in vivo. Mechanistically, recent studies have suggested that histone lactylation may upregulate the expression of the m6A recognition protein YTHDF2, thereby potentially influencing the degradation of oncogene transcripts such as PER1 and TP53 and contributing to tumorigenic processes. These observations provide preliminary evidence for the interplay between histone modifications and RNA epigenetics in cancer progression, although further studies are required to confirm these mechanisms [76].

Given that Kla is distinct with temporal dynamics from histone acetylation, it portraits a blueprint where lactate-modulated histone Kla can be perceived as a response to a surging cellular-lactate concentration owing to external stimulants [15]. Beyond histones, Kla has also been identified on a wide spectrum of non-histone proteins, intersecting with diverse cellular processes and suggesting broader roles in metabolic signaling and protein function [77]. Large-scale lactylation histology studies confirm the presence of Kla in a large number of non-histone proteins, including NCL, YY1, and HMGB1, and these modifications regulate transcription, signal transduction, and ribosome assembly by altering protein conformation, charge distribution, and molecular interactions, thereby revealing the broad regulatory potential of lactate as a signaling molecule [78–80]. Overall, while lactylation represents an intriguing link between metabolism and epigenetic regulation, the current understanding remains preliminary. Key questions including the existence of lactyl-CoA in vivo, the physiological relevance of non-enzymatic lactylation, and the identification of specific reader proteins must be resolved before lactylation can be fully established as a stable and universal regulatory mechanism.

### Regulatory enzymes of lactylation

During the modulation of lactylation dynamics, the coordinated actions of putative ‘writer’ and ‘eraser’ enzymes are proposed to contribute to the maintenance of epigenetic homeostasis and metabolic balance within the cell. The ‘writer’ enzyme is hypothesized to catalyze the transfer of lactate-derived metabolites (likely via lactoyl-CoA) to lysine residues on proteins, resulting in lactylation modifications that influence gene expression, chromatin structure, and cellular phenotype. In contrast, ‘eraser’ enzymes may remove these modifications, thereby modulating epigenetic plasticity and responding to metabolic cues. Although the identity of a bona fide lactyltransferase remains undetermined, the acetyltransferase p300 has been suggested as a candidate enzyme capable of mediating both histone (such as H3 and H4) and non-histone lactylation under certain conditions. For instance, in the fibrotic microenvironment where lactate accumulates and is taken up by macrophages and myofibroblasts, p300 has been implicated in promoting lactylation at macrophage-related loci, potentially contributing to the progression of pulmonary fibrosis [81]. Similarly, pharmacological inhibition of p300 has been reported to attenuate lactate-induced myoblast differentiation and reduce H3K9 lactylation at the Neu2 promoter, supporting a regulatory role of p300 in lactate-responsive transcriptional control [82].

Regarding ‘eraser’ enzymes, HDAC1, HDAC3, and members of the SIRT family have been identified in vitro as possessing delactylation activity [83]. Moreno-Yruela C et al. introduced the delactylases effectiveness of nicotinamide adenine dinucleotide-dependent HDACs. Researchers demonstrated that nuclear HDAC1-3, in association with SIRT (dual classified into class III HDACs), exhibited vibrant activities towards delactylases in vitro and inhibited Kla of both H3K18 and H4K5, among which HDAC3 predominates as the leading eraser in a zinc-dependent manner [83]. Nevertheless, these findings are largely based on in-vitro studies, and their in-vivo significance remains to be confirmed. To date, specific ‘reader’ proteins capable of recognizing lactyl-lysine residues have not yet been identified, and the molecular mechanisms underlying lactylation signal interpretation remain elusive. Collectively, while lactylation and delactylation appear to constitute a regulatory system analogous to other lysine acylations, our current understanding especially in living systems, remains preliminary and requires further experimental validation (Fig. 1).

### Functional implications of lactylation

#### Functional roles of histone lactylation

Histone lactylation represents one of the earliest identified forms of lactate-dependent epigenetic regulation. Zhang et al. initially identified histone lactylation in 2019, proposing it as a novel form of epigenetic regulation that preliminarily connected lactate metabolism to transcriptional control [15]. This modification depends on lactate availability and acts as a metabolic-chromatin bridge that modulates transcriptional output and cellular fate [15]. Functionally, histone lactylation influences gene transcription primarily through changes in chromatin accessibility rather than DNA sequence [75]. Compared with acetylation, Kla presents distinct temporal dynamics and is highly responsive to metabolic stress [15]. However, its in-vivo prevalence, regulatory enzymes, and biological significance remain to be comprehensively established.

Among the identified sites, H3K18la is the best characterized, promoting the transcription of tissue-repair and anti-inflammatory genes such as Arg1 during M1-to-M2 macrophage polarization [15, 84]. Notably, elevated H3K18la has also been linked to infection resolution, where it mediates a shift from pro-inflammatory to reparative gene programs [84]. Beyond immune regulation, lactylation also participates in developmental processes. Yang et al. (2021) reported that lactate-induced histone lactylation (H3K23la) contributes to uterine endometrial remodeling and supports early embryonic development, underscoring its regulatory role in reproductive physiology [85]. Subsequent studies have extended the functional spectrum of histone lactylation beyond immune regulation to developmental and differentiation contexts, which has been observed in embryonic stem-cell differentiation, endometrial reconstruction, and osteogenic development [86–88]. More recently, Li et al. (2025) revealed that lactate dynamically regulates histone lactylation at H4K5la, H4K8la, and H4K12la during meiotic prophase I in mouse spermatogenesis. H4K8la is enriched at promoters of meiotic genes and recombination hotspots, co-localizing with SPO11, DMC1, RAD51, and cohesin components (RAD21L, REC8), suggesting that lactylation serves as a metabolic cue coordinating gene activation and recombination during germ-cell meiosis [89].

Recent evidence further indicates that H3K14la contributes to neuronal ferroptosis after intracerebral hemorrhage by upregulating PMCA2 and impairing calcium efflux, thereby aggravating neural injury [90]. In head and neck squamous cell carcinoma (HNSCC), increased histone lactylation, particularly at H3K9la, is associated with poor immunotherapy response. H3K9la enhances the expression of interleukin-11 (IL-11), which subsequently triggers immune checkpoint activation through the JAK2/STAT3 signaling pathway, ultimately impairing CD8⁺ T cell activity and facilitating tumor growth. Knockdown of IL-11 reverses T cell exhaustion and enhances immunotherapy efficacy. Clinically, H3K9la correlates with IL-11 expression and unfavorable outcomes [91]. In addition, Cai et al. (2025) identified H3K27la as a lactate-responsive histone mark that promotes trained immunity. Through fueling the TCA cycle and enhancing chromatin accessibility, lactate-dependent H3K27la upregulates cytokine expression and strengthens innate immune memory, linking metabolic flux to long-term epigenetic reprogramming in monocytes.

Beyond experimental models, clinical evidence also supports the diagnostic relevance of histone lactylation in human immune responses. In a prospective cohort study of patients with sepsis, Wang et al. (2025) demonstrated that neutrophil H4K5la and T-cell H3K56la levels are markedly elevated during acute infection and correlate with immune activation. Importantly, neutrophil H4K5la on day 1 served as an independent predictor of sepsis, and its combination with C-reactive protein (CRP) significantly improved diagnostic accuracy. Conversely, decreased lactylation on day 3, particularly reduced Pan Kla and H3K56la, was associated with unfavorable outcomes, suggesting that immune cell lactylation may serve as a dynamic biomarker for disease progression and prognosis. Histone lactylation and its various biological functions are summarized in Table 1. The table presents a comprehensive overview of the representative histone lactylation marks and their reported roles in different cellular processes.

**Table 1:** Representative histone lactylation marks and their reported biological functions

Histone site	Biological context	Reported function	Reference
H3K18la	M2 macrophage polarization	Activate Arg1 gene expression and promote tissue repair	[15, 84]
H3K14la	Intracerebral hemorrhage	Upregulate PMCA2, impair Ca²⁺ efflux, and exacerbate neuronal ferroptosis	[90]
H3K9la	HNSCC	Upregulate IL−11 via JAK2/STAT3, induce CD8⁺ T-cell exhaustion, and promote immune escape	[91]
H3K23la	Mouse oocytes and pre-implantation embryos	Display oxygen and LDHA-dependent dynamics that regulate embryonic developmental potential	[85]
H3K27la	Trained monocytes/innate immune memory	Enhance chromatin accessibility and cytokine production by fueling the TCA cycle and promoting histone lactylation, linking lactate metabolism to trained immunity	[254]
H4K12la	Alzheimer’s Disease	Promote glycolysis-related gene transcription (such as HIF−1α, PKM, and LDHA), elevate glycolytic flux, worsen microglial dysfunction in Alzheimer’s disease, and drive neuroinflammation and cognitive impairment via the glycolysis-H4K12la-PKM2 feedback mechanism.	[255]
H4K5la/H3K56la/Pan Kla	Immune cells from patients with sepsis	Correlate with immune activation and clinical outcome; neutrophil H4K5la predicts sepsis progression, while reduced H3K56la and Pan-Kla associate with poor prognosis	[256]
H4K5la/H4K8la/H4K12la	Male germ cells during meiotic prophase I	Orchestrate meiosis by marking recombination hotspots and cohesin sites to activate gene expression	[89]

This table summarizes experimentally validated histone lysine lactylation (Kla) sites, their biological contexts, and corresponding functional outcomes. Lactylation marks are implicated in diverse physiological and pathological processes, including immune regulation, neuronal injury, early embryogenesis, innate immune memory, infection response, and reproductive development

*Abbreviations*:*HNSCC* Head and neck squamous cell carcinoma, *PMCA2* Plasma membrane calcium-ATPase 2, *AD* Alzheimer’s Disease, *HIF*-*1α* Hypoxia-Inducible Factor 1-alpha, *PKM* Pyruvate Kinase M Isozyme, *JAK2* Janus kinase 2, *STAT3* Signal transducer and activator of transcription 3, *IL*-*11* Interleukin-11, *LDHA* Lactate dehydrogenase A, *TCA* Tricarboxylic acid, *Pan Kla* Pan-lysine lactylation

#### Functional roles of non-histone lactylation

**Fig. 2:**
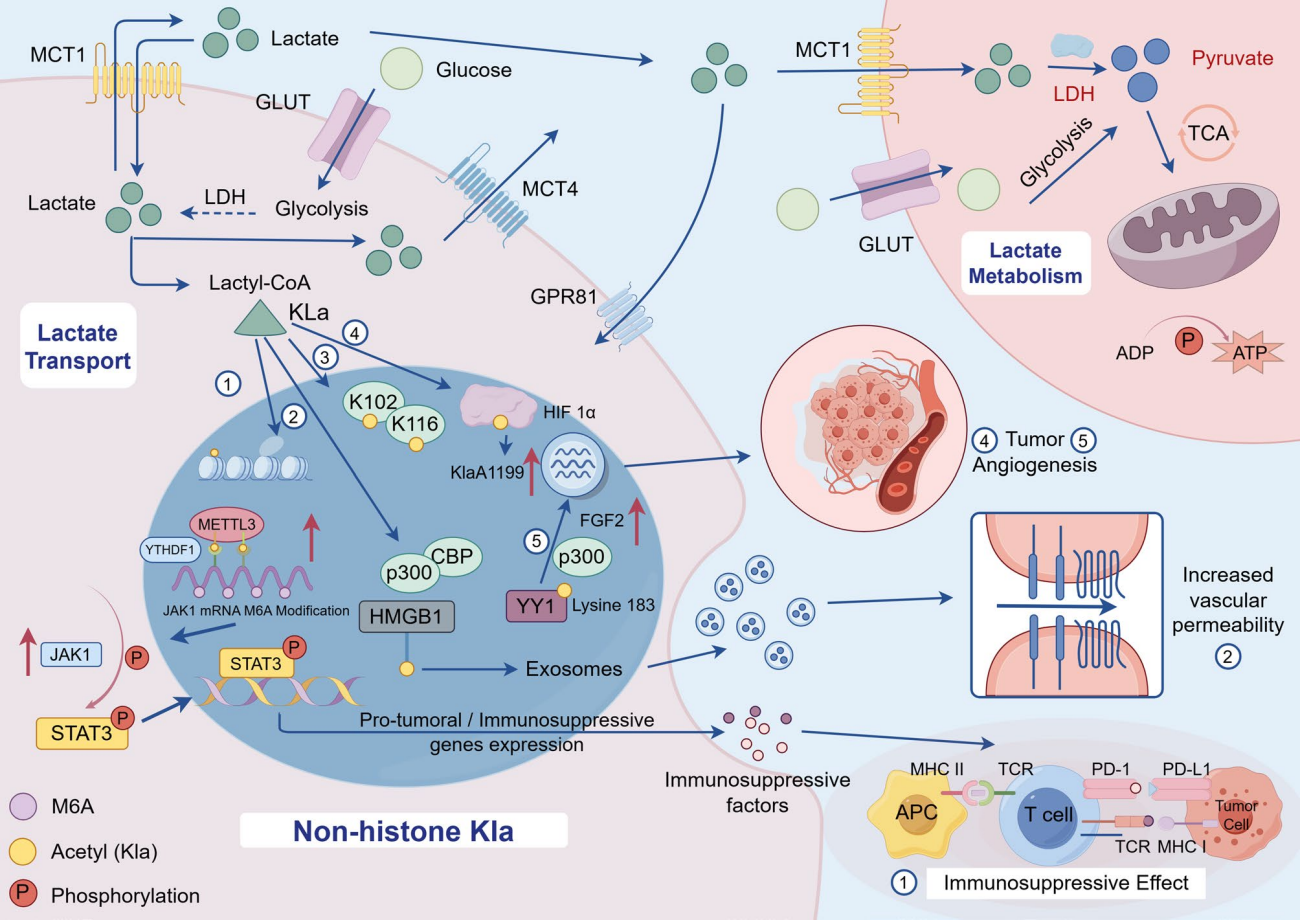
Mechanism of lactate transport and non-histone protein lactylation with functional outcomes. During glycolysis, lactate is generated via lactate dehydrogenase (LDH) and exported to the extracellular space through MCT4. Under normoxic conditions, adjacent cells import lactate via MCT1, where it is utilized for oxidative metabolism or functions as a signaling mediator. (1) Lactate-derived lactyl-CoA facilitates lysine lactylation on non-histone proteins, which promotes METTL3-mediated m6A methylation of JAK1 mRNA and activates STAT3 phosphorylation, enhancing the immunosuppressive phenotype of tumor-infiltrating myeloid cells (TIMs) and promoting immune evasion. (2) In a p300/CBP-dependent context, lactate induces HMGB1 lactylation, leading to its exosomal secretion and contributing to vascular endothelial dysfunction and the progression of sepsis. (3) Nucleolin (NCL) undergoes lactylation at K102 and K116, with this modification correlating with intracellular lactate levels and affecting rRNA transcription and ribosome biogenesis. (4) Lactate stabilizes HIF-1α through lactylation following MCT1-mediated uptake under normoxia, thereby enhancing KlAA1199 expression and promoting angiogenesis. (5) Lactate also induces YY1 expression and FGF2 transcription, amplified via p300-mediated K183 lactylation of YY1, which contributes to pathological angiogenesis. The immunosuppressive consequence is illustrated by the PD-1/PD-L1 axis as a representative checkpoint pathway. *Abbreviations*: *LDH* Lactate dehydrogenase, *MCT1*/*4* Monocarboxylate transporter 1/4, *GLUT* Glucose transporter, *GPR81* G protein-coupled receptor 81, *Kla* Lysine lactylation, *METTL3* Methyltransferase-like 3, *m6A* N6-methyladenosine, *JAK1* Janus kinase 1, *STAT3* Signal transducer and activator of transcription 3, *HMGB1* High mobility group box 1, *CBP/p300* CREB-binding protein/E1A binding protein p300, YY1, Yin Yang 1, *FGF2* Fibroblast growth factor 2, *K183* Lysine 183, *TCA* Tricarboxylic acid cycle, *PD*-*1/PD-L1* Programmed death-1/Programmed death ligand 1, *MHC* Major histocompatibility complex, *TCR* T-cell receptor, *APC* Antigen-presenting cell, *ATP/ADP* Adenosine triphosphate/Adenosine diphosphate

Beyond chromatin regulation, lactylation has been increasingly recognized on a broad spectrum of non-histone proteins, extending the influence of lactate from epigenetic control to post-translational signaling. These modifications alter protein conformation, stability, and intermolecular interactions, thereby impacting inflammation, metabolism, and tumor progression. One well-characterized non-histone substrate is HMGB1, a chromatin-binding protein with extracellular cytokine-like activity. Through p300/CBP-dependent lactylation, lactate promotes the nuclear-to-cytoplasmic translocation of HMGB1, which is subsequently secreted in exosomes and contributes to endothelial dysfunction and sepsis progression [80]. This finding reveals that lactate-mediated modifications can reprogram immune and inflammatory responses independently of chromatin remodeling. Further evidence highlights the metabolic sensitivity of non-histone lactylation. Nucleolin (NCL) exhibits lactylation at K102 and K116, correlating with cytoplasmic lactate levels and affecting its functions in rRNA transcription and ribosome biogenesis [92]. Likewise, YY1 lactylation at K183, mediated by p300, enhances FGF2 transcription in retinal microglia, promoting pathological angiogenesis (Fig. 2) [79]. In addition, lactate-induced lactylation stabilizes HIF-1α under normoxic conditions, facilitating its transcriptional activity on angiogenic targets (KlAA1199 axis) in prostate cancer cells and contributing to vascular remodeling [93].

Global proteomic analyses have revealed that non-histone lactylation is widespread, particularly in the cytoplasm of hepatocellular carcinoma cells, where it regulates intracellular signaling and metabolic enzyme activity [77]. Importantly, delactylase enzymes, such as HDAC1, HDAC3, and SIRT family members, have been shown to remove lactyl groups in vitro, suggesting that non-histone lactylation is dynamically reversible and may constitute a regulatory network parallel to acetylation [94]. However, the substrate specificity, compartmental localization, and physiological relevance of these delactylases in vivo remain incompletely understood, and no specific “reader” proteins have yet been identified to interpret lactylation marks.

Functionally, non-histone lactylation has been implicated in immunoregulation. In macrophages, for example, lactylation may reinforce M2-like polarization, likely complementing the Arg1-driven mechanism previously discussed [95]. More broadly, lactylation appears to promote the expression of anti-inflammatory genes and facilitate immune evasion, thereby contributing to an immunosuppressive microenvironment. These findings suggest that targeting the lactylation machinery may offer therapeutic potential. For instance, HDAC inhibitors, some of which possess delactylase activity, may reverse lactylation-associated immune suppression and restore anti-tumor immunity. Although direct preclinical evidence remains limited, this represents a promising direction for future investigation. Moreover, lactate-driven immunosuppressive signaling also extends beyond covalent modifications, such as through engagement with GPR81 and other metabolic sensors [15, 80, 96]. Taken together, lactate-induced non-histone lactylation represents a critical metabolic interface that bridges glycolytic activity with protein function, immune remodeling, and cellular signaling.

Moreover, lactate-associated metabolic signaling extends beyond covalent modification. Ippolito et al. (2020) reported that fibroblasts infiltrating the TME secrete lactate to shape lipid metabolic circuits through histone acetylation, highlighting how carbon flux and acyl-donor dynamics can converge on chromatin remodeling [97]. These findings underscore the broader role of metabolite-driven protein modifications in immune and metabolic homeostasis. Nevertheless, key questions remain regarding the enzymatic control, cell-type specificity, and in vivo relevance of lactylation, warranting deeper mechanistic and translational investigation. Non-histone lactylation and its functional roles are summarized in Table 2, which outlines the validated and candidate non-histone lactylation substrates along with their enzymatic regulation.

**Table 2:** Non-histone lactylation substrates and enzymatic regulation

Protein	Lactylation site	Biological context	Functional outcome	Associated enzymes	Evidence type	Reference
HMGB1	Lys (unspecified)	Endothelial cells/sepsis	p300-dependent lactylation drives nuclear export and exosomal secretion; promotes endothelial dysfunction and inflammation	Writer: p300/CBP	In vitro + in vivo	[80]
NCL	K102, K116	Human cell lines	Lactylation correlates with cytoplasmic lactate levels; modulates rRNA transcription and ribosome biogenesis; links metabolic state to nucleolar function	Writer: unknown (presumably p300)	In vitro + in vivo	[92]
YY1	K183	Retinal microglia	p300-mediated lactylation enhances FGF2 transcription and pathological angiogenesis	Writer: p300	In vitro + in vivo	[79]
HIF-1α	KlAA1199	Prostate cancer cells (normoxia)	Lactate imported via MCT1 induces HIF1α lactylation and stabilization, leading to transcriptional activation of KIAA1199 and pro-angiogenic genes	Writer: not specified (lactate-dependent)	In vitro + in vivo	[93]
Adenylate kinase 2	K28	HBV-related HCC	Kla at K28 inhibits AK2 enzymatic function, thereby promoting proliferation and metastasis of HCC cells	Writer: p300; Eraser: HDAC3	Global lactylome	[77]

Summary of experimentally validated and putative non-histone lactylation targets, their modification sites, biological contexts, associated enzymes, and functional implications across inflammatory, metabolic, and tumor models

*Abbreviations*: *HMGB1 *High-mobility group box 1, *NCL* Nucleolin, *YY1* Yin Yang 1, *HIF*−1α Hypoxia-inducible factor-1 alpha, *HBV* Hepatitis B virus, *HCC* Hepatocellular carcinoma, *FGF2* Fibroblast growth factor 2, *MCT1* Monocarboxylate transporter 1, *HDAC* Histone deacetylase

## Lactate and immunity

### Myeloid immune cells

Myeloid-derived suppressor cells (MDSCs) are crucial components of the innate immune system, with the ability to differentiate into various immune cell types, including macrophages, dendritic cells (DCs), and mast cells [98]. Emerging evidence suggests that lactate exerts metabolic effects beyond its conventional role within these immune lineages, and also plays a multifaceted regulatory role. Specifically, lactate is actively involved in immunoregulatory processes through metabolic crosstalk and cytokine-mediated signaling pathways [98, 99].

In addition to these effects, lactate also functions as a signaling molecule that activates MDSCs via the GPR81 receptor through the mTOR/HIF-1α/STAT3 axis. This lactate-induced activation of MDSCs plays a crucial role in promoting an immunosuppressive phenotype, which significantly impairs the effectiveness of anti-tumor immune responses [100]. Furthermore, metabolic reprogramming in tumor cells, marked by heightened lactate secretion, is linked to tumor progression and immune suppression in the TME. Lactate influences immune cell activity, including macrophages, DCs, and T cells, fostering an immunosuppressive milieu. Resistance to anti-PD-1 (programmed death-1)/PD-L1 (programmed death-ligand 1) therapy has been associated with lactate accumulation in the TME [101, 102].

Subpopulations of macrophages (M0, M1, and M2) are capable of dynamic interconversion. The M0 phenotype can be transformed into M1 or M2 subtypes depending on external stimuli. M1 and M2 subpopulations generally exhibit opposing functions: M1 macrophages release pro-inflammatory cytokines, while M2 macrophages display an anti-inflammatory phenotype involved in tissue repair and healing [25]. The interplay between lactate and macrophages exemplifies this plasticity. For instance, lactate induces M2 macrophage activation via MCT1 signaling in muscle and metabolic reprogramming in mesenchymal stem cell (MSC) secretion, promoting tissue repair and growth [103, 104].

However, this pro-repair, anti-inflammatory outcome represents only one facet of lactate’s broad functional spectrum. Recent evidence, particularly from single-cell studies, underscores that lactate’s influence is highly context-dependent and extends beyond M2 polarization, potentially sustaining inflammatory functions. This dual role is supported by distinct mechanistic insights. Beyond its role in promoting M2 polarization, lactate can be utilized by macrophages as a metabolic fuel in glucose-deprived environments, such as tumors, to sustain their energy-demanding pro-inflammatory functions [105]. The discovery of histone lactylation further reveals lactate’s capacity to directly reshape macrophage gene expression. In LPS-activated M1 macrophages, lactate-derived lactylation initiates a unique genetic program that blurs the line between classical polarization states, driving the expression of both pro-inflammatory and homeostatic genes [15]. This model of metabolic feedback is also observed in antiviral immunity, where lactate suppresses type I interferon production by lactylating the key signaling protein MAVS, revealing an unconventional immunosuppressive function [106]. Furthermore, in the tumor microenvironment, lactate can polarize macrophages toward a pro-angiogenic phenotype characterized by vascular endothelial growth factor (VEGF) production, which facilitates tumor progression [107].

In summary, the role of lactate in macrophages cannot be oversimplified as merely “promoting M2.” It functions more like a “double-edged sword,” with its ultimate effect, whether it favors pro-inflammatory (M1) or anti-inflammatory (M2) phenotypes, being tightly regulated by factors such as concentration, duration, cell origin, and the overall tissue microenvironment [108]. Future research should explore these novel perspectives to further uncover the specific molecular switches that determine the functional shift of lactate.

As professional antigen-presenting cells, DCs exhibit exceptional efficiency and immunological potency. Most importantly, the significance of DCs is being recognized in multiple fields, an exemplary case is the application of dendritic cell vaccines [109]. Nasi A et al. proposed a novel strategy of modulating DCs vaccines immunogenicity, by means of lactate-induced PPARγ pathway modification resulting in inhibitory effects on DCs maturation [110]. Moreover, in accordance with what has been discussed above, the lactate secreted by MSCs can not only induce M2 macrophage polarization, but promote the capability of DCs polarizing CD4^+^T cells into Th2 cells, implicating the dynamic interaction between M2 macrophages and DCs [104].

Mast cells, unique subpopulations of myeloid lineage-derived immune cells, were once perceived as effectors in allergic responses or autoimmune diseases while gradually being reconsidered as a crucial mediator participating in complicated pathophysiological processes, which is related to monocyte-macrophage system [111]. Given the observation that lactate was enriched in the inflamed tissues, researchers believed it to be an inhibitor of inflammatory cytokines production and degranulation of mast cells, dependent on MCT1 expression [112]. Lactate also regulates mast cell activation through MRGPRX2, suppressing both early (Ca2^+^ mobilization, degranulation) and late (cytokine/chemokine release) phases of activation [113]. Moreover, Abebayehu D et al. showed that lactate could reduce IgE and IL-33 production in an MCT1-dependent manner, involving miR-155-5p downregulation likely via HIF-1α regulation, reinforcing lactate’s role in controlling mast cell functions in inflammation [114].

Lactate has also been shown to reduce IgE-induced Syk, Btk, and ERK phosphorylation, key signals of inflammation, and inhibit passive systemic anaphylaxis in mice. In vivo, lactate injection reduced IgE-mediated hypothermia in a murine model of anaphylaxis. Furthermore, lactate suppressed the activation of MRGPRX2/MrgprB2 receptors in both human and mouse mast cells, suggesting its potential to modulate mast cell responses during allergic diseases [112].

### CD4^+^/CD8^+^/Treg T lymphocytes

T lymphocytes, encompassing both CD4⁺ helper and CD8⁺ cytotoxic subsets, are the main differentiating subpopulations of naïve T cells, which possess heterogeneous populations and follow distinct differentiation trajectories [115]. Recent studies have shown that lactate exerts differential regulatory effects on CD4^+^ and CD8^+^ T lymphocytes. Moreover, emerging evidence underscores that lactate’s role in T cell immunity is context-dependent, exhibiting both inhibitory and supportive functions.

Crucially, the expression of lactate transporters varies between T cell subsets and is influenced by the immunological context, which underpins these divergent functional outcomes. In CD4⁺ T cells, extracellular lactate not only inhibits their migratory capacity, but also contributes to their conversion to a proinflammatory Th17 phenotype, characterized by increased expression of cytokines such as IL-2 and IL-17 [116, 117]. Lactate uptake in CD4⁺ T cells can be mediated by MCT1, and notably, by the sodium-coupled monocarboxylate transporter 2 (SMCT2) in specific contexts, which in turn affects intracellular glycolytic activity [116]. Conversely, under specific conditions such as the TME, lactate robustly promotes the activation, expansion, and suppressive role of regulatory T cells (Tregs), a pivotal mechanism in the establishment of immunosuppression [118]. This effect is largely dependent on lactate uptake via MCT1, which enables Tregs to utilize extracellular lactate as an alternative fuel under glucose-deprived conditions. The lactate-driven metabolic reprogramming enhances mitochondrial oxidative phosphorylation and sustains Foxp3 expression, thereby reinforcing the immunosuppressive phenotype of Tregs. In addition, lactate-mediated histone lactylation has been implicated in stabilizing Treg lineage commitment and functional persistence, further contributing to immune tolerance within TME [118].

The effects on CD8^+^ T cells are equally dichotomous. While lactate impairs the motility and cytolytic function of effector CD8^+^ T cells, it does not inhibit glycolysis in these cells, unlike in CD4^+^ T cells, yet still compromises their mobility, thus blunting their cytolytic function [116]. In CD8^+^ T cells, MCT1 serves as the primary lactate importer, mediating these inhibitory effects [116]. Similar studies confirmed that CD8^+^ T cells in a lactate-rich environment exhibited reduced motility, suppressing degranulation and cytotoxin secretion by decreasing the abundance of effector CTLs at multiple steps. Owing to impaired maneuverability, CTLs are more likely to either fail to effectively engage with targeted tumor cells or have difficulty detaching from them, leading to prolonged contact, both of which hinder the effectiveness of tumor elimination.

Recent findings indicate that lactate supports the survival and persistence of CD8^+^ memory T cells, especially under nutrient stress. This includes conditions such as exercise or acute infection. This effect is mediated through both metabolic and epigenetic mechanisms. Due to their relatively low glycolytic activity, memory CD8^+^ T cells rely more heavily on exogenous lactate uptake via MCT1, which sustains intracellular lactate levels and promotes histone H3K9 lactylation (H3K9la). This modification is enriched at promoter regions of genes involved in OXPHOS, fatty acid oxidation, and memory-associated factors, contributing to the long-term functional maintenance of memory T cells. Through this mechanism, lactate enhances mitochondrial function and metabolic reprogramming, which are essential for the endurance and longevity of memory CD8^+^ T cells. Moreover, H3K9la-mediated transcriptional activation supports effector potential retention and adaptation to metabolic stress. These findings collectively suggest that lactate acts not only as an alternative energy substrate but also as a key epigenetic modulator, enabling CD8^+^ memory T cells to persist and function effectively over extended periods [119].

**Fig. 3:**
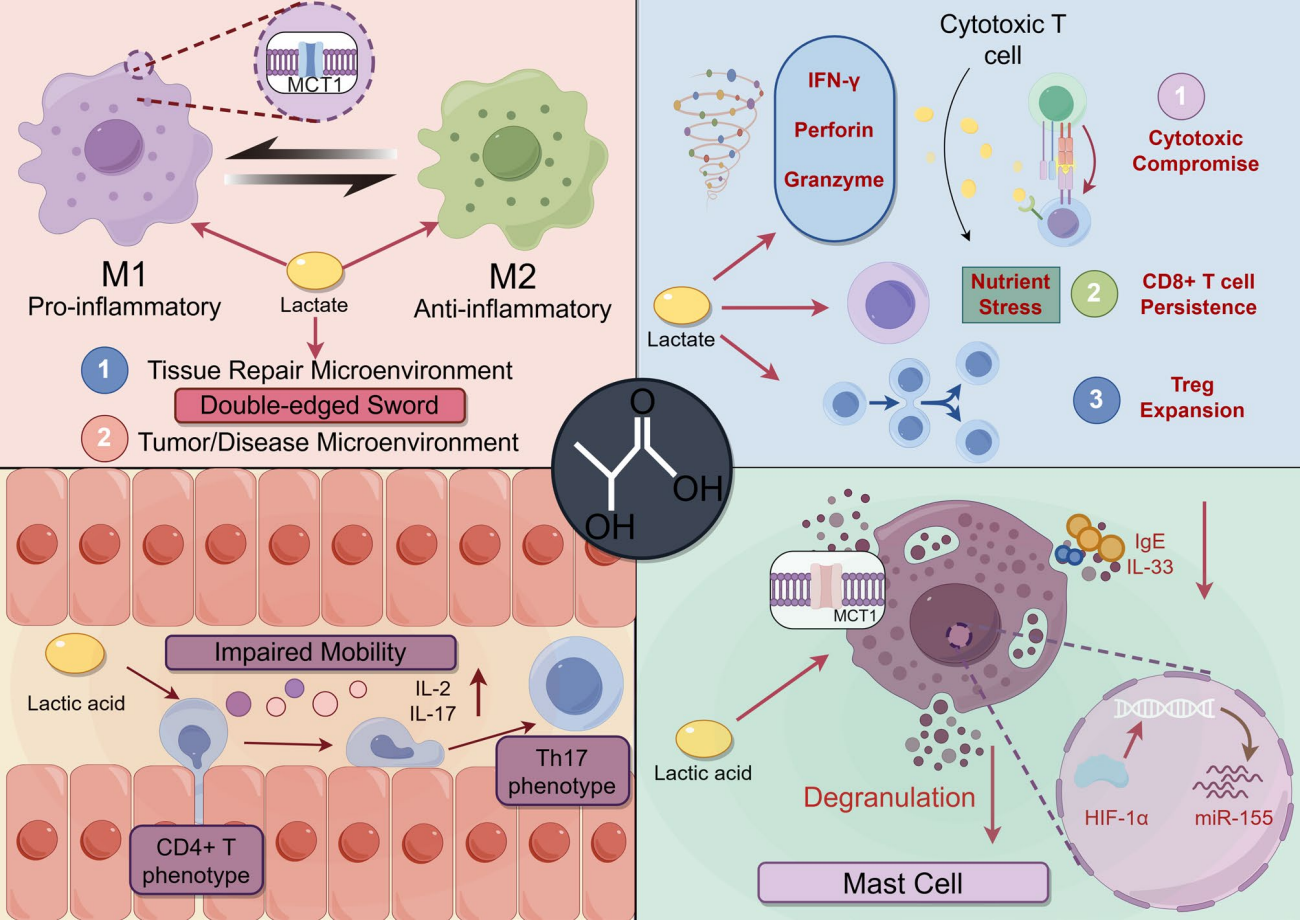
The immunomodulatory effects of lactate across distinct immune cell types. Lactate exerts a complex and context-dependent dual influence on immune cells. In macrophages, lactate acts as a double-edged sword: in a tissue repair microenvironment, it promotes an M2-type anti-inflammatory phenotype via MCT1 signaling and metabolic reprogramming, facilitating inflammation resolution; conversely, in a tumor/disease microenvironment, it can serve as a metabolic fuel, thereby sustaining an M1-like pro-inflammatory phenotype or driving a pro-angiogenic phenotype that contributes to disease progression. In T cells, lactate displays a functional dichotomy: while it inhibits effector functions of CD8^+^ cytotoxic T cells by decreasing the expression of IFN-γ, perforin, and granzyme as well as promoting regulatory T cells (Tregs) expansion, it also supports the persistence of CD8⁺ memory T cell precursors under nutrient stress. In CD4⁺ T cells, lactate limits cellular mobility and promotes IL-2 and IL-17 secretion to drive a pro-inflammatory Th17 phenotype; conversely, it also promotes the expansion and suppressive function of Tregs, a key mechanism in immunosuppression. Furthermore, lactate inhibits mast cell degranulation and cytokine release by entering cells through MCT1. This process involves suppression of the HIF-1α/miR-155 signaling axis and is associated with reduced IL-33-mediated inflammatory responses. *Abbreviations*: *M1/M2* M1 type/M2 type macrophages, *IFN*-γ Interferon gamma, *IL-2/IL-17* Interleukin 2/17, *CD4⁺/CD8⁺ T cells* Cluster of differentiation 4/8 positive T lymphocytes, *Tregs* Regulatory T cells, *MCT1* Monocarboxylate transporter 1, *HIF-1α* Hypoxia-inducible factor 1-alpha, *miR*-*155* MicroRNA-155, *IgE* Immunoglobulin E, *IL*-*33* Interleukin 33

Therefore, reduced cytotoxic ability associated with limited killing scope is likely to reinforce the conceptual assumption that lactate exhibits remarkable potential to coordinate the spatial distribution and modulate the biological functions of CD8^+^ T cells [120](Fig. 3). Collectively, lactate acts as a metabolic checkpoint that can simultaneously suppress effector T cell functions while fostering immunosuppressive Tregs and, paradoxically, supporting the foundation of long-term immunity via memory CD8⁺ T cells, highlighting a sophisticated and context-dependent immunomodulatory profile.

## Lactate and disease

### Immunity-related diseases

Immune responses intersect with a diverse range of disease occurrences. Apart from being involved in metabolic activities, lactate has exhibited remarkable potential in immune regulation [99]. Targeting specialized immune cell populations with high heterogeneity, lactate interacts with them in a dynamic manner. Inflammatory diseases are often accompanied by immune system disorders when lactate metabolism is disrupted. Therefore, to deepen the understanding of lactate’s diverse immunoregulatory roles across different immune cell populations involved in immune-related diseases, we aim to provide a detailed explanation in this section.

#### Rheumatoid arthritis

For the past few decades, the parallel relationship between lactate and rheumatoid arthritis (RA) has been recognized owing to lactate enrichment in synovial cells, endowing it with the trait of being a reliable indicator for differentiating RA [17]. This phenomenon suggests enhanced glycolytic metabolic tendency in synovial cells. Peng et al. proposed aerobic glycolysis-dominant metabolic pathway with responsive LDHA concentration increase in activated T cells, releasing IFN-γ and maintaining histone acetylation to promote effector T cell functions [121]. However, constrained CD4^+^/CD8^+^ T cells motility were also observed for different reasons when extracellular lactate concentration increased, mediated by SMCT2 on CD4⁺ T cells and MCT1 on CD8⁺ T cells, and was retrospectively shown to correlate with T cell function in RA synovia [116].

Consequently, the impaired migration ability caused CD4^+^/CD8^+^ T cells’ prolonged retention in synovial fluids, where lactate could promote CD4^+^ T cells to transform into Th17 subsets producing abundant IL-17, and to reduce the cytolytic capacity in CD8^+^ T cells, possibly owing to autoantibody and ectopic lymphoid structures [122]. Additionally, the decreased pH running parallel to the RA activity is facilitated by MCT4, which exports lactate from synovial fibroblasts (RASFs) [122]. Compelling preclinical evidence positions MCT4 as a key therapeutic target. In RA patients, MCT4 is upregulated in RASFs, directly contributing to synovial fluid acidification. Crucially, in a mouse model of collagen-induced arthritis, targeted silencing of MCT4 within the articular synovium significantly attenuated disease severity by inducing apoptosis in RASFs, offering direct proof-of-concept for this approach [122].

Furthermore, considering the specific transporter SMCT2, highly expressed on CD4^+^ T cells, this biological characteristic provides an essential influx route for lactate ingestion. Once intracellular lactate is sufficient to induce CD4^+^ T cell phenotype transformation, RORγT-dependent IL-17 expression via activation of the PKM2-STAT3 axis would be initiated, confirming lactate as a critical mediator in inflammation-related diseases, including RA. Moreover, in correspondence with what was delineated above, the extracellular lactate accumulation would lead to localized CD4^+^ T cell retention in inflamed lesions due to inhibited motility, as a consequence of glycolytic activity inhibition [123]. Haas R et al. further elaborated on this phenomenon: CD4^+^ T cell movement limitation was found to involve the CXCR3/CXCL10 axis, while CD8^+^ T cell motility does not apply to this regulatory mechanism, presumably due to the loss of cytolytic effectiveness [116].

**Fig. 4:**
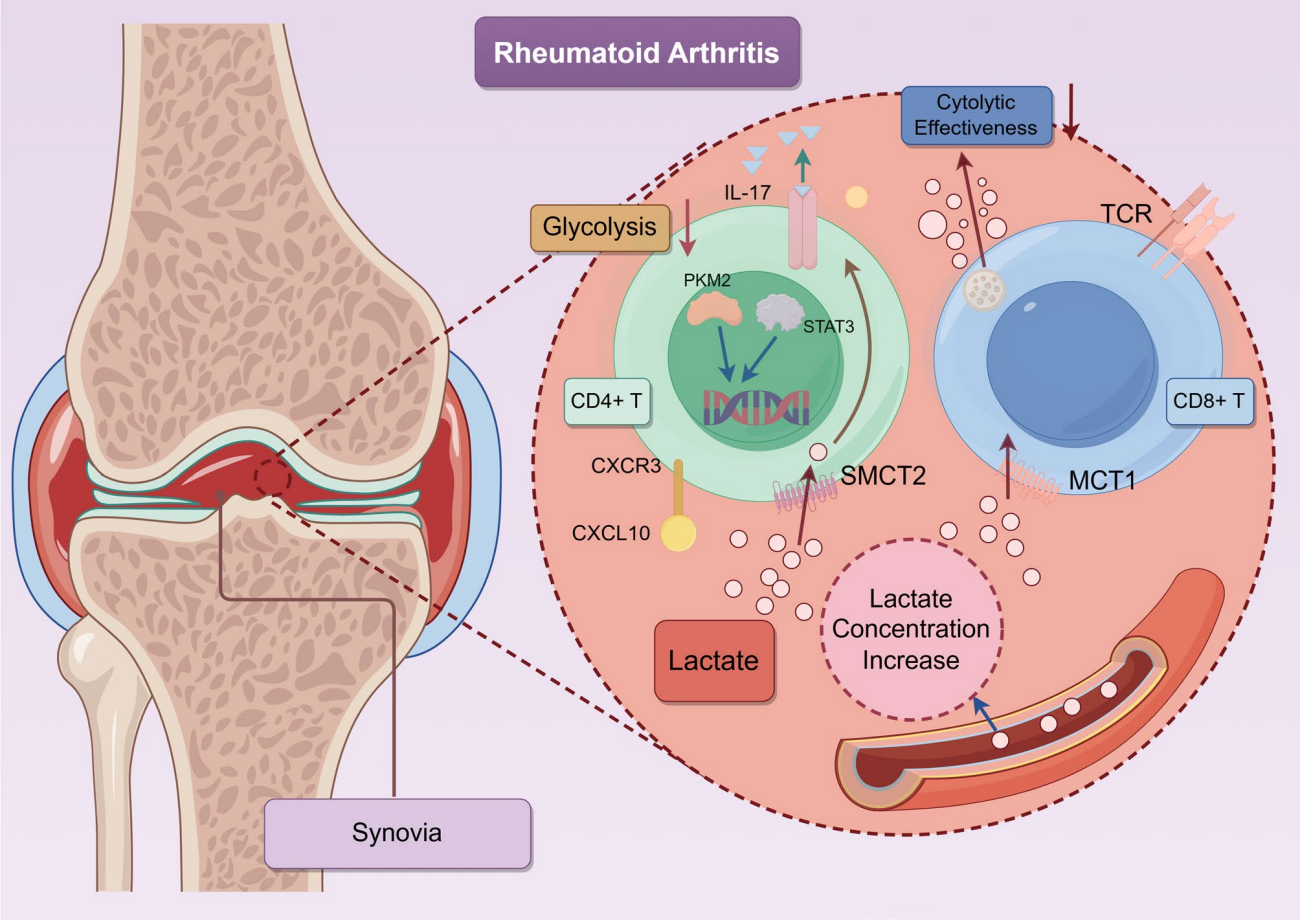
Lactate accumulation in the RA synovium leads to increased expression of MCT1 on CD8^+^ T cells and SMCT2 on CD4^+^T cells, mediating intracellular lactate uptake. In the context of CD4 + T cells, lactate uptake activates the PKM2/STAT3 signaling pathway and promotes IL-17 production, driving Th17 polarization and contributing to synovial inflammation. In contrast, lactate impairs the cytolytic effectiveness of CD8^+^T cells, potentially through MCT1-mediated transport. Additionally, the CXCR3/CXCL10 chemokine axis may contribute to CD4^+^T cell retention in the inflamed synovial tissue, further exacerbating chronic inflammation. Lactate thus acts as a metabolic and migratory regulator in RA pathogenesis. *Abbreviations*: *RA* Rheumatoid arthritis, *IL*-*17* Interleukin-17, *CD4*⁺/*CD8*⁺ *T* Cluster of differentiation 4/8 positive T cells, *MCT1* Monocarboxylate Transporter 1, *PKM2* Pyruvate Kinase M2, *STAT3* Signal transducer and activator of transcription 3, *CXCR3* C-X-C chemokine receptor type 3, *CXCL10* C-X-C motif chemokine ligand 10

Therefore, both T cells and lactate are closely correlated with chronic inflammation, revealing the detailed clues comprising the delicate lactate interaction with cellular metabolism and behavioral adjustments (Fig. 4).

#### Ulcerative colitis

Ulcerative colitis (UC), a chronic bowel disease with immune disorders involving the colon and rectum, has contributing pathogenic factors characterized by individual heterogeneity, such as environmental factors, genetic susceptibility, and immune imbalances. Since the efficacy of TNF-α treatment was demonstrated in clinical trials in the 2000 s, the UC management strategy has evolved into a paradigm shift towards healing at the histological level, aiming to improve patient diagnosis and quality of life as much as possible [124].

Admittedly, gut microbiome disorder is one of the underlying causes of UC. Dietary management based on anti-inflammatory principles has also recently been utilized as a recommended therapy to reduce immunosuppressive agent-induced adverse effects [125]. Thus, considering the high abundance of lactate production mostly derived from specific gut bacterial communities such as bifidobacteria and lactobacilli, it is worthwhile to analyze the complex and context-dependent roles of lactate during UC-related gut microbiome disorders [126]. Mechanistically, studies in mouse colitis models by Ranganathan et al. identified GPR81 on colonic dendritic cells and macrophages as a key suppressor of inflammation, promoting Tregs and limiting Th1/Th17 responses. These findings suggest GPR81 is a compelling preclinical target. However, its clinical translatability requires evaluation, particularly given the complex, context-dependent role of lactate in human UC, where its accumulation can also be pathogenic [127]. In conjunction with this finding, intrarectal lactate administration induced symptom relief in a murine colitis model. The possible explanations include the potential to reduce serum IL-6 levels, disrupting metabolic activities correlated with proinflammatory cytokine release [128]. Of note, on the basis of epigenetic modification, another study contextualized the significance of lactate-promoting effects on histone H3K18 lactylation in association with histone H3K9 acetylation in UC management, by increasing MCT-induced macrophage lactate uptake, suppressing NOD-like receptor family pyrin domain containing 3 (NLRP3) inflammasome activation, and reducing macrophage pyroptosis induced by caspase-1 expression [129].

However, the beneficial, anti-inflammatory role of lactate contrasts with observations in human UC patients. Metagenomic and metabolomic analyses of human gut microbiota have revealed that microbial dysbiosis can lead to excessive lactate accumulation in the colonic lumen. This overabundance of lactate is associated with a worsened disease state, potentially by creating a pro-inflammatory microenvironment, disrupting the balance of short-chain fatty acid production, and directly impairing epithelial barrier function [130]. Therefore, as evidenced by current observations, lactate metabolism may possess a dual immunomodulatory propensity that is tightly regulated by the gut microbial ecology and metabolic context. The impact of lactate, whether beneficial or harmful, depends on various factors, including its concentration, origin, and the overall condition of the host’s microbiome and immune system.

#### Allergic diseases

Given the observation that lactate plasma concentration in patients with asthma increases in conjunction with disease severity, the relationship between lactate and asthma has drawn widespread attention. Irvin C et al. found that significant enrichment of Th2 and Th17 lymphocyte subsets identified within bronchoalveolar lavage samples obtained from individuals diagnosed with asthma could trigger IL-4 overproduction and subsequently augment the capability of inducing glucocorticoid resistance in vitro [131]. Given that lactate could reinforce this through delicate immunomodulatory propensity, it could help explain its corresponding roles in allergy.

However, another immune cell subpopulation closely associated with allergic reactions is mast cells, as mentioned above. When stimulated by lactate administration, mast cells tend to reduce the production of both IgE and IL-33 cytokines [114]. This finding indicates the therapeutic potential of lactate in alleviating allergic responses through inhibiting the release of endogenous sensitizing substances.

To summarize, immunometabolic activities are of great significance in allergic diseases. Lactate possesses the propensity to alter allergy-associated immune cell phenotypes from intrinsic perspectives, helping to maintain or disrupt immune balance.

### Cardiovascular disease

As a crucial energy substrate, lactate utilization covers a large proportion of the energy in cardiomyocytes. The clinical relevance of lactate in myocardial infarction (MI) is firmly established in human studies, which consistently show that elevated blood lactate levels serve as a powerful prognostic indicator, being strongly associated with higher mortality and poorer outcomes in patients with acute MI [132]. However, the dual role of lactate is evident as it also contributes to the progression of myocardial fibrosis and worsens cardiac dysfunction by facilitating endothelial-mesenchymal transition (EndoMT) following MI [133].

The functional significance of lactylation at the α-MHC K1897 site in regulating cardiac contractility was first characterized in mouse models. Proteomic analysis revealed decreased K1897 lactylation in failing hearts. Mechanistically, mice engineered with an α-MHC K1897R substitution mutation exhibited impaired α-MHC-Titin interaction and exacerbated cardiac dysfunction. Importantly, increasing intracellular lactate levels, either by lactate administration or inhibition of the lactate exporter MCT4, alleviated cardiac dysfunction in these models, suggesting a potential therapeutic mechanism mediated by K1897 lactylation [18].

While lactylation is an evolutionarily conserved modification, the existence and functional role of this specific α-MHC K1897 lactylation event in human cardiomyocytes remain unvalidated and represent a critical area for future research. Beyond its role in modifying structural proteins like α-MHC, lactylation also functions as an epigenetic mark. For instance, it modifies key transcription factors and histones, such as H3K27, which is closely correlated with gene expression levels in end-stage heart failure [134]. This broader regulatory role enables lactylation to coordinate the expression of genes involved in myocardial repair following injury [18, 135].

Based on this assumption, and considering that lactate depletion may aggravate myocardial dysfunction, research has explored whether MCT4 inhibitors could reverse ventricular remodeling at early onset. Coinciding with this assumption, Cluntun et al. found that inhibiting lactate export could also ameliorate hypertrophic phenotype transformation mediated by the pyruvate-lactate metabolic shuttle axis. Given the myocardial propensity to consume exogenous Cori cycle-produced lactate, lactate consumption in cardiomyocytes is normally balanced with the rate of glycolytic pyruvate-derived lactate production, commonly known as the pyruvate-lactate axis. The circulatory abundance fluctuation between lactate and pyruvate is a decisive factor contributing to cardiomyocyte maintenance. However, this axis balance is disrupted when cardiomyocytes prefer pyruvate-derived lactate production, accompanied by a concomitant decrease in lactate consumption at the onset of heart failure [136]. This study identified both the MPC and MCT4 as central nodes in this metabolic intersection. MCT4 blockade (VB124) administration was then tested for its capacity to mitigate hypertrophy in cultured mouse cardiomyocytes [137]. In murine models of type 2 diabetes, MCT4 inhibition alleviated myocardial oxidative stress and pathological injury. It is crucial to note, however, that recent studies highlight significant systemic side effects of MCT4 inhibition due to its broad tissue expression, which may limit its clinical translational potential and necessitate the development of tissue-specific delivery strategies [138].

However, it is important to note that recent studies on MCT4 inhibition have revealed significant systemic side effects that may limit its clinical translational potential. Specifically, MCT4 is widely expressed in various normal tissues, where it plays a crucial role in lactate efflux. Its systemic inhibition could lead to lactate acidosis, metabolic disturbances, and impaired immune function, among other adverse events [68]. Therefore, while MCT4 plays an important role in regulating myocardial metabolism, developing tissue-specific targeting methods or seeking combination therapies may be key strategies to overcome these side effects in the future.

Paradoxically, the clinical relevance of modulating lactate is supported by human studies. For instance, administration of sodium lactate was shown to significantly enhance cardiac performance in patients with acute heart failure, suggesting that the timing and context of lactate modulation are critical and that boosting lactate levels can be beneficial in certain clinical scenarios [139]. This finding contrasts with MCT4 inhibition strategies and introduces another important perspective, regulation through lactate-specific influx transporters, such as MCT1.

**Fig. 5:**
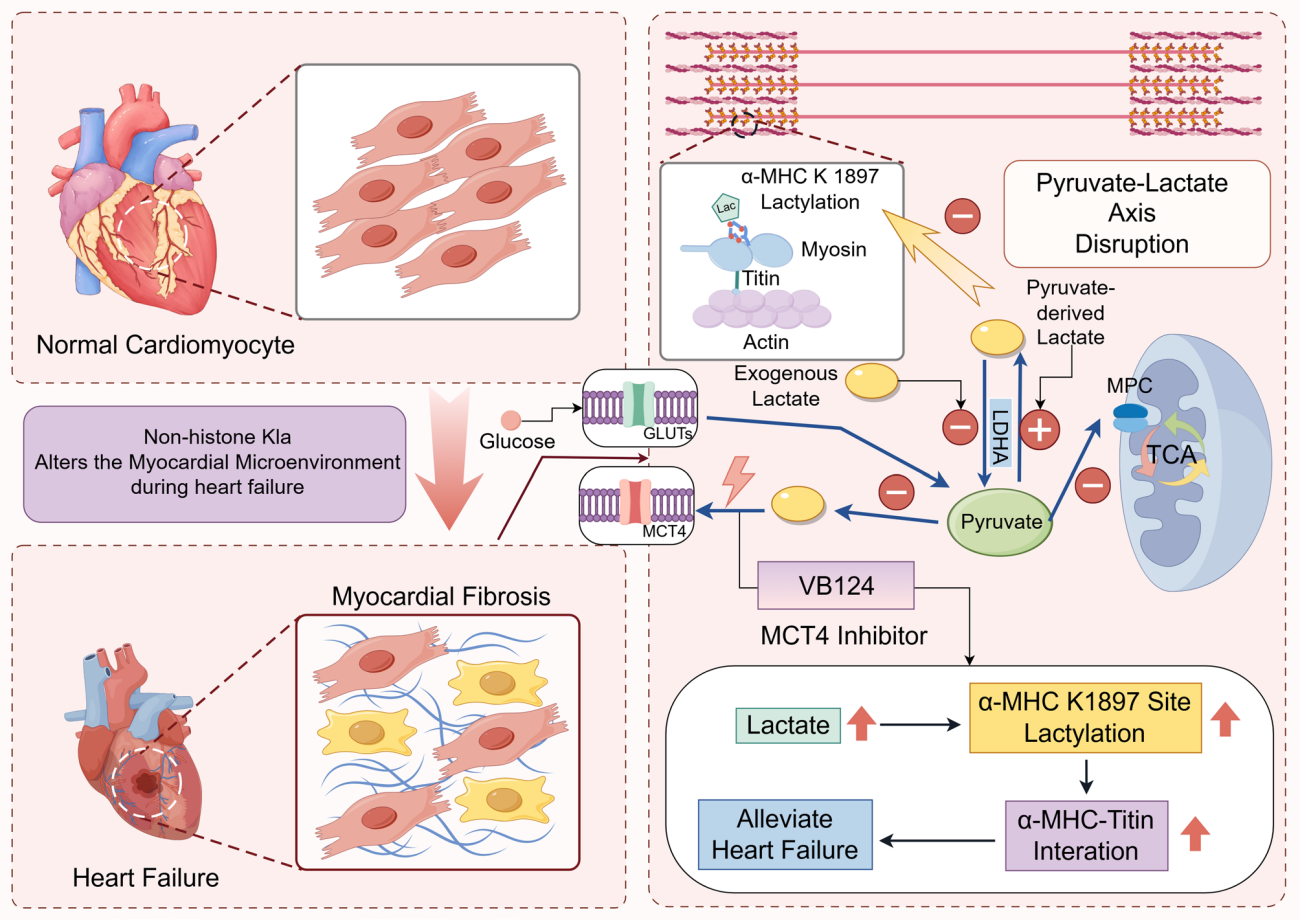
Altered lactate flux and α-MHC lactylation in cardiac stress conditions. In the healthy myocardium, glucose and lactate are metabolically coupled to sustain contractility, with lactate entering cardiomyocytes via MCT1 and being exported through MCT4, maintaining a stable pyruvate–lactate axis. Under pathological stress such as heart failure, this equilibrium is disrupted due to excessive lactate accumulation and overactivation of MCT4, leading to reduced lactylation at lysine 1897 of α-myosin heavy chain (α-MHC). This specific post-translational modification is crucial for maintaining Titin-myosin interactions. Its decline weakens sarcomere function and contributes to fibrotic remodeling. Pharmacological inhibition of MCT4 by VB124 retains intracellular lactate, thereby restoring α-MHC K1897 lactylation and enhancing myocardial contractile function. In addition, multiple non-histone proteins undergo lactylation under cardiac stress, suggesting a broader role for lactate in metabolic and epigenetic regulation of heart function *Abbreviations:* *α*-*MHC* alpha-myosin heavy chain, *Kla* Lysine lactylation, *K1897* lysine 1897, *MCT1*/*4* Monocarboxylate transporter *1*/*4,* *GLUT* Glucose transporter, *LDHA* Lactate dehydrogenase A, *TCA* Tricarboxylic acid cycle, *MPC* Mitochondrial pyruvate carrier

Another revealing perspective is based on the lactate-specific transporter MCT1. Given the increased reliance on lactate for oxidation in impaired cardiomyocytes, these specialized transporters have inevitably received much emphasis. MCT1, as the lactate influx transporter in the sarcolemma, was found to be extensively upregulated in rats with congestive heart failure. Another contributing factor was the formation of a novel cell-intrinsic MCT1 pool in conjunction with specific sarcoplasmic membranes oriented toward the T-tubules [140] (Fig. 5).

Therefore, lactate plays a dual role in cardiovascular disease, serving both as a critical energy substrate for cardiomyocytes and as a modulator of pathological processes such as fibrosis and cardiac dysfunction through lactylation and transporter-mediated mechanisms.

### Obesity

The role of lactate in obesity and insulin resistance is complex and exhibits apparent contradictions, which can be reconciled by considering dose, temporal context (chronic elevation vs. acute supplementation), and specific tissue environments. Unequivocally, recent reports have indicated an alternative metabolic pathway for lactate, in association with lipid anabolism. Lactate enrichment pertains to fatty acid synthesis by serving as a supplementary source of acetyl-CoA, concurrently activating related carboxylases responsible for promoting fatty acid production during shuttle and exchange processes. Seemingly, lactate has been explored as a substrate for lipid storage, and in some cases, it can also promote fatty acid synthesis in rat muscles through glyceroneogenesis-derived glycerol [141]. Contrary to this belief, under an excessive abundance of fatty acids, lactate could responsively exhibit a stimulating effect on fatty acid oxidation through the TCA cycle [19]. The blood tests also demonstrated the negative association between lactate and lipid-oxidation, suggesting that lactate may possibly suppress fatty acid oxidation [142]. A prospective case-control study involving 24 severely obese patients was accompanied by hyperinsulinemia secondary to hyperlactatemia [143].

Nevertheless, the interplay between lactate and insulin resistance in obesity remains multifaceted and often contradictory, as further discussed below. Admittedly, the rise of lactate levels in adipose tissues of obese individuals has long been understood. In the context of chronic hyperlactatemia, lactate is viewed as both a byproduct and a contributor to metabolic dysfunction and systemic inflammation [144].

To reveal lactate’s role in this dynamic process, researchers found that manipulating MCT1-mediated lactate transport led to notable effects. Specifically, MCT1 is abundantly expressed in adipose cells, and selective blockade agents have been shown to cause cell-intrinsic lactate retention, thereby stimulating adipocyte apoptosis, promoting pro-inflammatory cytokine release, and exacerbating insulin resistance in both adipose and surrounding tissues [144]. However, the detailed mechanism responsible for this sequential phenomenon remains vague. Transcriptome analysis revealed a strong association between MCT1 deletion and alterations in inflammation and apoptosis within adipose cells. The inflammatory response appears to be secondary, triggered after lactate-induced apoptosis in adipocytes. These activities could be aggravated by MCT1 inhibition, accompanied by cytokine production, infiltration of inflammatory adipose tissue macrophages (ATMs), and the emergence of insulin insensitivity in nearby tissues [144]. In line with this finding, researchers also discovered that both ATMs and pro-inflammatory cytokines were diminished in the lack of lactate. Mouse models with selective deletion of LDHA exhibited improved glucose tolerance and insulin sensitivity. The specific mechanism may involve adipocyte-derived lactate enhancing ATM polarization into inflammatory subsets by binding competitively to the PHD2 catalytic domain in macrophages, stabilizing HIF-1α expression, and augmenting IL-1β production. These effects were alleviated when lactate was depleted, suggesting that endogenous lactate may enhance inflammation in obesity-related insulin resistance [145].

In stark contrast, exogenous lactate administration at moderate doses in experimental settings reveals a protective, pharmacodynamic potential. This paradigm demonstrates that when introduced as a signal to a stressed system, lactate can activate beneficial pathways. Paradoxically, exogenous lactate supplementation at moderate doses demonstrates protective effects. For instance, when Cai H et al. administered moderate doses of L-lactate (800 mg/kg/day) to mice exposed to a high-fat dietary regimen, they observed suppression of M1 polarization in ATMs. The 12-week lactate administration showed potential in reducing body weight gain and improving insulin sensitivity, indicating therapeutic potential in type II diabetes associated with obesity [146]. Moreover, other studies have reported lactate as a possible dietary supplement acting via the GPR81 receptor. Oral lactate administration was shown to induce GPR81 expression, promoting adipose browning as an anti-obesity mechanism. A possible explanation involves lactate activating the p38-UCP1 axis, supporting the hypothesis that lactate-activated GPR81 may cooperate with β3-adrenergic receptors to enhance thermogenesis [147]. This concentration-dependent effect is epitomized in energy metabolism during exercise, where a shift from white adipose tissue to intramuscular triglycerides as the primary fatty acid source occurs as blood lactate rises from 2 mM to 5 mM, demonstrating lactate’s role as a metabolic rheostat in real-time [148].

**Fig. 6:**
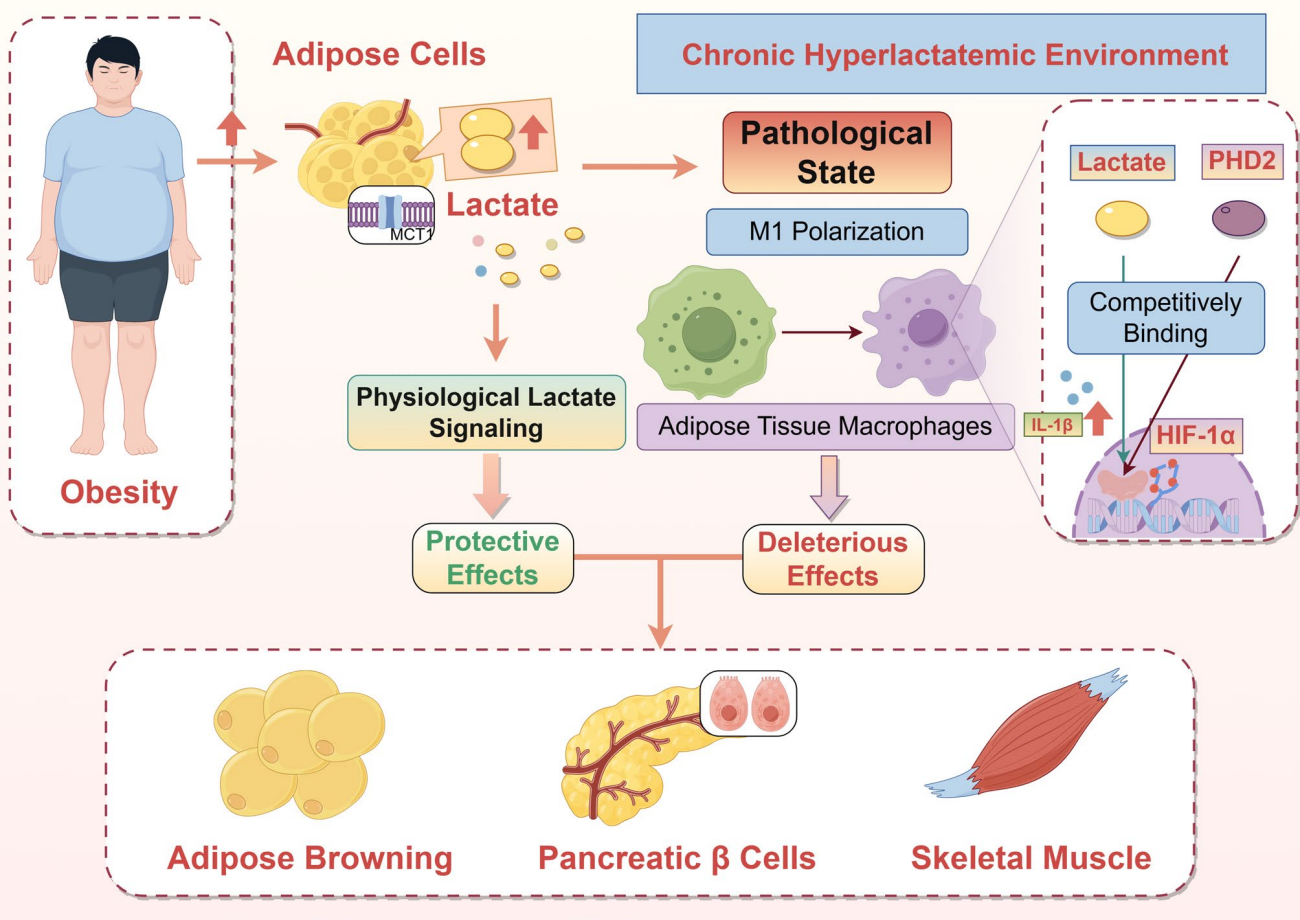
The dual role of lactate in obesity and insulin resistance is governed by dose, temporal, and tissue context. Lactate exerts opposing effects in a context-dependent manner. On one hand, a chronic hyperlactatemic environment, characterized by persistently high lactate levels, promotes a Pathological State. In this setting, lactate within Adipose Tissue Macrophages competitively binds to PHD2, leading to the stabilization of HIF-1α and driving M1 Polarization and pro-inflammatory cytokine production. These events contribute to adipose inflammation and systemic Deleterious Effects, including insulin resistance. On the other hand, Physiological Lactate Signaling, as seen during acute exposure or moderate supplementation, mediates Protective Effects. In this beneficial context, lactate promotes adipose browning and thermogenesis, supports the function of pancreatic β Cells, and enhances metabolic homeostasis in skeletal muscle. Thus, lactate acts not merely as a metabolic byproduct but as a key contextual rheostat fine-tuning metabolic health and disease. *Abbreviations*: *MCT1* Monocarboxylate transporter 1, *ATMs* Adipose tissue macrophages, *PHD2* Prolyl hydroxylase domain-containing protein 2, *HIF*-*1α* Hypoxia-inducible factor 1 alpha, *IL*-*1β* Interleukin-1 beta

In conclusion, the dualistic role of lactate in obesity is not only dependent on its concentration and exposure duration but is also fundamentally governed by the distinct metabolic and signaling pathways it engages in different tissues. As comprehensively reviewed, lactate exhibits “tissue specificity”: in white adipose tissue, it can suppress lipolysis via the GPR81 receptor at high concentrations, yet promote lipolysis and browning via β3-adrenergic receptor and mitochondrial pathways at lower concentrations or under specific conditions such as exercise [149, 150]. Concurrently, in skeletal muscle, lactate can inhibit fat breakdown and promote triglyceride accumulation through the GPR81-cAMP-PKA axis, while simultaneously enhancing mitochondrial biogenesis and oxidative function through metabolic pathways [9, 151]. This intricate, context-dependent network of actions positions lactate not merely as a passive biomarker of metabolic stress, but as an active rheostat that fine-tunes energy substrate utilization across tissues. Future therapeutic strategies must therefore move beyond a simplistic view of lactate, and instead target its specific signaling hubs to harness its beneficial effects while mitigating its pathological contributions (Fig. 6).

### Sepsis

Fairly speaking, lactate serves as a critical but non-specific biomarker in sepsis, reflecting the body’s metabolic stress rather than solely hypoxic conditions. Current sepsis guidelines emphasize that while lactate is a strong prognostic indicator for mortality and the need for resuscitation, its elevation has multiple causes beyond global hypoperfusion [152].

Traditionally, lactate is viewed as a byproduct of anaerobic glycolysis during hypoxia, and hyperlactatemia in sepsis often mirrors circulatory hypoperfusion and the associated decreased pH levels [2]. However, a paradigm shift recognizes that a significant portion of lactate production in sepsis, particularly in hyperdynamic septic shock, stems from adrenergic stimulation-induced aerobic glycolysis (the “Staub-Effect”) in well-oxygenated tissues. This explains the paradoxical observation of significant hyperlactatemia in patients without overt signs of shock, underscoring the complex relationship between lactate and sepsis [152–154]. Therefore, lactate levels, though vital for risk stratification, must be interpreted as a nonspecific marker of metabolic stress within a broader clinical context.

Undeniably, excessive glycolysis exerts significant influences on immune metabolism in severe infection. Transient adaptations to glycolysis can sometimes facilitate immune cell subtype transformations, given their characteristics of adopting metabolic configurations for better energy conservation and rearrangements [155]. The persistent elevation of hyperlactatemia symbolizes the interactive trajectory with inflammation-related cytokines and immune cells. Lipopolysaccharide (LPS)-induced inflammatory responses, as the most representative septic pathway, initiate a premise for immune cell mobilization and are a potent driver of this metabolic reprogramming.

Speaking broadly, monocyte-macrophage systems and mast cells are significant immune subpopulations at septic onset. For instance, considering the crucial functions of mast cells in sepsis, researchers discovered that lactate suppressed the production of LPS-induced cytokines and NF-κB transcriptional activities dependent on MCT1, based on disrupting the constant energy metabolic circulation [156]. Focusing on energy metabolism, sustained ATP production is the decisive force to maintain the intracellular physiological processes. What could be presumed on lactate was its capability to disrupt normal metabolism, thus impairing the corresponding immunity in the sepsis process [157]. More precisely, the activation of LPS is usually accompanied by reinforced glycolysis, which could ensure adequate energy availability for the smooth operation of NF-κB transcriptional activities and cytokine releases; this dynamic process could be suppressed by exogenous lactate. This lactate propensity implied the vicious circle of sepsis, where patients at their terminal stages always exhibited refractory and critical immunosuppressive effects [156, 158]. A similar suppressive phenomenon was observed in bone marrow-derived macrophages (BMDMs) in multiple pathways. Dependent on the GPR81 receptor, researchers found that lactate inhibits activation of the inflammatory cascade via TLR4 signaling, including the NLRP3 inflammasome and NF-κB signaling. Furthermore, the synthesis of IL-1β was subsequently diminished [159].

Beyond that, based on previous assumptions, lactate was surprisingly discovered to exhibit inhibitory effects on wild GPR81(-) macrophage cell lines, which included abrogating LPS-induced IL-6 expression depending on concentration. The non-specificity of this process implied that lactate could exert remarkable influences via other alternative pathways, such as MCT1-dependency, which was assessed to be expressed in macrophages [160]. Apart from this, neutrophils localized in the bone marrow possessed the propensity to produce lactate during the early stages of infectious disease induced by LPS, on the basis of increasing glycolysis, ROS burst primarily derived from NADPH oxidase (NOX) activity, and HIF-1α. The overproduction of lactate would thus be secreted into the circulation through MCT4 and preferentially bind to GPR81 on endothelial cells, to reduce endothelial VE-Cadherin expression and increase vascular permeability through different signaling. This whole dynamic process promoted neutrophil mobilization to a large extent [161]. This host of evidence provides a reasonable conjecture where immune compromises might occur in a lactate-rich circumstance, in conjunction with a group of innate immune cells escalating this route, which are believed to be the first guard to defend the bacterial invasion. For the time being, the studies investigating lactate effects on adaptive immunity in septic pathogenesis are comparatively limited, so more detailed mechanisms require further verification.

**Fig. 7:**
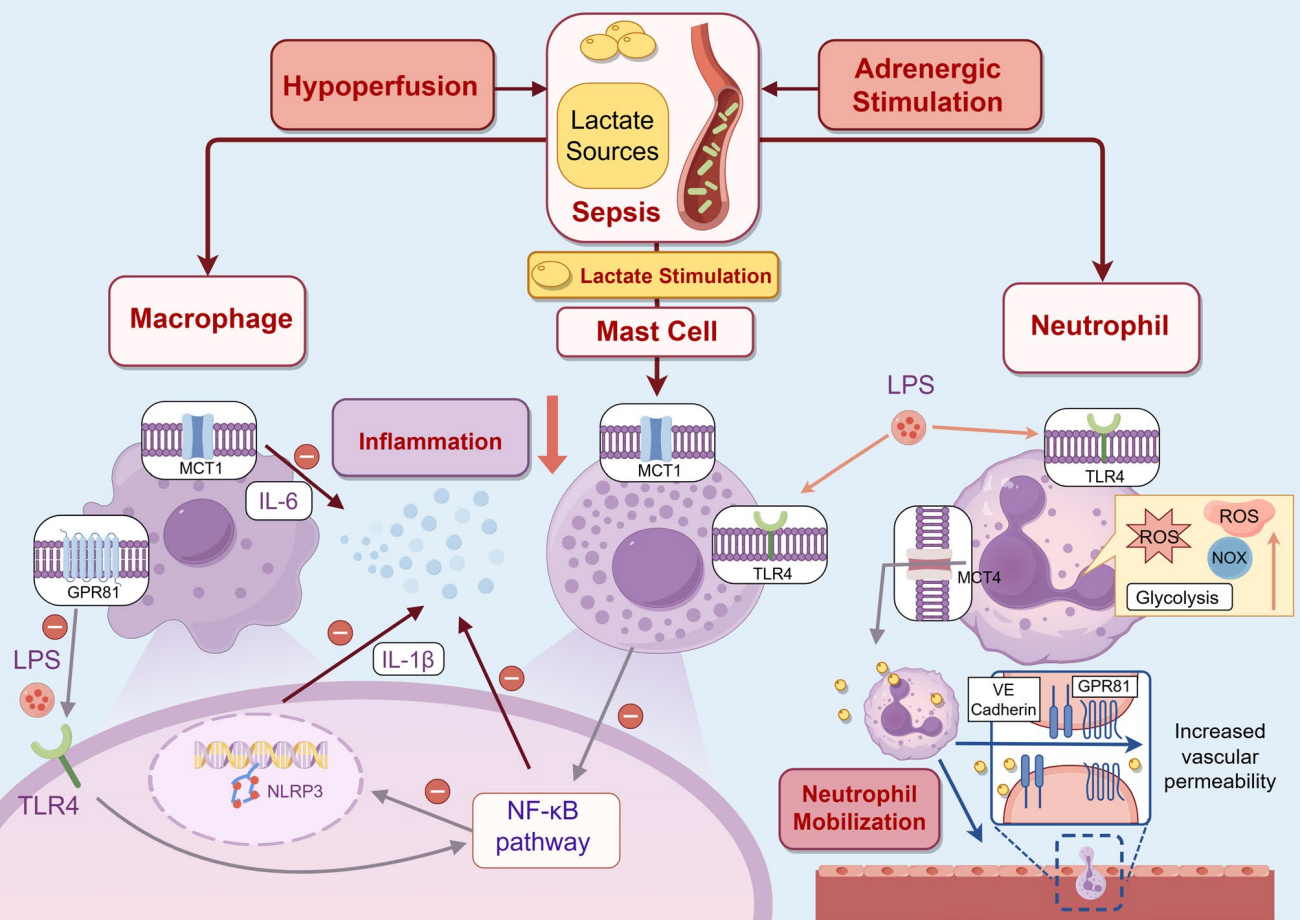
In sepsis, lactate engages in diverse cellular interactions that vary across different immune cell types. Neutrophils, particularly those in the bone marrow, rapidly increase lactate production upon LPS exposure, which may influence endothelial cell function and compromise vascular integrity, potentially mediated via the GPR81 receptor. In macrophages, lactate is sensed through MCT1-mediated transport and GPR81-dependent pathways, modulating cytokine profiles and possibly engaging NF-κB or inflammasome signaling. Mast cells also internalize lactate via MCT1, suppressing LPS-induced cytokine production and NF-κB signaling, thus contributing to the overall anti-inflammatory response. Lactate production in sepsis reflects not only hypoxia but also adrenergic stimulation and aerobic glycolysis. Elevated lactate serves as a prognostic marker in sepsis but is a non-specific indicator of metabolic stress. This illustration underscores lactate’s complex role in immune regulation and vascular permeability during septic conditions. *Abbreviations:* *MCT1*/*4* Monocarboxylate transporter 1/4, *TLR4* Toll-like receptor 4, *IL*-*6* Interleukin-6, *IL*-*1β* Interleukin-1 beta, *GPR81* G protein-coupled receptor 81, *NLRP3* NOD-like receptor family pyrin domain containing 3, *NF*-*κB* Nuclear factor kappa-light-chain-enhancer of activated B cells, *ROS* Reactive oxygen species, *NOX* NADPH oxidase, *LPS* Lipopolysaccharide

Additionally, from the post-transcriptional modification perspective, researchers extracted blood samples from clinical subjects and explored the ubiquitous lactylation in global protein modifications in peripheral blood mononuclear cells both in healthy and shock patients, among which the major subpopulation is H3K18 lactylation. H3K18 lactylation was significantly correlated with the infectious inflammatory cytokines, concurrently prompting ARG1 overexpression in response to inflammation. Therefore, the general H3K18 lactylation status portrayed the landscape associated with the severity and prognosis [84] (Figs. 2 and 7).

### Injury

#### Wound healing

Wound healing is a multifaceted process marked by cellular variation, structural complexity, and functional malleability, during which age-related factors and diabetes are recognized as key contributors to the development of persistent wounds [162]. Wound healing typically progresses through three overlapping phases, including inflammation, proliferation, and tissue remodeling [163]. In general, the post-injury process requires coagulation initiated by interactions between endothelial cells and platelets, whose released mediators trigger an inflammatory response that recruits neutrophils and macrophages. These immune cells release proinflammatory cytokines and growth factors that stimulate stromal cells to mature into myofibroblasts, which promote wound contraction and deposition of extracellular matrix components, enhance epithelial cell proliferation, and induce neovascularization. Eventually, macrophages and extracellular protein-hydrolyzing enzymes remove clots and tissue debris to complete the repair process [164]. Due to the excessive secretion of cytokines during this process, metabolic activities increase rapidly, leading to a hypoxic microenvironment. In this context, lactate becomes essential for a central energy source required to fulfill the metabolic demands of wound healing.

Angiogenesis is a key step in wound healing, as it supplies nutrients essential for cellular proliferation and tissue repair [165]. Porporato et al. reported that lactate can promote angiogenesis and reperfusion in ischemic wounds in mice, indicating that lactate induces healing angiogenesis [166]. Supporting this, elevated levels of VEGF have been associated with enhanced angiogenesis [167]. Given its origin in glycolytic processes, many studies have shown a dynamic link between lactate metabolism and HIF-1α: under low oxygen conditions, HIF-1α attaches to the VEGF promoter, activating its gene expression [168]. Furthermore, HIF-1α upregulation can indirectly enhance VCAM1 expression by inducing C1q binding protein (C1QBP) overexpression and activating the NF-κB signaling pathway [169]. Additionally, lactate has been found to stimulate circulating vasculogenic stem cells within subcutaneous matrix tissue via a mechanism dependent on thioredoxin 1 (Trx1) and HIF-1, forming a positive feedback loop [170].

In addition to promoting angiogenesis, lactate has also been shown to stimulate collagen production in fibroblasts, highlighting the critical role of fibroblasts in extracellular matrix synthesis during wound healing [171]. This suggests that regulating fibroblast metabolism may offer a theoretical basis for enhancing tissue regeneration. Weng et al. demonstrated that platelet-rich plasma (PRP), widely used in regenerative medicine, can stimulate glycolytic enzyme activity in fibroblasts to accelerate wound repair [172]. Moreover, lactate pretreatment was found to shift fibroblast metabolism toward glycolysis, partly through ROS-mediated HIF-1α stabilization.

Beyond fibroblasts, macrophages play a crucial role in post-injury inflammation. Lactate has been demonstrated to trigger macrophage polarization as well as promote tissue regeneration [103]. In later stages of repair, M1 macrophages may be reprogrammed to support the transcriptional activation of genes such as ARG1 [15]. Lactate has also been reported to promote macrophage polarization toward the M2 phenotype, linked to VEGF and ARG1 secretion [107]. These results suggest that lactate facilitates macrophage reprogramming toward a suppressive and tissue-repairing phenotype in response to injury.

**Fig. 8:**
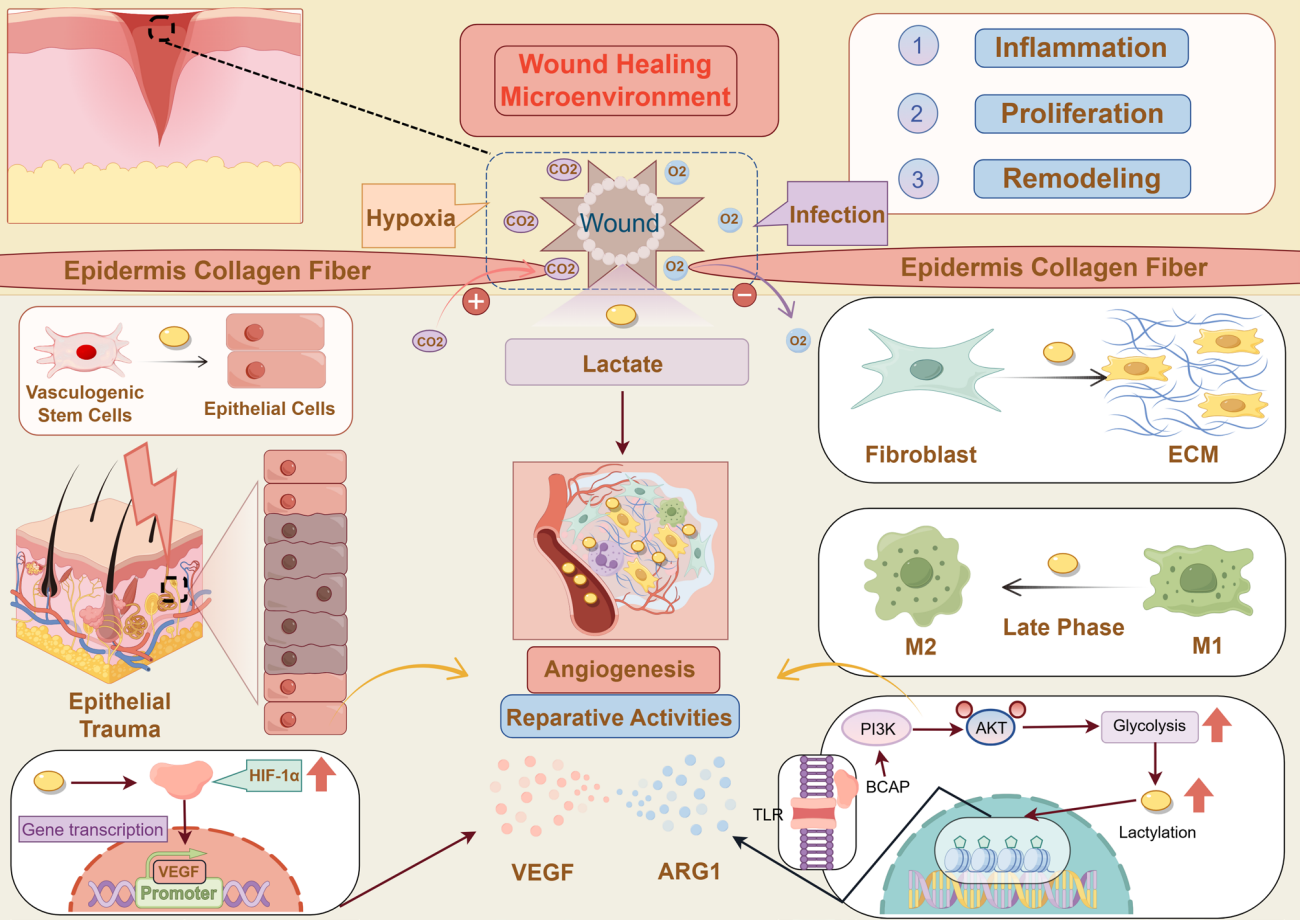
Lactate’s diverse roles in wound healing. Injury induces hypoxia and triggers a cascade of wound healing processes, including inflammation, proliferation, and tissue remodeling. Lactate accumulates in the wound microenvironment, promoting angiogenesis and facilitating tissue repair, in part by upregulating VEGF and ARG1. Additionally, lactate enhances fibroblast function and extracellular matrix synthesis. In the later stages of inflammation, lactate plays a key role in macrophage polarization towards the reparative M2 phenotype. This shift is associated with the activation of PI3K-AKT signaling and increased histone lactylation, which contribute to the resolution of inflammation and the remodeling of tissue. *Abbreviations*: *VEGF* Vascular endothelial growth factor, *ARG1* Arginase 1, *ECM* extracellular matrix, *M1* pro-inflammatory macrophage, *M2* anti-inflammatory macrophage, *PI3K* Phosphoinositide 3-kinase, *AKT* Protein kinase B

From an epigenetic perspective, lactate acts as a “lactate clock” in late-phase M1 macrophages. This transition involves the B cell adaptor for PI3K (BCAP), which, via its N-terminal TIR domain, mediates signal transduction from TLRs to the PI3K-AKT pathway, promoting aerobic glycolysis and lactate production. Loss of BCAP impairs this metabolic shift, resulting in reduced histone Kla and diminished expression of reparative genes, accompanied by prolonged inflammation due to reactivation of FOXO1 and GSK3β. In BCAP-deficient mice, these defects could be rescued by exogenous lactate, confirming that histone Kla and reparative programs can be restored through lactate supplementation, with increased Arg1 and Klf4 expression serving as key markers of this transition [173]. Thus, lactate appears to act as a driving force for macrophage immunomodulation. An imbalance in cellular immune status influenced by lactate may affect the severity of inflammatory diseases, providing new insight into potential therapeutic approaches for chronic inflammatory wounds [174] (Fig. 8).

#### Anti-ROS

Reactive Oxygen Species (ROS), a key hallmark of neurodegeneration, are dependent on mitochondria for a vast majority of production, and aging mitochondria can produce substantial quantities of ROS due to dysfunction of the respiratory chain, which has been witnessed particularly in Alzheimer’s disease (AD) and Parkinson’s disease (PD) [175, 176]. AD is characterized by amyloid beta plaques with significantly increased lactate levels owing to glucose hypometabolic activities [177]. Surprisingly, the production of lactate is presumably inducing a mild release of ROS inside the mitochondria while concurrently triggering defensive mechanisms responsible for maintaining pro-survival pathways, including PI3K-AKT pathway activation and Endoplasmic Reticulum (ER) protein processing. This finding sheds light on the lactate clinical utility to treat aging-related disorders as a response-activating agent [178]. However, the lactate-induced response mechanism based on a mild ROS burst seems to be restricted in scope. Researchers have also observed this phenomenon in Schwann cells. In Schwann cells, Rheb gene knockdown was utilized to specifically inhibit PDH activity, which shifted the metabolic pattern towards a lactate-predominant oxidized route, thereby transporting increased lactate into peripheral axons. Parallel to the above findings, a slight lactate increase could fuel ATP production in the mitochondria and trigger ROS-dependent pro-survival signaling. However, prolonged exposure to ROS could exacerbate axon damage, reflecting the complexity of lactate metabolism in neuronal support [179]. In pulmonary fibrosis, lactate accumulation has already been observed with the tendency to promote disease progression. Under hypoxic conditions, lactate could enter fibrogenic mesenchymal progenitor cells through the GPR81 receptor to enhance their self-renewal, intensified by HIF-1α augmenting GPR81 expression [180]. In light of this assumption, Sun et al. continued exploring the corresponding mechanism. They found that lactate levels increased concurrently with rising ROS levels, secondary to lactate-induced alterations in mitochondrial morphology and function through DRP1 and ERK modulation [181].

#### Ischemia-reperfusion injury

Lactate has long been widely recognized as being involved in metabolic remodeling within the central nervous system. Across varied intrinsic energy absorption among cell types, aberrant metabolism has been shown to predispose to the pathophysiological progression of neuronal disorders [177]. Admittedly, lactate has been found to participate in various energy activities typical of glycolytic metabolism, despite excessive oxygen consumption, due to the heterogeneity of neurocytes with distinct metabolic features within the central nervous system, such as astrocytes [182]. Magistretti PJ et al. proposed a model in which lactate is transferred as a regulatory molecule rather than simply being a metabolic end-product. The lactate transported from astrocytes into neurons plays a crucial role in activating signaling cascades via MCT2 and HCAR1 (GPR81) receptors, involving plasticity-associated genes [183, 184]. Therefore, this intricate trajectory contextualizes astrocyte-neuron interactions, wherein accumulated extracellular lactate can serve as an energy source for neurons. Moreover, Suzuki A et al. proposed that MCT1/4 blockers on astrocytes could potentially increase the incidence of amnesia, thereby impairing LTP functions mediated by lactate consumption, implying the role of lactate in synaptic plasticity [47]. Furthermore, conclusive evidence was demonstrated by the observation that lactate in neurons has the potential to stimulate the expression of synaptic plasticity-associated genes such as Arc in anN-methyl-D-aspartate (NMDA) receptor-dependent manner. Lactate-induced NMDA receptor activation thus triggers calcium influx into the neuronal cytoplasm. The calcium influx then initiated a signaling cascade leading to ERK1/2 activation, which belongs to one of the MAPK subfamilies, thereby promoting gene expression associated with synaptic remodeling [33]. Moreover, increased NADH levels resulting from astrocyte-derived lactate utilization in gluconeogenesis also act as a driving force to positively stimulate NMDA receptor activity. According to Bajaffer A et al., other upregulated genes significantly related to synaptic plasticity include EGR1 and BDNF [185].

It is worthwhile to explore more specific mechanisms involved in lactate-mediated signaling and physiological maintenance. More specifically, HCAR1 (GPR81) is located at the interface between pial fibroblast-like cells and pericyte-like cells, promoting cerebral vascular endothelial growth and angiogenesis in a VEGFA-dependent manner, independently of MCTs and as an alternative pathway induced by intense exercise [186]. Consequently, some researchers have suggested promising roles for lactate in exerting protective effects, including promoting cell survival during reperfusion injury, with evidence supporting its potential to alleviate such damage. Rather than merely serving as a metabolic end-product, lactate can play a pivotal role in preventing ischemia-reperfusion injury by acting as an efficient energy source. This phenomenon is demonstrated by observations showing that lactate-induced ischemic preconditioning exerts neuroprotective effects on human model cells in vitro [187]. This finding supports the potential clinical utility of lactate in ischemic stroke. However, it is crucial to add nuance to this perspective. The beneficial role of lactate as a metabolic fuel, primarily observed in controlled experimental models, stands in contrast to clinical data from stroke patients. Clinical studies consistently associate elevated systemic lactate levels with poor prognosis. For instance, elevated lactate concentrations upon ICU admission are independently linked to higher mortality in ischemic stroke, reflecting its dual role as both a metabolic substrate and a prognostic biomarker of disease severity [188]. Thus, while lactate may support neuronal survival in controlled models, its systemic accumulation in clinical contexts often signals worse outcomes. Furthermore, a recent retrospective analysis identified the Lactate Dehydrogenase to Albumin Ratio (LAR) as an independent predictor of 3-month post-thrombolysis outcomes in ischemic stroke patients, supporting the prognostic relevance of lactate-related biomarkers in cerebrovascular events [189]. This dichotomy underscores that while locally administered lactate may be neuroprotective, elevated systemic lactate serves as a robust biomarker of global metabolic derangement and disease severity, heralding a worse clinical outcome.

Additionally, bedside microdialysis monitoring in patients with subarachnoid hemorrhage revealed that dynamic elevations of extracellular lactate and glycerol closely correlate with delayed cerebral ischemia, highlighting lactate’s utility as a sensitive indicator of acute metabolic distress in neurological injury [190]. As is well known, after encountering blood and nutrients obstructions for a time limit, normal cells will sustain irreversible damage that exacerbates cellular impairment even when reacquiring blood reperfusion, including uncontrollable pro-inflammatory cytokine storms, ROS activation, and lipid peroxidation [191]. The extent of organ damage depends on the reliability degree of absorbing oxygen from the red blood cells, so the specific cells surrounded with rapid circulatory blood, whose tissues belong to the brain or heart, are the most influenced organs by the ischemia reperfusion damage and the focus of research topic.

**Fig. 9:**
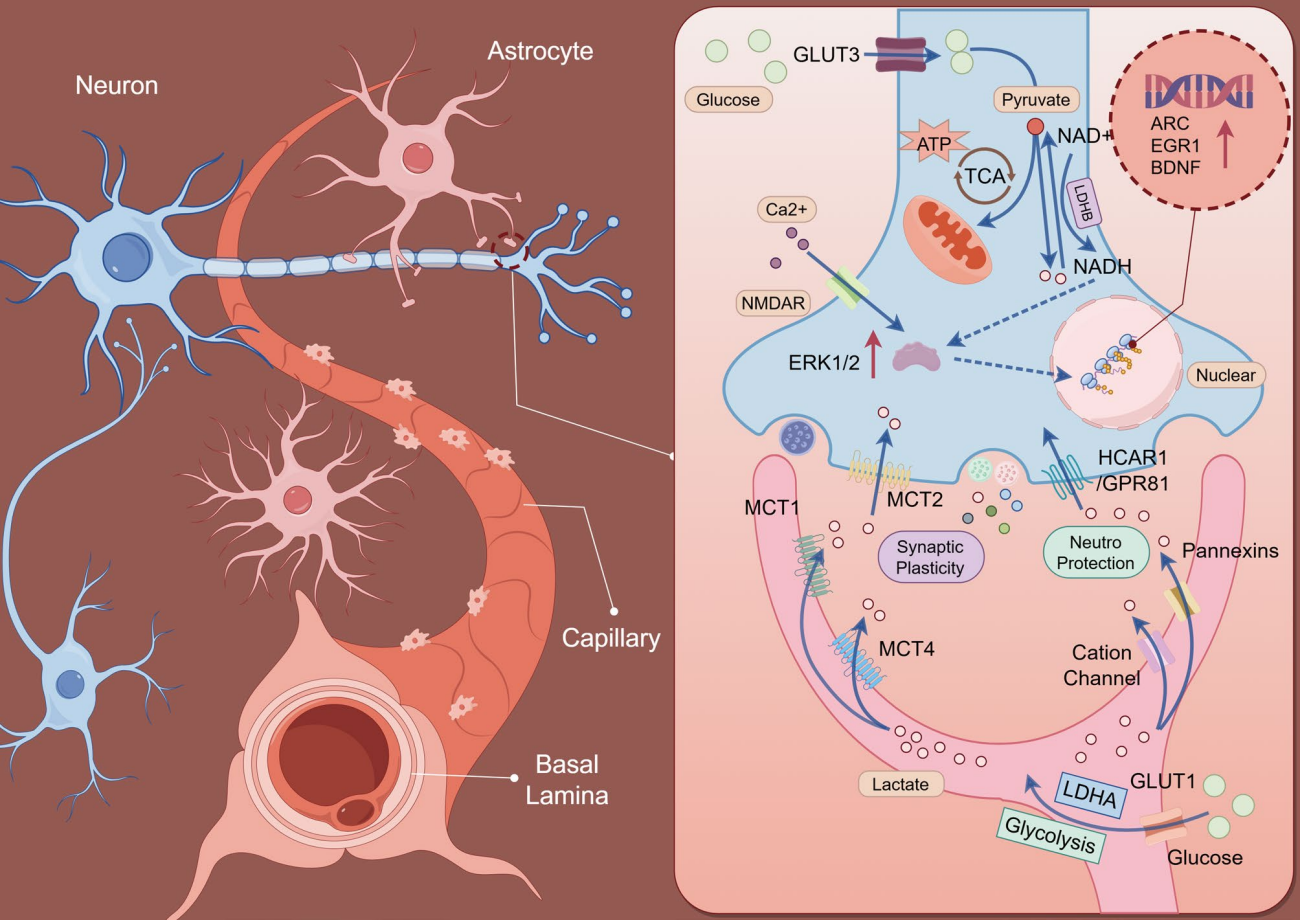
Lactate-induced synaptic plasticity and neuroprotection via the astrocyte-neuron lactate shuttle (ANLS). Astrocytes take up glucose through GLUT1 and produce lactate via glycolysis, catalyzed by LDHA. The lactate is then released into the extracellular space through MCT1, MCT4, and Pannexin channels. Neurons absorb lactate via MCT2 and convert it into pyruvate, which enters the mitochondrial TCA cycle to generate ATP and NADH. NADH, in turn, activates NMDARs, leading to calcium influx and subsequent ERK1/2 signaling activation, which promotes the expression of synaptic plasticity-related genes such as ARC, EGR1, and BDNF. Additionally, lactate triggers neuroprotective pathways through HCAR1 (GPR81) receptors. This illustration highlights lactate’s essential role in energy production, signaling, and gene regulation. *Abbreviations*: *GLUT1* Glucose transporter 1, *LDHA* Lactate dehydrogenase A, *MCT1*/*2*/*4* Monocarboxylate transporter 1/2/4, *TCA* Tricarboxylic acid cycle, *NADH* Nicotinamide adenine dinucleotide (reduced form), *NMDAR* N-Methyl-D-aspartate receptor, *ERK1*/*2* Extracellular signal-regulated kinase 1/2, *HCAR1 *(GPR81) Hydroxycarboxylic acid receptor 1

Beyond metabolic dysfunction, structural compromise of the neurovascular unit further contributes to ischemic injury. The basal lamina of capillaries, a structural barrier closely associated with astrocytic endfeet, also plays a key role in maintaining blood-brain interface integrity during ischemia-reperfusion [192]. Disruption of this anatomical interface impairs not only barrier integrity but also glial-neuronal metabolic coupling. Such coupling is exemplified by the ANLS hypothesis, where astrocyte-secreted lactate acts as an energy supplement for neurons during nutrient deprivation (See Fig. 9 for schematic overview). Generally, astrocyte-derived lactate is mainly released through monocarboxylate transporters; however, emerging evidence indicates that alternative release pathways may also exist [193]. For instance, recent studies in mouse models have revealed that astrocytes maintain an intracellular lactate reservoir that can be rapidly mobilized in response to neuronal cues such as elevated extracellular K^+^ or membrane depolarization. Specifically, a putative lactate-permeable ion channel, activated by astrocytic depolarization and exhibiting approximately 37 pS conductance, has been identified in vitro; this channel appears to be positively modulated by intracellular lactate levels, forming a possible feedback loop for rapid lactate efflux [13]. Moreover, connexin hemichannels, which are traditionally associated with ATP and metabolite release, have been implicated in lactate transport during hypoxia or intense synaptic activity. Although pannexin channels are primarily known for mediating ATP release, some experimental evidence suggests they may also participate in lactate flux under specific non-homeostatic conditions, though this remains speculative and requires further validation [14]. These findings from animal models suggest that astrocytic lactate release may involve multiple pathways beyond monocarboxylate transporters, allowing adaptive responses to neuronal energy demands and pathological stress.

Crucially, the functional significance of this lactate shuttle has been strongly supported by interventional studies. Specifically, downregulation of the neuronal lactate transporter MCT2 or the astroglial lactate transporter MCT4 in the rat barrel cortex abolished the lactate rise in response to sensory stimulation. Under the same conditions, the hemodynamic response measured by blood oxygen level-dependent (BOLD) functional MRI, a primary technique for visualizing brain activity by detecting local changes in blood oxygenation and flow that correlate with neural activation, was completely prevented in all MCT2-downregulated rats. Intriguingly, approximately half of the MCT4-downregulated animals also lost their BOLD response, and this deficit could be rescued by peripheral lactate infusion, a rescue not possible in MCT2-downregulated rats. When assessed behaviorally, MCT2-downregulated animals were impaired in a textured object recognition task, while a similar proportion of MCT4-downregulated animals (about half) showed an identical deficit, mirroring the bifurcation observed in the neurovascular responses. These data collectively demonstrate that ANLS is indispensable for both the neurometabolic and neurovascular coupling processes that underpin functional brain imaging signals and is necessary to sustain behavior driven by cortical activation [194]. It is important to note that beyond its role as an energy substrate, lactate also functions as a signaling neuromodulator. Activation of the lactate receptor HCAR1 has been consistently shown to decrease the activity and excitability of cortical neurons via both pre and postsynaptic mechanisms. This modulatory role, which is distinct from its metabolic function, adds another layer of complexity to the interpretation of neurovascular coupling and may contribute to the BOLD signal [195]. Therefore, the observed effects of lactate shuttling on brain activation and behavior likely involve the integrated contribution of both its energy-delivering and its signal-transducing functions.

Additionally, within the peripheral nervous system, Schwann cells play a crucial role in this process, undergoing metabolic reprogramming via upregulated glycolysis to protect injured axons [196]. Furthermore, the augmented mammalian target of rapamycin complex 1 (mTORC1) pathway mediates the glycolytic shift and sustained energy support, in conjunction with downstream signals of both HIF-1α and c-Myc, thus responsively protecting injured axons [197]. Most importantly, post-injury axons undergo a dramatic upregulation of MCTs, suggesting enhanced lactate influx to meet increased energy demands as a protective mechanism [197]. However, other studies have revealed that lactate-induced neuroprotective effects may only occur in the presence of specific anesthetics, due to its potential to shift the preferential metabolic route when ATP stores are depleted [198]. Similar studies have also reported lactate’s protective effects in conjunction with isoflurane anesthesia administration [199]. Thus, whether lactate can be used as a protective agent in ischemia-reperfusion remains to be conclusively determined.

Additionally, lactate has been investigated as a potential post-treatment for myocardial cells after infarction [200]. A recent study identified a significant intestinal metabolite, indole-3-lactate, as a protective agent that alleviates intestinal ischemia-reperfusion in mouse models by regulating YAP and Nrf2 [201]. More specifically, compared with earlier assumptions, researchers focusing on lactate-promoted protective effects after organ ischemia-reperfusion are scarce. Possible reasons may include the difficulty in distinguishing the effects of endogenous versus exogenous lactate, as well as challenges in translating findings into broad clinical applications.

### Lactate and tumor

#### Tumor adaptation

The glycolysis-based metabolic preferences of tumor cells (“Warburg Effect”) render lactate closely connected with tumor cell metabolism. Lactate also profoundly alters the TME through a series of sophisticated mechanisms. TAp73 enhances lactate metabolism through upregulation of PFKL (phosphofructokinase-1, liver type) and serves as a crucial upstream regulator in the mechanisms of lactate accumulation and lactate-associated tumor adaptation, making it an important molecular node linking tumor metabolic reprogramming to lactate accumulation [202].

First, the frontline of controlling lactate entry into the tumor lies in specific transporters, which predominate in lactate import and export. As explained above, both MCT1 and MCT4 play central roles in tumor metabolic activities. MCT1 exhibits relatively high affinity for lactate and primarily mediates its uptake under most tumor microenvironmental conditions. However, depending on the transmembrane H^+^/lactate gradients, MCT1 can also facilitate lactate efflux, particularly in cells with fluctuating metabolic demands. This bidirectional transport is not constant, but governed by dynamic proton-coupled concentration gradients [8, 43]. MCT4, which is abundantly found in cells with high glycolytic activity, primarily exports lactate due to its low affinity for the substrate. Then, the redundant lactate is transported to the extracellular matrix, assisting tumor cells in maintaining intracellular pH homeostasis [53].

Importantly, MCT1 has been identified as a functional and prognostic marker in multiple cancers. In osteosarcoma, MCT1 expression was confirmed across several cell lines and primary tumors. Inhibition of MCT1 significantly delayed tumor growth both in vitro and in vivo, including in orthotopic models. Notably, MCT1 blockade also enhanced the sensitivity of tumor cells to chemotherapeutics like adriamycin and suppressed their metastatic potential. Mechanistically, these antitumor effects were associated with suppression of the NF-κB pathway, and high MCT1 expression correlated with poorer overall survival in patients [203]. In addition to membrane transport, lactate also functions as a signaling molecule via its receptor GPR81 (HCAR1). Initially identified in adipose and muscle tissue, GPR81 is now recognized to be overexpressed in solid tumors, where it serves as a metabolic sensor for lactate. Silencing of GPR81 dramatically reduced tumor cell survival in lactate-supplemented, low-glucose conditions, suggesting its essential role in lactate utilization under nutrient stress. In vivo, GPR81 expression correlated with increased tumor growth and metastasis in pancreatic cancer models. Furthermore, GPR81 modulates the expression of lactate-handling genes such as MCT1/4, indicating its feedback control over both lactate signaling and transport [204]. These findings highlight the multifaceted roles of lactate in tumor adaptation, not only as a metabolic substrate but also as a signal integrator that coordinates energy metabolism and transport systems within the TME.

#### Tumor immunosuppression

In the TME, metabolic reprogramming not only provides energy and biosynthetic substrates to support the accelerated growth of tumor cells, but also significantly changes the local immune ecology. A prominent immunosuppressive characteristic is the accumulation of lactate due to elevated glycolysis, creating an acidic, lactate-enriched microenvironment that inhibits the proliferation, cytokine release, and cytotoxic activities of T cells and NK cells. This environment also promotes macrophage polarization towards a pro-tumor M2-like phenotype, thereby reducing anti-cancer immune responses [205–207]. Additionally, lactate contributes to metastatic regulation through epigenetic-related signaling. As previously discussed, lactate-driven M2 macrophage polarization also constitutes a key mechanism contributing to the immunosuppressive TME that facilitates tumor spread in breast cancer [95].

Beyond immunometabolic interference, lactate mediates immune suppression through epigenetic and receptor-mediated signaling mechanisms. For example, in colorectal cancer, elevated lactate levels induce H3K18 lactylation and direct lactylation of methyltransferase-like 3 (METTL3) at RNA-binding zinc-finger domains, enhancing its ability to catalyze m⁶A modification of JAK1 mRNA. This promotes YTHDF1-dependent translation, JAK1 upregulation, and downstream STAT3 activation, thereby reinforcing the immunosuppressive phenotype of tumor-infiltrating myeloid cells (TIMs) and promoting immune evasion [208]. These findings highlight the emerging role of non-histone lactylation in post-transcriptional gene regulation [96].

**Fig. 10:**
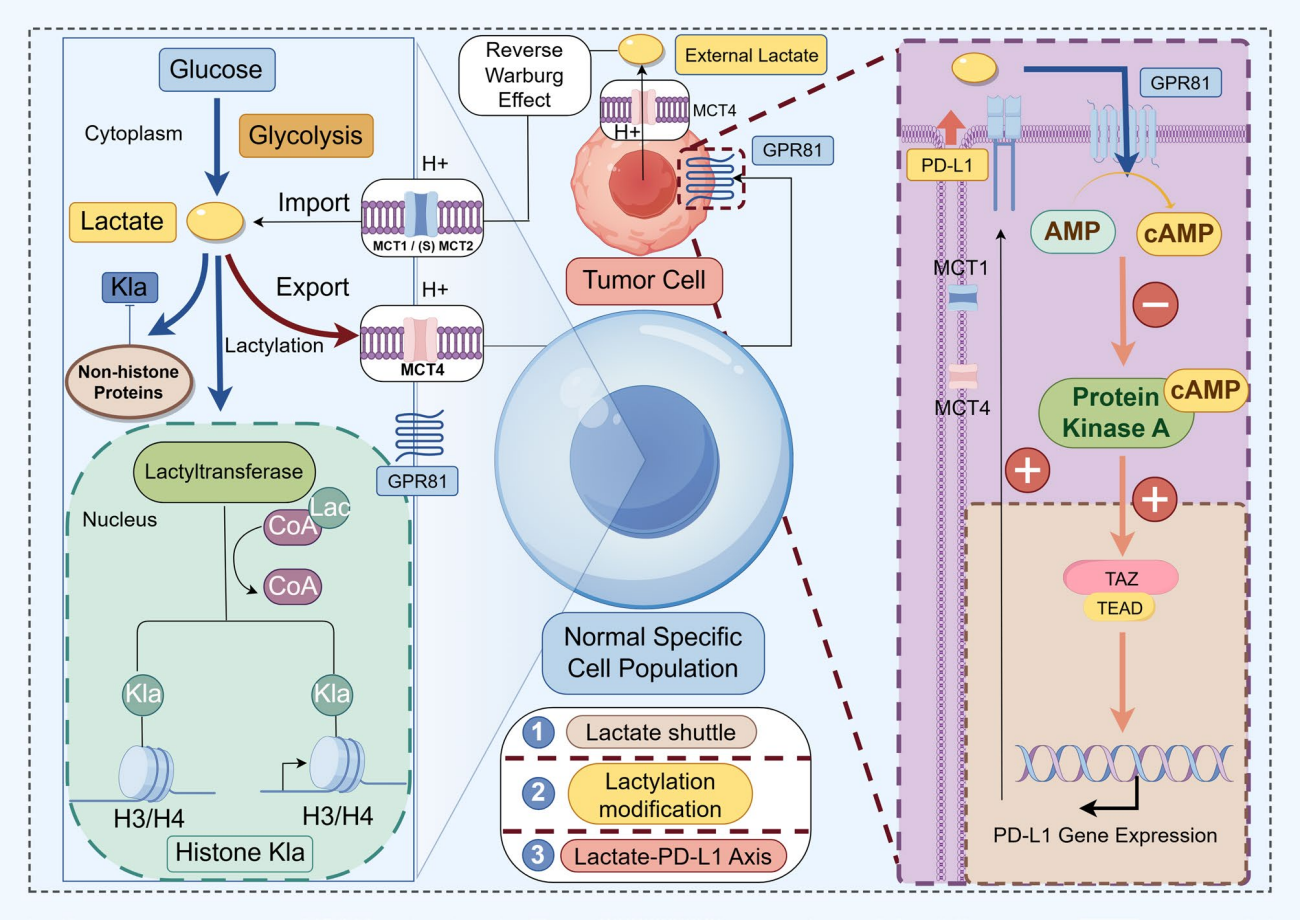
Integrated roles of lactate in metabolism, epigenetics, and immune suppression. (1) Lactate shuttle: Lactate is exported from glycolytic tumor or other hypoxic cells via MCT4, while oxidative neighboring cells import it via MCT1 for mitochondrial respiration. This bidirectional lactate flow forms a “Lactate shuttle”, enabling metabolic coupling across hypoxic and normoxic cells. In specific contexts, such as white adipose tissue, lactate also signals through GPR81 receptors; in rheumatoid arthritis, lactate uptake by CD4⁺ T cells is mediated via SMCT2, while in the central nervous system, astrocyte-secreted lactate flows into neurons through MCT2 as a fuel source for promoting neuronal plasticity. The shuttle maintains energy homeostasis and contributes to signaling transduction (2) Lactylation modification (epigenetic regulation): Intracellular lactate is converted to lactyl-CoA, serving as a donor for lysine lactylation (Kla) catalyzed by a putative lactyltransferase (currently unidentified, though p300 has been proposed as a potential candidate). Histone lactylation (e.g., H3K18la, H3K9la) promotes chromatin accessibility and gene transcription. Non-histone lactylation affects protein stability and signaling cascades. (3) Lactate-PD-L1 axis (immune escape): Extracellular lactate activates the GPR81 receptor, suppressing adenylate cyclase and reducing intracellular cAMP. This downregulation deactivates PKA, enabling nuclear translocation of TAZ. TAZ binds TEAD to promote PD-L1 transcription. Elevated PD-L1 expression facilitates T cell suppression, forming a lactate-GPR81-TAZ-PD-L1 signaling loop linking metabolism with immune evasion. *Abbreviations*: *MCT* Monocarboxylate transporter, *Kla* Lysine lactylation, *PD*-*L1* Programmed death-ligand 1, *MCT1 */*MCT4* Monocarboxylate Transporter 1/4, *SMCT2* Sodium-coupled Monocarboxylate Transporter 2, *GPR81 *(*HCAR1*) G Protein-Coupled Receptor 81 (Hydroxycarboxylic Acid Receptor 1), *Kla* Lysine Lactylation, *LDH* Lactate Dehydrogenase, *PD*-*L1* Programmed Death Ligand 1, *AMP */*cAMP* Adenosine Monophosphate/Cyclic Adenosine Monophosphate, *PKA* Protein Kinase A, *CoA* Coenzyme A, *Lac* lactyl-CoA, *H3 */*H4* Histone 3/Histone 4, *HDAC* Histone Deacetylase

In parallel, lactate signals through its G-protein-coupled receptor GPR81 (HCAR1) to promote immune escape. In lung cancer cells, lactate activates GPR81, which reduces intracellular cAMP and inhibits PKA activity, leading to activation of the transcriptional coactivator TAZ. TAZ then interacts with TEAD transcription factors to induce PD-L1 expression, suppressing T cell activity. This lactate-GPR81-LDHA-TAZ-PD-L1 axis constitutes a key immunosuppressive circuit within the TME that mechanistically links metabolic reprogramming to immune evasion [209]. To visually summarize these mechanisms, Fig. 10 provides an integrated schematic of how lactate mediates metabolic coupling, epigenetic remodeling, and immune evasion in the TME. It highlights intercellular lactate shuttling, intracellular lactyl-CoA-driven protein lactylation, and the lactate-GPR81-TAZ-PD-L1 signaling axis that facilitates immunosuppression.

Lactate-induced extracellular acidosis further contributes to immunosuppression by impairing tumor-infiltrating lymphocyte (TIL) function and promoting tumor invasiveness [210]. Specifically, lactateosis inhibits cytokine production (such as IFN-γ) and partially blocks lysosomal granule efflux in cytotoxic T lymphocytes (CTLs). Mechanistically, it selectively suppresses JNK, c-Jun, and p38 phosphorylation while sparing MEK1 and ERK signaling, thus compromising CTL effector functions. Importantly, this immunosuppression is rapid and reversible, and buffering extracellular acidity can restore CTL function even in the presence of high lactate levels [211]. In light of these immunosuppressive effects, regulating lactate export has emerged as a potential therapeutic strategy. In light of these immunosuppressive effects, regulating lactate export has emerged as a potential therapeutic strategy. To handle lactate accumulation and maintain pH homeostasis, tumor cells upregulate lactate transporters such as MCT1 and MCT4, facilitating lactate efflux to avoid intracellular acidification [212]. Targeting these transporters with MCT inhibitors has been proposed as a therapeutic strategy to disrupt lactate recycling and tumor metabolism [67]. However, mathematical modeling suggests that MCT inhibition alone may not significantly decrease lactate production due to autoregulatory feedback on flux control [213]. Additionally, MCT1/4 contribute to immune dysfunction, angiogenesis, and chemoresistance through interactions with stromal components in the TME [38].

Interestingly, lactate may also play an immunostimulatory role under specific conditions. Kaymak et al. reported that moderate lactate concentrations can upregulate TCF-1 expression in CD8^+^ T cells, enhancing stem-like properties and memory potential, thereby prolonging antitumor responses [214]. These findings suggest that the immunoregulatory effects of lactate are highly context-dependent, influenced by concentration, extracellular pH, and microenvironmental cues. Therefore, it is crucial to distinguish between lactate itself and lactate-induced acidosis when assessing its role in immune modulation.

#### Tumor angiogenesis and metastasis

Lactate is a key byproduct of glycolytic activity in tumors and serves a significant function in regulating signaling within the TME. Increasing evidence indicates that lactate is essential for promoting angiogenesis in cancers, thereby driving tumor progression [215].

In terms of angiogenesis, lactate can enter vascular endothelial cells via MCT1, leading to IκBα phosphorylation and subsequent degradation, thereby activating the NF-κB pathway and upregulating IL-8 transcription, a key mediator of angiogenesis. This signaling axis enhances the motility of endothelial cells and lumen formation, and is an important driver of tumor neovascularization. Studies have also found that this process relies on the interplay between ROS and PHD signaling. More critically, in colorectal and breast cancer xenograft models, tumor cells activate this IL-8-dependent pathway by releasing lactate via MCT4, which promotes neovascularization and tumor growth. This suggests that the lactate/NF-κB/IL-8 pathway serves as a critical hub connecting tumor metabolic activity and angiogenic processes. Additionally, lactate enters vascular endothelial cells via MCT1, which can activate the HIF-1 pathway and upregulate the expression of VEGFR2 and bFGF under normoxic conditions, promoting angiogenesis. Blocking this process significantly inhibited tumor-associated neovascularization. This pathway demonstrates lactate’s function as an active signaling agent in angiogenesis and underscores the therapeutic potential of targeting MCT1 for both metabolic inhibition and suppression of neovascularization [216].

**Fig. 11:**
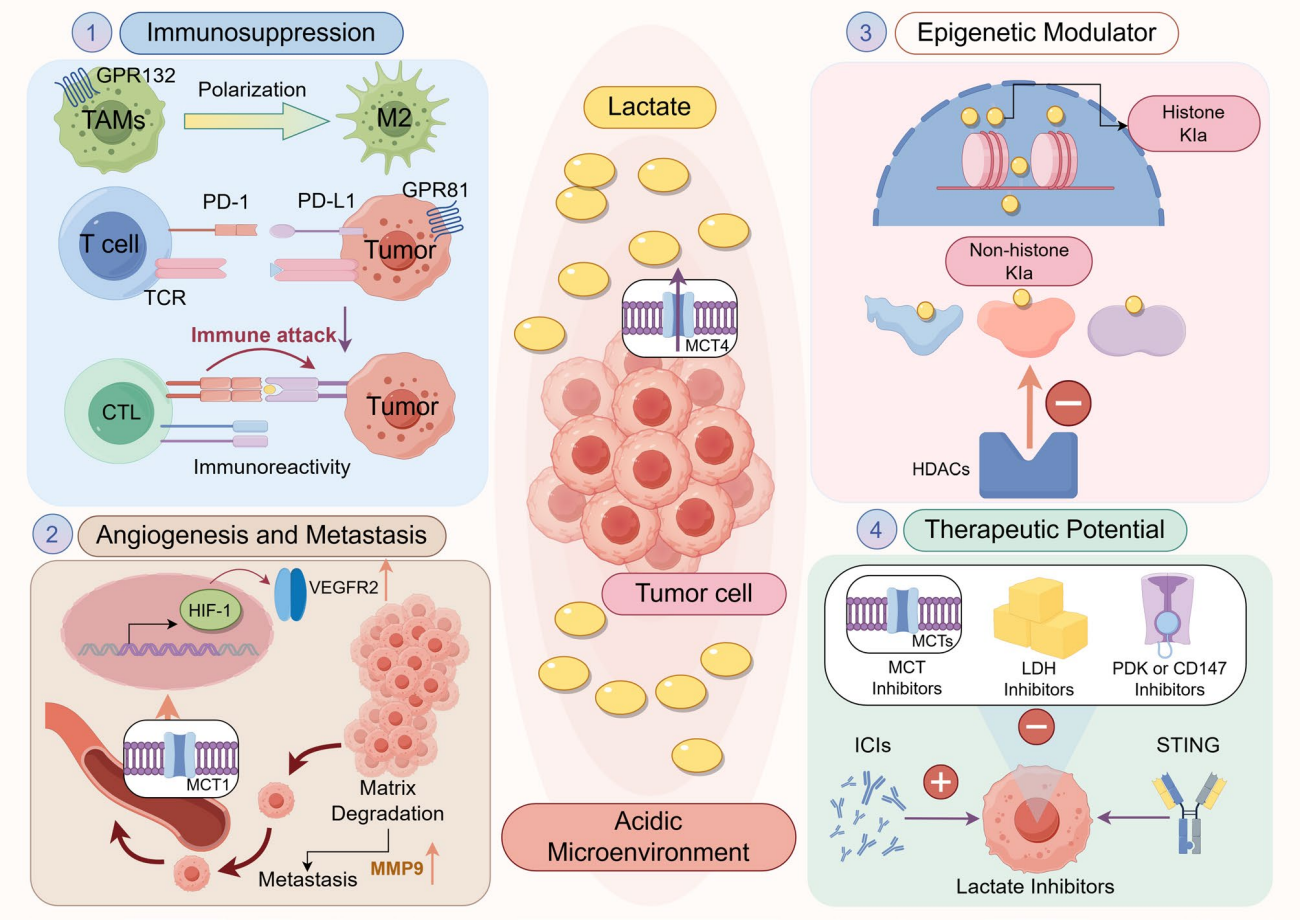
Lactate’s multiple roles in tumorigenesis and progression Tumor cells produce excess lactate due to enhanced glycolysis, which is transported to the extracellular space and secreted into the extracellular matrix, contributing to the establishment of an acidic microenvironment. (1) Immunosuppression: Lactate inhibits anti-tumor immunity by impairing T cell and NK cell functions and promoting the polarization of macrophages into the M2-like phenotype. (2) Lactate-driven angiogenesis and metastasis: Lactate is taken up by vascular endothelial cells via MCT1 and activates the HIF-1 pathway under normoxic conditions, which upregulates VEGFR2 expression, thereby promoting angiogenesis. Simultaneously, lactate-mediated extracellular acidification enhances MMP9 activity, which weakens cell-cell adhesion and supports metastasis. (3) Regulation of epigenetic mechanisms: Lactate regulates epigenetic processes by inducing lysine lactylation on histones, particularly H3 and H4, altering chromatin structure and gene expression. Lactylation also occurs on various non-histone proteins, impacting cytoplasmic signaling and cellular functions, collectively reshaping the tumor immune microenvironment. Histone deacetylases (HDACs) are responsible for removing lactylation marks, presenting potential therapeutic targets for epigenetic intervention. (4) Therapeutic targeting of lactate metabolism: Inhibiting lactate metabolic pathways by targeting key proteins such as LDH, MCTs, and CD147, in combination with immune checkpoint inhibitors (ICIs) or STING agonists, can reduce immunosuppression and strengthen anti-tumor immunity. *Abbreviations*: *LDH* lactate dehydrogenase, *MCT* Monocarboxylate transporter, *CD147* Cluster of differentiation 147, *MMP9* Matrix metalloproteinase 9, *HIF*-*1* Hypoxia-inducible factor 1, *Kla* Lysine lactylation, *VEGFR*-*2* Vascular endothelial growth factor receptor 2, *ICIs* Immune checkpoint inhibitors, *STING* Stimulator of interferon genes, *TAM* Tumor-associated macrophages, *M2* M2-like macrophages, *CTL* Cytotoxic T lymphocyte, *NK* Natural killer cell, *HDACs* Histone deacetylases, *PD*-*L1* Programmed death-ligand 1, *GPR132* G protein-coupled receptor 132

Lactate has also been involved in promoting the metastatic capacity of tumor cells by regulating intercellular adhesion, extracellular matrix degradation, and the establishment of a favorable microenvironment at metastatic sites. Researchers have found that extracellular acidification is linked with more aggressive tumor behaviors through upregulating ProMMP9/MMP9 expression, disrupting intercellular adhesion, and clearing obstacles for metastasis [217, 218]. The immunosuppressive nature of lactate impairs T-cell functionality and enables metastatic tumor cells to escape immune surveillance in secondary locations. It has also been observed that a high-lactate environment remodels the metabolic state of tumor-associated stromal cells, synergistically inducing angiogenesis, immunosuppression and metastatic stabilization, and setting the stage for distal metastasis [219]. In summary, lactate not only maintains the energy required for tumor growth by metabolic means, but also regulates angiogenesis and metastasis through a series of signaling pathways to provide structural and functional support for tumors. Therefore, interfering with lactate production or blocking its signaling pathway may become an effective strategy to inhibit abnormal tumor vascular development and distal metastasis (Fig. 11).

## Therapeutic targeting of lactate metabolism

The central role of lactate in the metabolic reprogramming of the TME is well-established, acting as both a key byproduct of glycolytic metabolism and a crucial modulator of various cellular signaling networks. In recent years, therapeutic strategies targeting lactate metabolism have gained increasing attention, emerging as a critical avenue in metabolic therapy and immunomodulation. These strategies are applicable to cancer treatment and show promising potential for conditions such as ischemia/reperfusion injury, neurodegenerative disorders, and cardiovascular disease.

### Tumor therapy

Considering lactate’s central function in metabolic reprogramming of the TME, lactate metabolism has emerged as a novel focus for tumor therapy. Therapeutic strategies targeting lactate metabolism fall into two major categories: direct modulators, which inhibit lactate production or transport by acting on LDHA/B or MCT1/4; and indirect modulators, which target upstream glycolytic regulators to reroute pyruvate toward mitochondrial oxidation or suppress hexokinase-mediated glycolysis [45, 220]. Notably, lactate transporters MCT1 and MCT4 require the chaperone protein CD147 (basigin) for membrane localization and functional activation. Inhibiting CD147, rather than MCTs directly, disrupts lactate transport by interfering with the disulfide-bonded domains in their Ig-like C2 region, highlighting its essential role in maintaining MCT catalytic activity [221].

A number of LDHA/B inhibitors, such as FX-11, Galloflavin, and N-hydroxyindole-based compounds, have been explored in animal or preclinical studies for cancer treatment, as summarized in Table 3. While some of these compounds have demonstrated anticancer activity in preclinical models, it is important to note that many, including galloflavin (largely abandoned due to poor bioavailability), have not progressed beyond this stage. Furthermore, the clinical translation of LDHA inhibitors as a class has been challenging, often hampered by issues of toxicity and lack of selectivity [222]. Therefore, while these preclinical findings are valuable for understanding LDHA as a target, their therapeutic readiness should not be overstated, and further research is crucial to assess their true clinical potential.

**Table 3:** Therapeutic targets and compounds modulating lactate metabolism

Therapeutic Agents	Targets	Diseases	Efficacy	Publish Year	Proof	NCT	Reference
FX−11	LDHA inhibitor	Lymphoma and pancreatic cancer	Inhibited LDHA using FX11 reduced ATP levels, induced oxidative stress, and caused cell death, showing efficacy in tumor regression, particularly in lymphoma and pancreatic cancer xenografts, suggesting a promising therapeutic strategy targeting the Warburg effect.	2010	Preclinical	NA	[257]
		Neuroblastoma	Inhibited aerobic glycolysis and growth in neuroblastoma cells by targeting LDHA with FX11, inducing modest G1 arrest and selective apoptosis, warranting further in vivo evaluation.	2017	Preclinical	NA	[258]
Galloflavin	LDHA/B inhibitor	Solid tumor	Inhibited aerobic glycolysis and induced apoptosis in tumor cells by targeting both LDHA and LDHB with galloflavin, a selective inhibitor, without affecting normal tissue respiration.	2012	Preclinical	NA	[259]
		Breast cancer	Inhibited proliferation of breast cancer cell lines, including aggressive and tamoxifen-resistant subtypes, by blocking glycolysis with galloflavin, inducing apoptosis through oxidative stress and downregulation of ERα signaling.	2012	Preclinical	NA	[260]
		Burkitt lymphoma	Inhibited Burkitt lymphoma cell replication by targeting LDH-A with galloflavin, reducing NAD levels and sirtuin−1 activity, leading to decreased MYC protein levels and suggesting potential for LDH inhibition in cancer therapy.	2013	Preclinical	NA	[261]
N-hydroxyindole-based compounds	LDHA inhibitor	Diversified tumors	Discovered N-hydroxyindole-based inhibitors of LDH-A that selectively target this enzyme, effectively reducing glucose-to-lactate conversion and showing potent antiproliferative activity, especially under hypoxic conditions.	2011	Preclinical	NA	[262]
ML-05	LDHA inhibitor	Melanoma	Inhibited tumor growth, and activated antitumor immunity in a melanoma model, with potential to enhance immunotherapies	2022	Preclinical	NA	[228]
NCI-006	LDHA inhibitor	Pancreatic cancer and colorectal cancer	Demonstrated metabolic rewiring and tumor growth inhibition through targeting LDH and mitochondrial complex 1 inhibition in glycolytic cancer models	2020	Preclinical	NA	[263]
PSTMB	LDHA inhibitor	NSCLC, breast cancer, liver cancer, melanoma, and colon cancer	Identified PSTMB as a potent LDHA inhibitor that reduced lactate production, inhibited tumor cell growth, and induced apoptosis in colon cancer cells, suggesting its potential as a novel anti-cancer drug targeting cancer metabolism	2019	Preclinical	NA	[264]
RS6212	LDHA inhibitor	Breast cancer, lung cancer, colon cancer, and pancreatic cancer	Inhibited tumor growth in multiple cancer cell lines with pyridazine derivative 18 (RS6212), showing potent anticancer activity and synergy with complex I inhibition	2022	Preclinical	NA	[265]
AZD3965	MCT1 inhibitor	Advanced Cancer	Inhibited lactate transport and demonstrated target engagement in advanced cancers with AZD3965, showing on-target pharmacodynamic activity and establishing a recommended phase II dose of 10 mg twice daily	2023	Phase I	NCT01791595	[67]
AZD0095	MCT4 inhibitor	Tumor (Not specified)	Inhibited MCT4 and induced cytotoxic levels of intracellular lactate with AZD0095, a potent and selective inhibitor, showing strong preclinical efficacy in combination with cediranib	2023	Preclinical	NA	[68]
Syrosingopine	MCT1/4 inhibitor	Tumor (Not specified)	Induced synthetic lethality and glycolytic blockade in cancer cells by inhibiting MCT1 and MCT4 with syrosingopine, in combination with metformin, leading to ATP depletion and cell death.	2018	Preclinical	NA	[69]
AR-C155858	MCT1 inhibitor	TNBC	Inhibited L-lactate uptake and cellular proliferation in TNBC cells with AR-C155858, but failed to reduce tumor growth or lactate concentrations in the 4T1 xenograft model, despite high drug concentrations in tumors	2018	Preclinical	NA	[223]
8G6	CD147*inhibitor	Breast cancer	Inhibited MMP−2 production and tumor cell invasion by blocking homophilic CD147 interactions with anti-CD147 antibody 8G6, suggesting potential therapeutic applications in preventing cancer metastasis	2001	Preclinical	NA	[226]
DCA	PDK inhibitor	Recurrent malignant brain tumors	Showed feasibility and tolerability of chronic oral DCA in recurrent malignant brain tumors, with no dose-limiting toxicities and clinical stability in patients over extended treatment periods	2014	Phase I	NCT01111097	[224]
Lonidamine	HK inhibitor	Malignant glioma	Inhibited aerobic glycolysis and showed moderate clinical activity in recurrent gliomas	1989	Phase II	NA	[225]
PKM2 inhibitors	PKM2	AD	Inhibited microglial activation and improved cognitive function in AD mice by disrupting the glycolysis/H4K12la/PKM2 feedback loop, suggesting a potential therapeutic approach for Alzheimer’s disease	2022	Preclinical	NA	[255]
MCT4 Inhibitor	MCT4, MPC inhibitor	Heart failure	Effectively reduced hypertrophy in cultured cardiomyocytes and mice	2021	Preclinical	NA	[137]
Half-molar Sodium Lactate	NA	Acute heart failure	Increased cardiac output and enhanced right ventricular systolic function without organ function harm	2014	Randomised controlled clinical trial	NCT01981655	[139]
L-lactate	NA	Obesity-associated type 2 diabetes	Reduced body weight gain and improved insulin sensitivity	2022	Preclinical	NA	[146]
Exogenous Lactate	NA	Inflammation, Macrophage Transition	Restored histone Kla and reparative gene expression (Arg1, Klf4) in BCAP-deficient mice	2020	Preclinical	NA	[173]
Oxamate	LDHA inhibitor	Brain injury after ischemic stroke	Inhibited lactate production or protein Kla formation reduced ischemic brain injury	2024	Preclinical	NA	[251]
Stiripentol	LDH inhibitor	Epilepsy	Suppressed seizures and epileptiform activity by inhibiting LDH, with a new LDH inhibitor identified that effectively reduced seizures in vivo, suggesting LDH inhibitors as a promising class of antiepileptic drugs.	2015	Approved by FDA	Approved by FDA	[253]
Exogenous Lactate	NA	Cerebral ischemic stroke	Lactate transport into neurons enhances neuroprotection by supporting ATP-starved hypoxic neurons	2024	Preclinical	NA	[187]
Lactate supplementation	Mitochondrial enzyme activity	Exercise physiology, mitochondrial function	Enhanced mitochondrial enzyme activity in oxidative muscle; improved energy production and acid-base balance during exercise	2020	Preclinical	NA	[235]
Lactate supplementation	Gut microbiota	Gut dysbiosis, microbial balance	Promoted beneficial bacteria (*Lactobacillus*,* Bifidobacterium*), suppressing potential pathogens like *Clostridium*	2024	Preclinical	NA	[243]
lactic acid-producing bacterium	Butyrate-producing bacteria	Inflammatory bowel disease	Alleviated colitis via expanding butyrate producers and suppressing IL−6/STAT3 signaling	2021	Preclinical	NA	[248]

This table provides a comprehensive overview of representative inhibitors targeting key nodes in lactate metabolism, such as lactate dehydrogenase (LDH) and monocarboxylate transporters (MCTs). It includes compounds at various developmental stages, from preclinical candidates to those in clinical trials. While most of the listed compounds have not yet advanced beyond clinical stages, and some have been abandoned due to poor pharmacokinetic properties, this table serves as a broad resource on emerging therapeutic strategies targeting lactate metabolism. Not all compounds are current clinical development candidates. *CD147 forms stable, non-covalent complexes with MCT1 and MCT4, serving as an essential chaperone for their proper folding and trafficking to the plasma membrane

Abbreviations: *SCLC* Small cell lung cancer, *LDH* Lactate dehydrogenase, *MCH* Monocarboxylate transporter, *SCLC* Small cell lung cancer, *NSCLC* Non-small cell lung cancer, *TNBC* Triple negative breast cancer, *PDK* Pyruvate dehydrogenase kinase, *CD147* Cluster of Differentiation 147, *DCA* Dichloroacetate, *AD* Alzheimer’s disease, *MPC* Mitochondrial pyruvate carrier, *FDA* U.S. Food and Drug Administration

Similarly, several novel MCT1/4 inhibitors are under investigation, including AZD3965, which has demonstrated anti-tumor effects by inhibiting MCT1, disrupting lactate metabolism in tumors, and causing lactate accumulation within cancer cells. This leads to a decrease in intracellular pH, thereby inhibiting tumor cell growth and proliferation. Additionally, AZD3965 may affect the tumor microenvironment by disrupting the metabolic coupling between tumor cells and stromal cells. The Phase I clinical study confirmed the feasibility of this mechanism and laid the foundation for further research in tumor types with high MCT1 and low MCT4 expression, such as certain lymphomas. However, its true therapeutic efficacy will need to be further validated in biomarker-selected patient populations [67]. However, its true therapeutic efficacy will need to be further validated in biomarker-selected patient populations. Most of the other MCT1/4 inhibitors remain in preclinical stages and have not yet been tested in clinical trials, and further validation in clinical settings is required to establish their therapeutic potential [68, 69, 223].

Of note, targeting lactate production upstream represents a viable therapeutic strategy. This approach is exemplified by Dichloroacetate, an inhibitor of pyruvate dehydrogenase kinase that diverts pyruvate away from lactate conversion and into mitochondria, and by Lonidamine, which suppresses glycolysis at its origin by inhibiting mitochondrially-bound hexokinase. Clinical studies have demonstrated the feasibility of both approaches, with oral DCA being well-tolerated in patients with recurrent malignant brain tumors and Lonidamine showing modest efficacy in a Phase II study for recurrent glioma, thereby providing a clinical foundation for targeting glycolytic flux to suppress tumorigenic lactate generation [224, 225].

Beyond modulating the metabolic and immune environments of tumors, targeting tumor matrix remodeling has emerged as a novel therapeutic strategy. CD147 (EMMPRIN) is a glycosylated membrane protein highly expressed on the surface of tumor cells. It induces the synthesis of matrix metalloproteinases (MMPs) through homodimeric binding and promotes tumor invasion. Blocking CD147 homophilic binding significantly inhibits MMP-2 secretion and tumor cell invasion, suggesting its potential as a target for anti-metastatic therapy [226]. Moreover, CD147 can directly enhance the angiogenic capacity of endothelial cells by upregulating hypoxia-inducible factor 2 alpha (HIF-2α), vascular endothelial growth factor receptor 2 (VEGFR-2), and soluble VEGF isoforms. This provides new insights into the mechanisms of CD147-mediated tumor angiogenesis and highlights CD147 as a potential target for anti-angiogenic therapies [227].

In recent years, the advantages of combining lactate inhibitors with immunotherapy in antitumor treatment have become increasingly evident. In particular, when used in conjunction with immune checkpoint inhibitors (ICIs) and Stimulator of Interferon Genes (STING) agonists, lactate inhibitors can significantly decrease tumor immunosuppression and promote the activation of tumor-infiltrating T cells [228, 229]. Through this combination strategy, the immune escape mechanism in the TME can be overcome and the immune system’s capacity to clear the tumor can be enhanced. This dual treatment approach enhances the durability and efficacy of immunotherapy. Consequently, the combined application of lactate inhibitors and immunotherapy shows great clinical promise and opens up new avenues for cancer treatment [230, 231]. Collectively, this evidence underscores the pivotal role of lactate in tumor progression and therapy, as illustrated in Fig. 11.

### Therapeutic approaches for Non‑oncological conditions

As previously described, lactate has emerged as a vital mediator in various non-cancerous pathologies, where it functions as more than just a product of metabolism, serving as a signaling agent and modulating the microenvironment. Accordingly, a growing number of targeted intervention strategies have emerged, offering new therapeutic avenues and broad prospects for the treatment of non-tumor diseases.

#### Athletic performance and metabolic regulation

The role of lactate in facilitating exercise adaptation is rooted in its triple identity as an energy substrate, a signaling molecule, and, most recently, an epigenetic regulator. This multifaceted nature means that the lactate surge during exercise does not simply represent a metabolic byproduct; rather, it actively orchestrates a wide range of cellular and molecular responses that underpin long-term training adaptations [232].

As a central component of the cell-to-cell and intracellular lactate shuttles, lactate ensures efficient energy distribution from its production sites such as glycolytic muscle fibers to oxidative tissues, including heart, brain, and oxidative muscle fibers, where it serves as a primary fuel for mitochondrial oxidative phosphorylation [233, 234]. Long-term sodium lactate supplementation has been shown to increase mitochondrial enzyme activity in skeletal muscle, underscoring its role in mitochondrial adaptation during exercise [235]. Beyond its energetic role, lactate-derived lactylation has emerged as a key mechanism fine-tuning metabolic and adaptive processes. For instance, mitochondrial protein lactylation, such as on PDHA1 and CPT2, may transiently constrain oxidative metabolism during exhaustive exercise as a protective mechanism against oxidative damage [74].

Furthermore, lactate acts as a critical mediator along the muscle-brain axis. Exercise-induced lactate can cross the blood-brain barrier and be taken up by neurons and astrocytes. Critically, this circulatory lactate integrates into the well-established ANLS theory within the hippocampus, a process fundamental to learning and memory. Disruption of this lactate shuttle at either the astrocytic (MCT4) or neuronal (MCT2) level leads to spatial memory deficits, as demonstrated in hippocampus-dependent tasks. Notably, these impairments can be rescued by exogenous L-lactate in MCT4-deficient but not MCT2-deficient mice, indicating that lactate transfer from astrocytes and its utilization in neurons are both essential for memory acquisition. In addition, neuronal MCT2 plays a unique role in long-term memory consolidation, and its knockdown in mature neurons also impairs hippocampal neurogenesis, revealing that MCT2 function extends beyond metabolic support to the regulation of plasticity-related processes. These findings underscore the indispensable and distinct roles of astroglial MCT4 and neuronal MCT2 in lactate-mediated cognitive function [47, 236].

However, the signaling cascade that triggers lactate release for memory consolidation is sophisticated. It is initiated by emotionally salient experiences that engage noradrenaline, which then acts specifically on hippocampal astrocytic β2-adrenergic receptors (β2ARs) [237]. This receptor activation is coupled to the training-dependent release of lactate from astrocytes, which is necessary for both long-term memory formation and the underlying molecular changes, such as the induction of phospho-CREB and Arc [47, 237]. Furthermore, optogenetic studies demonstrate that increasing cAMP levels specifically in hippocampal astrocytes is sufficient to modulate memory, an effect that is mediated by ANLS[238]. Building on these findings, the lactate surge induced by exercise is poised to emulate or augment these endogenous neurochemical processes. By supplying a supplementary flux of lactate to the brain, exercise may directly fuel the as ANLS, thereby facilitating the synaptic plasticity, gene expression, and neurogenesis that underlie the acquisition and consolidation of long-term memory [47, 236]. This establishes lactate as a pivotal molecular link that translates physical activity into enhanced cognitive function.

In parallel, the potential of oral lactate as a nutraceutical has garnered interest. Serving as a key energy source, signaling agent, and metabolic regulator, oral lactate is quickly absorbed into the bloodstream and skeletal muscles, enhancing oxidative metabolism [239]. In the liver, it may be transformed into glucose and stored as glycogen, while consuming hydrogen ions to help maintain bicarbonate levels and enhance extracellular pH buffering [233]. Therefore, lactate supports energy production and acid-base balance during exercise. Recent studies further suggest that lactate supplementation may enhance mitochondrial enzyme activity in oxidative muscle, thereby improving mitochondrial function [235]. Nevertheless, its physiological impacts appear to be multifaceted and context-sensitive, highlighting the need for further targeted investigation. While lactate exhibits clear buffering properties by increasing blood pH and bicarbonate levels, beneficial in short-duration, high-intensity efforts, it fails to improve endurance performance under lower-intensity or prolonged exercise conditions [240]. This dualistic outcome likely stems from the distinct physiological roles of lactate and associated acidosis: elevated lactate itself may not impair muscle function and might even support performance, yet the concomitant severe intracellular acidosis (pH 6.5–6.2), especially in fast-twitch fibers, reduces calcium sensitivity and impairs myosin ATPase activity, ultimately inducing muscle fatigue. Such conflicting observations are exemplified in studies where lactate supplementation improved acid-base balance and lowered perceived exertion without enhancing actual performance output. For instance, one trial on high-intensity interval cycling reported no performance gains despite improved buffering [241]. Similarly, another clinical study involving recreational exercisers found that oral lactate failed to improve aerobic capacity or lactate threshold, though it modestly increased work rate during a 20-minute time trial [242]. These findings underscore the importance of context, dosage regimen, and metabolic environment, reinforcing that the systemic benefits of lactate may be counteracted by localized biochemical disturbances in muscle tissue.

#### The lactate-microbiome-host axis

The regulatory role of lactate extends beyond cellular metabolism and systemic signaling to encompass profound influence on the gut ecosystem. This is evidenced by its significant prebiotic potential in shaping gut microbiota composition. A comprehensive meta-analysis reveals that lactate supplementation selectively enriches beneficial gut bacteria, such as *Lactobacillus* and *Bifidobacterium*, while reducing the *Firmicutes-to-Bacteroidetes* ratio and suppressing potential pathogens like *Clostridium*. Critically, these effects exhibit a clear dose-dependency, with pronounced benefits for *Lactobacillus* at doses exceeding 2,000 mg/day and for *Bifidobacterium* across a wide range (10 − 5,500 mg/day). Moreover, the response is age-dependent, with young adults demonstrating more consistent and robust improvements compared to older adults. This positions lactate as a promising agent for improving gut health and microbial balance [243].

The pivotal role of lactate-producing and consuming bacteria in gut and systemic health is particularly evident in the context of inflammatory bowel disease (IBD). Lactate-producing bacteria, such as *Lactobacillus* and *Bifidobacterium*, play a key role in the human gut microbiota and have shown effectiveness in alleviating IBD by improving intestinal permeability, reducing inflammation, and importantly, influencing the composition of the gut microbial community [244, 245]. A key mechanism underpinning their benefit is microbial cross-feeding. Lactate produced by bacterial community serves as a critical energy substrate for other commensal bacteria, such as butyrate-producing species within *Clostridium* groups XIVa and IV (e.g., *Faecalibacterium* and *Roseburia*) [246, 247]. This lactate-to-butyrate metabolic pathway is essential for maintaining colonic health, as butyrate serves as a primary energy source for colonocytes, strengthens the epithelial barrier, and exerts potent anti-inflammatory effects [248].

Conversely, dysbiosis in IBD is characterized by a depletion of these beneficial short-chain fatty acid (SCFA)-producing bacteria and an expansion of potential pathobionts like *Escherichia coli* [249, 250]. Interventions with specific lactate-producing strains, such as *Levilactobacillus brevis* and *Lactobacillus paracasei*, have been shown to counteract this dysbiosis by increasing the abundance of SCFA-producing bacteria and reducing pro-inflammatory cytokines, thereby restoring a healthier microbial ecosystem and alleviating colitis [247, 248]. Therefore, the therapeutic potential of lactate and lactate-producing bacterial community extends beyond their direct actions, lying significantly in their capacity to nourish a cooperative microbial network through cross-feeding, which collectively stabilizes gut homeostasis and immune function.

#### Other diseases

Lactate metabolism plays diverse roles across a range of diseases beyond cancer, including cardiovascular, neurological, metabolic, and inflammatory conditions. In ischemia-reperfusion injury, lactate supports neuronal survival via ANLS and histone lactylation modulation [13, 49, 251]. In obesity-associated insulin resistance, lactate accumulation due to impaired MCT1-mediated efflux contributes to inflammation and adipocyte dysfunction [144, 252]. In cardiovascular disease, lactate exerts both metabolic and signaling effects, while in epilepsy, LDH inhibitors targeting astrocyte-derived lactate have demonstrated anti-seizure activity approved by the U.S. Food and Drug Administration (FDA) [68, 136–139, 253]. Therapeutic strategies involving lactate modulation have shown promising effects across these disease contexts, and a summary of representative lactate-targeted therapies across multiple disease models is provided in Table 3.

## Conclusive remarks

Lactate was once thought to be just a byproduct of metabolism; however, with advancements in molecular biology and immunometabolism, growing evidence now shows that lactate plays essential and diverse roles in supporting human health and regulating disease mechanisms. From a basic substrate of energy metabolism to a key epigenetic regulator, from a guardian of physiological homeostasis to a promoter of disease development, the biological functions of lactate are far beyond the traditional understanding.

Under physiological conditions, lactate is not only a supplementary energy source for OXPHOS in muscle, brain, liver and other tissues, but also transmits metabolic information between cells through the lactate shuttle to maintain redox homeostasis. Lactate can move in and out of cells via MCTs, enter mitochondria to take part in the TCA cycle or be reprocessed in the liver through the Cori cycle; in addition, it acts on histones and non-histone proteins via lactylation modifications to play epigenetic regulatory roles in inflammatory repair, embryonic development and immune cell reprogramming.

Under pathological conditions, lactate accumulation and metabolic reprogramming have been increasingly recognized as critical contributors to a wide range of human diseases. Elevated lactate levels are observed not limited to tumors, but also widespread in various non-tumor diseases such as immune, cardiovascular, metabolic, neurological and infectious diseases. In these contexts, lactate is no longer simply a metabolic by-product, but a crucial element affecting disease evolution, prognosis and systemic homeostasis. Abnormal lactate levels are closely associated with chronic inflammation, tissue hypoxia, metabolic imbalance, and impaired immune regulation, highlighting its potential as a disease marker and regulator.

In conclusion, while the multifaceted roles of lactate in physiology and disease are increasingly clear from preclinical work, a significant translational gap remains. Future research must not only deepen our mechanistic understanding but also prioritize rigorous human studies to evaluate the safety and efficacy of lactate-targeted therapies. Bridging this gap is the essential next step to harnessing the biology of lactate for clinical benefit.

## Data Availability

No datasets were generated or analysed during the current study.

## References

[1] Lee S, Choi Y, Jeong E, Park J, Kim J, Tanaka M, et al. Physiological significance of elevated levels of lactate by exercise training in the brain and body. J Biosci Bioeng. 2023;135:167–75. 10.1016/j.jbiosc.2022.12.001. 36681523 10.1016/j.jbiosc.2022.12.001

[2] Gladden LB. Lactate metabolism: a new paradigm for the third millennium. J Physiol. 2004;558:5–30. 10.1113/jphysiol.2003.058701. 15131240 10.1113/jphysiol.2003.058701 PMC1664920

[3] Brooks GA. Lactate production under fully aerobic conditions: the lactate shuttle during rest and exercise. Fed Proc. 1986;45:2924–9. 3536591

[4] Li X, Yang Y, Zhang B, Lin X, Fu X, An Y, et al. Lactate metabolism in human health and disease. Signal Transduct Target Ther. 2022;7:305. 10.1038/s41392-022-01151-3. 36050306 10.1038/s41392-022-01151-3 PMC9434547

[5] Teng R, Liu Z, Tang H, Zhang W, Chen Y, Xu R, et al. HSP60 Silencing promotes Warburg-like phenotypes and switches the mitochondrial function from ATP production to biosynthesis in CcRCC cells. Redox Biol. 2019;24:101218. 10.1016/j.redox.2019.101218. 31112866 10.1016/j.redox.2019.101218 PMC6526248

[6] Rafikov R, Sun X, Rafikova O, Louise Meadows M, Desai AA, Khalpey Z, et al. Complex I dysfunction underlies the glycolytic switch in pulmonary hypertensive smooth muscle cells. Redox Biol. 2015;6:278–86. 10.1016/j.redox.2015.07.016. 26298201 10.1016/j.redox.2015.07.016 PMC4556771

[7] Chen Z, Liu M, Li L, Chen L. Involvement of the Warburg effect in non-tumor diseases processes. J Cell Physiol. 2018;233:2839–49. 10.1002/jcp.25998. 28488732 10.1002/jcp.25998

[8] Brooks GA, The Science and Translation of Lactate Shuttle Theory. Cell Metab. 2018;27:757–85. 10.1016/j.cmet.2018.03.008. 29617642 10.1016/j.cmet.2018.03.008

[9] Hashimoto T, Hussien R, Oommen S, Gohil K, Brooks GA. Lactate sensitive transcription factor network in L6 cells: activation of MCT1 and mitochondrial biogenesis. Faseb J. 2007;21:2602–12. 10.1096/fj.07-8174com. 17395833 10.1096/fj.07-8174com

[10] Luengo A, Li Z, Gui DY, Sullivan LB, Zagorulya M, Do BT, et al. Increased demand for NAD(+) relative to ATP drives aerobic Glycolysis. Mol Cell. 2021;81:691–e707696. 10.1016/j.molcel.2020.12.012. 33382985 10.1016/j.molcel.2020.12.012 PMC8315838

[11] Yoshida GJ. Metabolic reprogramming: the emerging concept and associated therapeutic strategies. J Exp Clin Cancer Res. 2015;34:111. 10.1186/s13046-015-0221-y. 26445347 10.1186/s13046-015-0221-y PMC4595070

[12] Brown TP, Ganapathy V. Lactate/GPR81 signaling and proton motive force in cancer: role in angiogenesis, immune escape, nutrition, and Warburg phenomenon. Pharmacol Ther. 2020;206:107451. 10.1016/j.pharmthera.2019.107451. 31836453 10.1016/j.pharmthera.2019.107451

[13] Sotelo-Hitschfeld T, Niemeyer MI, Mächler P, Ruminot I, Lerchundi R, Wyss MT, et al. Channel-mediated lactate release by K⁺-stimulated astrocytes. J Neurosci. 2015;35:4168–78. 10.1523/jneurosci.5036-14.2015. 25762664 10.1523/JNEUROSCI.5036-14.2015 PMC6605297

[14] Karagiannis A, Sylantyev S, Hadjihambi A, Hosford PS, Kasparov S, Gourine AV. Hemichannel-mediated release of lactate. J Cereb Blood Flow Metab. 2016;36:1202–11. 10.1177/0271678x15611912. 26661210 10.1177/0271678X15611912 PMC4900446

[15] Zhang D, Tang Z, Huang H, Zhou G, Cui C, Weng Y, et al. Metabolic regulation of gene expression by histone lactylation. Nature. 2019;574:575–80. 10.1038/s41586-019-1678-1. 31645732 10.1038/s41586-019-1678-1 PMC6818755

[16] Llibre A, Kucuk S, Gope A, Certo M, Mauro C, Lactate. A key regulator of the immune response. Immunity. 2025;58:535–54. 10.1016/j.immuni.2025.02.008. 40073846 10.1016/j.immuni.2025.02.008

[17] Gobelet C, Gerster JC. Synovial fluid lactate levels in septic and non-septic arthritides. Ann Rheum Dis. 1984;43:742–5. 10.1136/ard.43.5.742. 6497466 10.1136/ard.43.5.742 PMC1001519

[18] Ghosh-Choudhary S, Finkel T. Lactylation regulates cardiac function. Cell Res. 2023;33:653–4. 10.1038/s41422-023-00857-5. 37488305 10.1038/s41422-023-00857-5 PMC10474038

[19] Park SY, Jung SR, Kim JY, Kim YW, Sung HK, Park SY, et al. Lactate promotes fatty acid oxidation by the TCA cycle and mitochondrial respiration in muscles of obese mice. Am J Physiol Cell Physiol. 2024. 10.1152/ajpcell.00060.2024. 38981606 10.1152/ajpcell.00060.2024

[20] Louis P, Duncan SH, Sheridan PO, Walker AW, Flint HJ. Microbial lactate utilisation and the stability of the gut Microbiome. Gut Microbiome (Camb). 2022;3:e3. 10.1017/gmb.2022.3. 39295779 10.1017/gmb.2022.3 PMC11406415

[21] San-Millán I, Julian CG, Matarazzo C, Martinez J, Brooks GA. Is lactate an oncometabolite? Evidence supporting a role for lactate in the regulation of transcriptional activity of cancer-Related genes in MCF7 breast cancer cells. Front Oncol. 2019;9:1536. 10.3389/fonc.2019.01536. 32010625 10.3389/fonc.2019.01536 PMC6971189

[22] Wei S, Zhang J, Zhao R, Shi R, An L, Yu Z, et al. Histone lactylation promotes malignant progression by facilitating USP39 expression to target PI3K/AKT/HIF-1α signal pathway in endometrial carcinoma. Cell Death Discov. 2024;10:121. 10.1038/s41420-024-01898-4. 38459014 10.1038/s41420-024-01898-4 PMC10923933

[23] Judith A, Wisneski E, Gertz W. Dual carbon-labeled isotope experiments using D-[6-14 C] glucose and L-[1,2,3-13C3] lactate: a new approach for investigating human myocardial metabolism during ischemia. J Am Coll Cardiol. 1985;5:5. 10.1016/s0735-1097(85)80016-4 3989125

[24] Meyer C, Stumvoll M, Dostou J, Welle S, Haymond M, Gerich J. Renal substrate exchange and gluconeogenesis in normal postabsorptive humans. Am J Physiol Endocrinol Metab. 2002;282:E428–434. 10.1152/ajpendo.00116.2001. 11788376 10.1152/ajpendo.00116.2001

[25] Huang X, Li Y, Fu M, Xin HB. Polarizing macrophages in vitro. Methods Mol Biol. 2018;1784:119–26. 10.1007/978-1-4939-7837-3_12. 29761394 10.1007/978-1-4939-7837-3_12 PMC8875934

[26] Metallo CM, Gameiro PA, Bell EL, Mattaini KR, Yang J, Hiller K, et al. Reductive glutamine metabolism by IDH1 mediates lipogenesis under hypoxia. Nature. 2011;481:380–4. 10.1038/nature10602. 22101433 10.1038/nature10602 PMC3710581

[27] DeBerardinis RJ, Mancuso A, Daikhin E, Nissim I, Yudkoff M, Wehrli S, et al. Beyond aerobic glycolysis: transformed cells can engage in glutamine metabolism that exceeds the requirement for protein and nucleotide synthesis. Proc Natl Acad Sci U S A. 2007;104:19345–50. 10.1073/pnas.0709747104. 18032601 10.1073/pnas.0709747104 PMC2148292

[28] Hui S, Ghergurovich JM, Morscher RJ, Jang C, Teng X, Lu W, et al. Glucose feeds the TCA cycle via Circulating lactate. Nature. 2017;551:115–8. 10.1038/nature24057. 29045397 10.1038/nature24057 PMC5898814

[29] Faubert B, Li KY, Cai L, Hensley CT, Kim J, Zacharias LG, et al. Lactate metabolism in human lung tumors. Cell. 2017;171:358–e371359. 10.1016/j.cell.2017.09.019. 28985563 10.1016/j.cell.2017.09.019 PMC5684706

[30] Bergman BC, Wolfel EE, Butterfield GE, Lopaschuk GD, Casazza GA, Horning MA, et al. Active muscle and whole body lactate kinetics after endurance training in men. J Appl Physiol (1985). 1999;87:1684–96. 10.1152/jappl.1999.87.5.1684. 10562610 10.1152/jappl.1999.87.5.1684

[31] Hashimoto T, Hussien R, Brooks GA. Colocalization of MCT1, CD147, and LDH in mitochondrial inner membrane of L6 muscle cells: evidence of a mitochondrial lactate oxidation complex. Am J Physiol Endocrinol Metab. 2006;290:E1237–1244. 10.1152/ajpendo.00594.2005. 16434551 10.1152/ajpendo.00594.2005

[32] Glancy B, Kane DA, Kavazis AN, Goodwin ML, Willis WT, Gladden LB. Mitochondrial lactate metabolism: history and implications for exercise and disease. J Physiol. 2021;599:863–88. 10.1113/jp278930. 32358865 10.1113/JP278930 PMC8439166

[33] Yang J, Ruchti E, Petit JM, Jourdain P, Grenningloh G, Allaman I, et al. Lactate promotes plasticity gene expression by potentiating NMDA signaling in neurons. Proc Natl Acad Sci U S A. 2014;111:12228–33. 10.1073/pnas.1322912111. 25071212 10.1073/pnas.1322912111 PMC4143009

[34] Quinn WJ 3rd, Jiao J, TeSlaa T, Stadanlick J, Wang Z, Wang L, et al. Lactate limits T cell proliferation via the NAD(H) redox state. Cell Rep. 2020;33:108500. 10.1016/j.celrep.2020.108500. 10.1016/j.celrep.2020.108500 PMC7830708 33326785

[35] Halestrap AP. The SLC16 gene family - structure, role and regulation in health and disease. Mol Aspects Med. 2013;34:337–49. 10.1016/j.mam.2012.05.003. 23506875 10.1016/j.mam.2012.05.003

[36] Halestrap AP, Meredith D. The SLC16 gene family-from monocarboxylate transporters (MCTs) to aromatic amino acid transporters and beyond. Pflugers Arch. 2004;447:619–28. 10.1007/s00424-003-1067-2. 12739169 10.1007/s00424-003-1067-2

[37] Felmlee MA, Jones RS, Rodriguez-Cruz V, Follman KE, Morris ME. Monocarboxylate transporters (SLC16): Function, Regulation, and role in health and disease. Pharmacol Rev. 2020;72:466–85. 10.1124/pr.119.018762. 32144120 10.1124/pr.119.018762 PMC7062045

[38] Sun X, Wang M, Wang M, Yao L, Li X, Dong H, et al. Role of Proton-Coupled monocarboxylate transporters in cancer: from metabolic crosstalk to therapeutic potential. Front Cell Dev Biol. 2020;8:651. 10.3389/fcell.2020.00651. 32766253 10.3389/fcell.2020.00651 PMC7379837

[39] Brown TP, Bhattacharjee P, Ramachandran S, Sivaprakasam S, Ristic B, Sikder MOF, et al. The lactate receptor GPR81 promotes breast cancer growth via a paracrine mechanism involving antigen-presenting cells in the tumor microenvironment. Oncogene. 2020;39:3292–304. 10.1038/s41388-020-1216-5. 32071396 10.1038/s41388-020-1216-5

[40] Ahmed K, Tunaru S, Tang C, Müller M, Gille A, Sassmann A, et al. An autocrine lactate loop mediates insulin-dependent Inhibition of lipolysis through GPR81. Cell Metab. 2010;11:311–9. 10.1016/j.cmet.2010.02.012. 20374963 10.1016/j.cmet.2010.02.012

[41] Brooks GA. Cell-cell and intracellular lactate shuttles. J Physiol. 2009;587:5591–600. 10.1113/jphysiol.2009.178350. 19805739 10.1113/jphysiol.2009.178350 PMC2805372

[42] Chandel NS. Mitochondria as signaling organelles. BMC Biol. 2014;12:34. 10.1186/1741-7007-12-34. 24884669 10.1186/1741-7007-12-34 PMC4035690

[43] Bosshart PD, Kalbermatter D, Bonetti S, Fotiadis D. Mechanistic basis of L-lactate transport in the SLC16 solute carrier family. Nat Commun. 2019;10:2649. 10.1038/s41467-019-10566-6. 31201333 10.1038/s41467-019-10566-6 PMC6573034

[44] Wilson MC, Meredith D, Bunnun C, Sessions RB, Halestrap AP. Studies on the DIDS-binding site of monocarboxylate transporter 1 suggest a homology model of the open conformation and a plausible translocation cycle. J Biol Chem. 2009;284:20011–21. 10.1074/jbc.M109.014217. 19473976 10.1074/jbc.M109.014217 PMC2740427

[45] Doherty JR, Cleveland JL. Targeting lactate metabolism for cancer therapeutics. J Clin Invest. 2013;123:3685–92. 10.1172/jci69741. 23999443 10.1172/JCI69741 PMC3754272

[46] Lin RY, Vera JC, Chaganti RS, Golde DW. Human monocarboxylate transporter 2 (MCT2) is a high affinity pyruvate transporter. J Biol Chem. 1998;273:28959–65. 10.1074/jbc.273.44.28959. 9786900 10.1074/jbc.273.44.28959

[47] Suzuki A, Stern SA, Bozdagi O, Huntley GW, Walker RH, Magistretti PJ, et al. Astrocyte-neuron lactate transport is required for long-term memory formation. Cell. 2011;144:810–23. 10.1016/j.cell.2011.02.018. 21376239 10.1016/j.cell.2011.02.018 PMC3073831

[48] Mächler P, Wyss MT, Elsayed M, Stobart J, Gutierrez R, von Faber-Castell A, et al. In vivo evidence for a lactate gradient from astrocytes to neurons. Cell Metab. 2016;23:94–102. 10.1016/j.cmet.2015.10.010. 26698914 10.1016/j.cmet.2015.10.010

[49] Yang C, Pan RY, Guan F, Yuan Z. Lactate metabolism in neurodegenerative diseases. Neural Regen Res. 2024;19:69–74. 10.4103/1673-5374.374142. 37488846 10.4103/1673-5374.374142 PMC10479854

[50] Descalzi G, Gao V, Steinman MQ, Suzuki A, Alberini CM. Lactate from astrocytes fuels learning-induced mRNA translation in excitatory and inhibitory neurons. Commun Biol. 2019;2:247. 10.1038/s42003-019-0495-2. 31286064 10.1038/s42003-019-0495-2 PMC6606643

[51] Wu A, Lee D, Xiong WC. Lactate Metabolism, Signaling, and function in brain Development, synaptic Plasticity, Angiogenesis, and neurodegenerative diseases. Int J Mol Sci. 2023;24. 10.3390/ijms241713398. 10.3390/ijms241713398 PMC10487923 37686202

[52] Zhang M, Cheng X, Dang R, Zhang W, Zhang J, Yao Z. Lactate deficit in an alzheimer disease mouse model: the relationship with neuronal damage. J Neuropathol Exp Neurol. 2018;77:1163–76. 10.1093/jnen/nly102. 30383244 10.1093/jnen/nly102

[53] Dimmer KS, Friedrich B, Lang F, Deitmer JW, Bröer S. The low-affinity monocarboxylate transporter MCT4 is adapted to the export of lactate in highly glycolytic cells. Biochem J. 2000; 350 Pt 1: 219–27. PMC1221245 10926847

[54] Park SJ, Smith CP, Wilbur RR, Cain CP, Kallu SR, Valasapalli S, et al. An overview of MCT1 and MCT4 in GBM: small molecule transporters with large implications. Am J Cancer Res. 2018;8:1967–76. 30416849 PMC6220151

[55] Kirk P, Wilson MC, Heddle C, Brown MH, Barclay AN, Halestrap AP. CD147 is tightly associated with lactate transporters MCT1 and MCT4 and facilitates their cell surface expression. Embo J. 2000;19:3896–904. 10.1093/emboj/19.15.3896. 10921872 10.1093/emboj/19.15.3896 PMC306613

[56] Deora AA, Philp N, Hu J, Bok D, Rodriguez-Boulan E. Mechanisms regulating tissue-specific Polarity of monocarboxylate transporters and their chaperone CD147 in kidney and retinal epithelia. Proc Natl Acad Sci U S A. 2005;102:16245–50. 10.1073/pnas.0504419102. 16260747 10.1073/pnas.0504419102 PMC1283422

[57] Webb BA, Chimenti M, Jacobson MP, Barber DL. Dysregulated pH: a perfect storm for cancer progression. Nat Rev Cancer. 2011;11:671–7. 10.1038/nrc3110. 21833026 10.1038/nrc3110

[58] Singh L, Nair L, Kumar D, Arora MK, Bajaj S, Gadewar M, et al. Hypoxia induced lactate acidosis modulates tumor microenvironment and lipid reprogramming to sustain the cancer cell survival. Front Oncol. 2023;13:1034205. 10.3389/fonc.2023.1034205. 36761981 10.3389/fonc.2023.1034205 PMC9906992

[59] Ullah MS, Davies AJ, Halestrap AP. The plasma membrane lactate transporter MCT4, but not MCT1, is up-regulated by hypoxia through a HIF-1alpha-dependent mechanism. J Biol Chem. 2006;281:9030–7. 10.1074/jbc.M511397200. 16452478 10.1074/jbc.M511397200

[60] Végran F, Boidot R, Michiels C, Sonveaux P, Feron O. Lactate influx through the endothelial cell monocarboxylate transporter MCT1 supports an NF-κB/IL-8 pathway that drives tumor angiogenesis. Cancer Res. 2011;71:2550–60. 10.1158/0008-5472.Can-10-2828. 21300765 10.1158/0008-5472.CAN-10-2828

[61] Sonveaux P, Végran F, Schroeder T, Wergin MC, Verrax J, Rabbani ZN, et al. Targeting lactate-fueled respiration selectively kills hypoxic tumor cells in mice. J Clin Invest. 2008;118:3930–42. 10.1172/jci36843. 19033663 10.1172/JCI36843 PMC2582933

[62] De Saedeleer CJ, Copetti T, Porporato PE, Verrax J, Feron O, Sonveaux P. Lactate activates HIF-1 in oxidative but not in Warburg-phenotype human tumor cells. PLoS ONE. 2012;7:e46571. 10.1371/journal.pone.0046571. 23082126 10.1371/journal.pone.0046571 PMC3474765

[63] Pavlides S, Vera I, Gandara R, Sneddon S, Pestell RG, Mercier I, et al. Warburg Meets autophagy: cancer-associated fibroblasts accelerate tumor growth and metastasis via oxidative stress, mitophagy, and aerobic Glycolysis. Antioxid Redox Signal. 2012;16:1264–84. 10.1089/ars.2011.4243. 21883043 10.1089/ars.2011.4243 PMC3324816

[64] Witkiewicz AK, Whitaker-Menezes D, Dasgupta A, Philp NJ, Lin Z, Gandara R, et al. Using the reverse Warburg effect to identify high-risk breast cancer patients: stromal MCT4 predicts poor clinical outcome in triple-negative breast cancers. Cell Cycle. 2012;11:1108–17. 10.4161/cc.11.6.19530. 22313602 10.4161/cc.11.6.19530 PMC3335917

[65] Sotgia F, Martinez-Outschoorn UE, Lisanti MP. The reverse Warburg effect in osteosarcoma. Oncotarget. 2014;5:7982–3. 10.18632/oncotarget.2352. 25478627 10.18632/oncotarget.2352 PMC4226660

[66] Fu Y, Liu S, Yin S, Niu W, Xiong W, Tan M, et al. The reverse Warburg effect is likely to be an achilles’ heel of cancer that can be exploited for cancer therapy. Oncotarget. 2017;8:57813–25. 10.18632/oncotarget.18175. 28915713 10.18632/oncotarget.18175 PMC5593685

[67] Halford S, Veal GJ, Wedge SR, Payne GS, Bacon CM, Sloan P, et al. A phase I Dose-escalation study of AZD3965, an oral monocarboxylate transporter 1 Inhibitor, in patients with advanced cancer. Clin Cancer Res. 2023;29:1429–39. 10.1158/1078-0432.Ccr-22-2263. 36652553 10.1158/1078-0432.CCR-22-2263 PMC7614436

[68] Goldberg FW, Kettle JG, Lamont GM, Buttar D, Ting AKT, McGuire TM, et al. Discovery of clinical candidate AZD0095, a selective inhibitor of monocarboxylate transporter 4 (MCT4) for oncology. J Med Chem. 2023;66:384–97. 10.1021/acs.jmedchem.2c01342. 36525250 10.1021/acs.jmedchem.2c01342

[69] Benjamin D, Robay D, Hindupur SK, Pohlmann J, Colombi M, El-Shemerly MY, et al. Dual Inhibition of the lactate transporters MCT1 and MCT4 is synthetic lethal with Metformin due to NAD + Depletion in cancer cells. Cell Rep. 2018;25:3047–e30583044. 10.1016/j.celrep.2018.11.043. 30540938 10.1016/j.celrep.2018.11.043 PMC6302548

[70] He Y, Zhou C, Huang M, Tang C, Liu X, Yue Y, et al. Glyoxalase system: A systematic review of its biological activity, related-diseases, screening methods and small molecule regulators. Biomed Pharmacother. 2020;131:110663. 10.1016/j.biopha.2020.110663. 32858501 10.1016/j.biopha.2020.110663

[71] Rabbani N, Xue M, Weickert MO, Thornalley PJ. Multiple roles of glyoxalase 1-mediated suppression of Methylglyoxal glycation in cancer biology-Involvement in tumour suppression, tumour growth, multidrug resistance and target for chemotherapy. Semin Cancer Biol. 2018;49:83–93. 10.1016/j.semcancer.2017.05.006. 28506645 10.1016/j.semcancer.2017.05.006

[72] Bennis Y, Bodeau S, Batteux B, Gras-Champel V, Masmoudi K, Maizel J, et al. A study of associations between plasma Metformin Concentration, lactic Acidosis, and mortality in an emergency hospitalization context. Crit Care Med. 2020;48:e1194–202. 10.1097/ccm.0000000000004589. 33003077 10.1097/CCM.0000000000004589

[73] Gaffney DO, Jennings EQ, Anderson CC, Marentette JO, Shi T, Schou Oxvig AM, et al. Non-enzymatic lysine lactoylation of glycolytic enzymes. Cell Chem Biol. 2020;27:206–e213206. 10.1016/j.chembiol.2019.11.005. 31767537 10.1016/j.chembiol.2019.11.005 PMC7395678

[74] Mao Y, Zhang J, Zhou Q, He X, Zheng Z, Wei Y, et al. Hypoxia induces mitochondrial protein lactylation to limit oxidative phosphorylation. Cell Res. 2024;34:13–30. 10.1038/s41422-023-00864-6. 38163844 10.1038/s41422-023-00864-6 PMC10770133

[75] Allis CD, Jenuwein T. The molecular hallmarks of epigenetic control. Nat Rev Genet. 2016;17:487–500. 10.1038/nrg.2016.59. 27346641 10.1038/nrg.2016.59

[76] Yu J, Chai P, Xie M, Ge S, Ruan J, Fan X, et al. Histone lactylation drives oncogenesis by facilitating m(6)A reader protein YTHDF2 expression in ocular melanoma. Genome Biol. 2021;22:85. 10.1186/s13059-021-02308-z. 33726814 10.1186/s13059-021-02308-z PMC7962360

[77] Yang Z, Yan C, Ma J, Peng P, Ren X, Cai S, et al. Lactylome analysis suggests lactylation-dependent mechanisms of metabolic adaptation in hepatocellular carcinoma. Nat Metab. 2023;5:61–79. 10.1038/s42255-022-00710-w. 36593272 10.1038/s42255-022-00710-w

[78] Yang L, Niu K, Wang J, Shen W, Jiang R, Liu L, et al. Nucleolin lactylation contributes to intrahepatic cholangiocarcinoma pathogenesis via RNA splicing regulation of MADD. J Hepatol. 2024;81:651–66. 10.1016/j.jhep.2024.04.010. 38679071 10.1016/j.jhep.2024.04.010

[79] Wang X, Fan W, Li N, Ma Y, Yao M, Wang G, et al. YY1 lactylation in microglia promotes angiogenesis through transcription activation-mediated upregulation of FGF2. Genome Biol. 2023;24:87. 10.1186/s13059-023-02931-y. 37085894 10.1186/s13059-023-02931-y PMC10120156

[80] Yang K, Fan M, Wang X, Xu J, Wang Y, Tu F, et al. Lactate promotes macrophage HMGB1 lactylation, acetylation, and Exosomal release in polymicrobial sepsis. Cell Death Differ. 2022;29:133–46. 10.1038/s41418-021-00841-9. 34363018 10.1038/s41418-021-00841-9 PMC8738735

[81] Cui H, Xie N, Banerjee S, Ge J, Jiang D, Dey T, et al. Lung myofibroblasts promote macrophage profibrotic activity through Lactate-induced histone lactylation. Am J Respir Cell Mol Biol. 2021;64:115–25. 10.1165/rcmb.2020-0360OC. 33074715 10.1165/rcmb.2020-0360OC PMC7780997

[82] Dai W, Wu G, Liu K, Chen Q, Tao J, Liu H, et al. Lactate promotes myogenesis via activating H3K9 lactylation-dependent up-regulation of Neu2 expression. J Cachexia Sarcopenia Muscle. 2023;14:2851–65. 10.1002/jcsm.13363. 37919243 10.1002/jcsm.13363 PMC10751423

[83] Moreno-Yruela C, Zhang D, Wei W, Bæk M, Liu W, Gao J, et al. Class I histone deacetylases (HDAC1-3) are histone lysine delactylases. Sci Adv. 2022;8:eabi6696. 10.1126/sciadv.abi6696. 35044827 10.1126/sciadv.abi6696 PMC8769552

[84] Chu X, Di C, Chang P, Li L, Feng Z, Xiao S, et al. Lactylated histone H3K18 as a potential biomarker for the diagnosis and predicting the severity of septic shock. Front Immunol. 2021;12:786666. 10.3389/fimmu.2021.786666. 35069560 10.3389/fimmu.2021.786666 PMC8773995

[85] Yang W, Wang P, Cao P, Wang S, Yang Y, Su H, et al. Hypoxic in vitro culture reduces histone lactylation and impairs pre-implantation embryonic development in mice. Epigenetics Chromatin. 2021;14:57. 10.1186/s13072-021-00431-6. 34930415 10.1186/s13072-021-00431-6 PMC8691063

[86] Tian Q, Zhou LQ. Lactate activates germline and cleavage embryo genes in mouse embryonic stem cells. Cells. 2022;11. 10.3390/cells11030548. 10.3390/cells11030548 PMC8833948 35159357

[87] Yang Q, Liu J, Wang Y, Zhao W, Wang W, Cui J, et al. A proteomic atlas of ligand-receptor interactions at the ovine maternal-fetal interface reveals the role of histone lactylation in uterine remodeling. J Biol Chem. 2022;298:101456. 10.1016/j.jbc.2021.101456. 34861240 10.1016/j.jbc.2021.101456 PMC8733267

[88] Nian F, Qian Y, Xu F, Yang M, Wang H, Zhang Z. LDHA promotes osteoblast differentiation through histone lactylation. Biochem Biophys Res Commun. 2022;615:31–5. 10.1016/j.bbrc.2022.05.028. 35605402 10.1016/j.bbrc.2022.05.028

[89] Zhang X, Liu Y, Wang N. Multifaceted Roles of Histone Lysine Lactylation in Meiotic Gene Dynamics and Recombination. *bioRxiv* 2024. 10.1101/2024.01.25.576681

[90] Sun T, Zhang JN, Lan T, Shi L, Hu L, Yan L, et al. H3K14 lactylation exacerbates neuronal ferroptosis by inhibiting calcium efflux following intracerebral hemorrhagic stroke. Cell Death Dis. 2025;16:553. 10.1038/s41419-025-07874-9. 40701963 10.1038/s41419-025-07874-9 PMC12287320

[91] Wang R, Li C, Cheng Z, Li M, Shi J, Zhang Z, et al. H3K9 lactylation in malignant cells facilitates CD8(+) T cell dysfunction and poor immunotherapy response. Cell Rep. 2024;43:114957. 10.1016/j.celrep.2024.114957. 39475506 10.1016/j.celrep.2024.114957

[92] Wan N, Wang N, Yu S, Zhang H, Tang S, Wang D, et al. Cyclic immonium ion of lactyllysine reveals widespread lactylation in the human proteome. Nat Methods. 2022;19:854–64. 10.1038/s41592-022-01523-1. 35761067 10.1038/s41592-022-01523-1

[93] Luo Y, Yang Z, Yu Y, Zhang P. HIF1α lactylation enhances KIAA1199 transcription to promote angiogenesis and vasculogenic mimicry in prostate cancer. Int J Biol Macromol. 2022;222:2225–43. 10.1016/j.ijbiomac.2022.10.014. 36209908 10.1016/j.ijbiomac.2022.10.014

[94] Moreno-Yruela C, Bæk M, Monda F, Olsen CA. Chiral posttranslational modification to lysine ε-Amino groups. Acc Chem Res. 2022;55:1456–66. 10.1021/acs.accounts.2c00115. 35500056 10.1021/acs.accounts.2c00115

[95] Chen P, Zuo H, Xiong H, Kolar MJ, Chu Q, Saghatelian A, et al. Gpr132 sensing of lactate mediates tumor-macrophage interplay to promote breast cancer metastasis. Proc Natl Acad Sci U S A. 2017;114:580–5. 10.1073/pnas.1614035114. 28049847 10.1073/pnas.1614035114 PMC5255630

[96] Yang Z, Zheng Y, Gao Q. Lysine lactylation in the regulation of tumor biology. Trends Endocrinol Metab. 2024;35:720–31. 10.1016/j.tem.2024.01.011. 38395657 10.1016/j.tem.2024.01.011

[97] Ippolito L, Comito G, Parri M, Iozzo M, Duatti A, Virgilio F, et al. Lactate rewires lipid metabolism and sustains a Metabolic-Epigenetic axis in prostate cancer. Cancer Res. 2022;82:1267–82. 10.1158/0008-5472.Can-21-0914. 35135811 10.1158/0008-5472.CAN-21-0914 PMC7612586

[98] Lu J, Luo Y, Rao D, Wang T, Lei Z, Chen X, et al. Myeloid-derived suppressor cells in cancer: therapeutic targets to overcome tumor immune evasion. Exp Hematol Oncol. 2024;13:39. 10.1186/s40164-024-00505-7. 38609997 10.1186/s40164-024-00505-7 PMC11010322

[99] Certo M, Tsai CH, Pucino V, Ho PC, Mauro C. Lactate modulation of immune responses in inflammatory versus tumour microenvironments. Nat Rev Immunol. 2021;21:151–61. 10.1038/s41577-020-0406-2. 32839570 10.1038/s41577-020-0406-2

[100] Yang X, Lu Y, Hang J, Zhang J, Zhang T, Huo Y, et al. Lactate-Modulated immunosuppression of Myeloid-Derived suppressor cells contributes to the radioresistance of pancreatic cancer. Cancer Immunol Res. 2020;8:1440–51. 10.1158/2326-6066.Cir-20-0111. 32917658 10.1158/2326-6066.CIR-20-0111

[101] Zeng Y, Huang Y, Tan Q, Peng L, Wang J, Tong F, et al. Influence of lactate in resistance to anti–PD–1/PD–L1 therapy: mechanisms and clinical applications (Review). Mol Med Rep. 2025;31. 10.3892/mmr.2024.13413. 10.3892/mmr.2024.13413 PMC11650113 39670310

[102] Qian Y, Galan-Cobo A, Guijarro I, Dang M, Molkentine D, Poteete A, et al. MCT4-dependent lactate secretion suppresses antitumor immunity in LKB1-deficient lung adenocarcinoma. Cancer Cell. 2023;41:1363–e13801367. 10.1016/j.ccell.2023.05.015. 37327788 10.1016/j.ccell.2023.05.015 PMC11161201

[103] Zhang J, Muri J, Fitzgerald G, Gorski T, Gianni-Barrera R, Masschelein E, et al. Endothelial lactate controls muscle regeneration from ischemia by inducing M2-like macrophage polarization. Cell Metab. 2020;31:1136–e11531137. 10.1016/j.cmet.2020.05.004. 32492393 10.1016/j.cmet.2020.05.004 PMC7267778

[104] Selleri S, Bifsha P, Civini S, Pacelli C, Dieng MM, Lemieux W, et al. Human mesenchymal stromal cell-secreted lactate induces M2-macrophage differentiation by metabolic reprogramming. Oncotarget. 2016;7:30193–210. 10.18632/oncotarget.8623. 27070086 10.18632/oncotarget.8623 PMC5058674

[105] Liu Y, Xu R, Gu H, Zhang E, Qu J, Cao W, et al. Metabolic reprogramming in macrophage responses. Biomark Res. 2021;9:1. 10.1186/s40364-020-00251-y. 33407885 10.1186/s40364-020-00251-y PMC7786975

[106] Zhang W, Wang G, Xu ZG, Tu H, Hu F, Dai J, et al. Lactate is a natural suppressor of RLR signaling by targeting MAVS. Cell. 2019;178:176–e189115. 10.1016/j.cell.2019.05.003. 31155231 10.1016/j.cell.2019.05.003 PMC6625351

[107] Colegio OR, Chu NQ, Szabo AL, Chu T, Rhebergen AM, Jairam V, et al. Functional polarization of tumour-associated macrophages by tumour-derived lactic acid. Nature. 2014;513:559–63. 10.1038/nature13490. 25043024 10.1038/nature13490 PMC4301845

[108] Xu B, Liu Y, Li N, Geng Q. Lactate and lactylation in macrophage metabolic reprogramming: current progress and outstanding issues. Front Immunol. 2024;15:1395786. 10.3389/fimmu.2024.1395786. 38835758 10.3389/fimmu.2024.1395786 PMC11148263

[109] Zanna MY, Yasmin AR, Omar AR, Arshad SS, Mariatulqabtiah AR, Nur-Fazila SH, et al. Review of dendritic Cells, their role in clinical Immunology, and distribution in various animal species. Int J Mol Sci. 2021;22. 10.3390/ijms22158044. 10.3390/ijms22158044 PMC8348663 34360810

[110] Nasi A, Fekete T, Krishnamurthy A, Snowden S, Rajnavölgyi E, Catrina AI, et al. Dendritic cell reprogramming by endogenously produced lactic acid. J Immunol. 2013;191:3090–9. 10.4049/jimmunol.1300772. 23956421 10.4049/jimmunol.1300772

[111] Bischoff SC. Role of mast cells in allergic and non-allergic immune responses: comparison of human and murine data. Nat Rev Immunol. 2007;7:93–104. 10.1038/nri2018. 17259966 10.1038/nri2018

[112] Abebayehu D, Spence AJ, Caslin H, Taruselli M, Haque TT, Kiwanuka KN, et al. Lactic acid suppresses IgE-mediated mast cell function in vitro and in vivo. Cell Immunol. 2019;341:103918. 10.1016/j.cellimm.2019.04.006. 31030957 10.1016/j.cellimm.2019.04.006 PMC6579658

[113] Syed M, Kammala AK, Callahan B, Oskeritzian CA, Subramanian H. Lactic acid suppresses MRGPRX2 mediated mast cell responses. Cell Immunol. 2021;368:104422. 10.1016/j.cellimm.2021.104422. 34399172 10.1016/j.cellimm.2021.104422 PMC8428143

[114] Abebayehu D, Spence AJ, Qayum AA, Taruselli MT, McLeod JJ, Caslin HL, et al. Lactic acid suppresses IL-33-Mediated mast cell inflammatory responses via Hypoxia-Inducible Factor-1α-Dependent miR-155 suppression. J Immunol. 2016;197:2909–17. 10.4049/jimmunol.1600651. 27559047 10.4049/jimmunol.1600651 PMC5026940

[115] Sun L, Su Y, Jiao A, Wang X, Zhang B. T cells in health and disease. Signal Transduct Target Ther. 2023;8:235. 10.1038/s41392-023-01471-y. 37332039 10.1038/s41392-023-01471-y PMC10277291

[116] Haas R, Smith J, Rocher-Ros V, Nadkarni S, Montero-Melendez T, D’Acquisto F, et al. Lactate regulates metabolic and Pro-inflammatory circuits in control of T cell migration and effector functions. PLoS Biol. 2015;13:e1002202. 10.1371/journal.pbio.1002202. 26181372 10.1371/journal.pbio.1002202 PMC4504715

[117] Wen J, Cheng S, Zhang Y, Wang R, Xu J, Ling Z, et al. Lactate anions participate in T cell cytokine production and function. Sci China Life Sci. 2021;64:1895–905. 10.1007/s11427-020-1887-7. 33580429 10.1007/s11427-020-1887-7

[118] Watson MJ, Vignali PDA, Mullett SJ, Overacre-Delgoffe AE, Peralta RM, Grebinoski S, et al. Metabolic support of tumour-infiltrating regulatory T cells by lactic acid. Nature. 2021;591:645–51. 10.1038/s41586-020-03045-2. 33589820 10.1038/s41586-020-03045-2 PMC7990682

[119] Sun R, Chi H. Metabolic-epigenetic rewiring in CD8(+) T cells via lactate-dependent histone lactylation. Nat Immunol. 2024;25:1980–2. 10.1038/s41590-024-01991-x. 39415052 10.1038/s41590-024-01991-x

[120] Fischbeck AJ, Ruehland S, Ettinger A, Paetzold K, Masouris I, Noessner E, et al. Tumor lactic acidosis: protecting tumor by inhibiting cytotoxic activity through motility arrest and bioenergetic Silencing. Front Oncol. 2020;10:589434. 10.3389/fonc.2020.589434. 33364193 10.3389/fonc.2020.589434 PMC7753121

[121] Peng M, Yin N, Chhangawala S, Xu K, Leslie CS, Li MO. Aerobic Glycolysis promotes T helper 1 cell differentiation through an epigenetic mechanism. Science. 2016;354:481–4. 10.1126/science.aaf6284. 27708054 10.1126/science.aaf6284 PMC5539971

[122] Fujii W, Kawahito Y, Nagahara H, Kukida Y, Seno T, Yamamoto A, et al. Monocarboxylate transporter 4, associated with the acidification of synovial fluid, is a novel therapeutic target for inflammatory arthritis. Arthritis Rheumatol. 2015;67:2888–96. 10.1002/art.39270. 26213210 10.1002/art.39270

[123] Pucino V, Certo M, Bulusu V, Cucchi D, Goldmann K, Pontarini E, et al. Lactate buildup at the site of chronic inflammation promotes disease by inducing CD4(+) T cell metabolic rewiring. Cell Metab. 2019;30:1055–e10741058. 10.1016/j.cmet.2019.10.004. 31708446 10.1016/j.cmet.2019.10.004 PMC6899510

[124] Kobayashi T, Siegmund B, Le Berre C, Wei SC, Ferrante M, Shen B, et al. Ulcerative colitis. Nat Rev Dis Primers. 2020;6:74. 10.1038/s41572-020-0205-x. 32913180 10.1038/s41572-020-0205-x

[125] Herrador-López M, Martín-Masot R, Navas-López VM. Dietary interventions in ulcerative colitis: A systematic review of the evidence with Meta-Analysis. Nutrients. 2023;15. 10.3390/nu15194194. 10.3390/nu15194194 PMC10574654 37836478

[126] O’Callaghan A, van Sinderen D. Bifidobacteria and their role as members of the human gut microbiota. Front Microbiol. 2016;7:925. 10.3389/fmicb.2016.00925. 27379055 10.3389/fmicb.2016.00925 PMC4908950

[127] Ranganathan P, Shanmugam A, Swafford D, Suryawanshi A, Bhattacharjee P, Hussein MS, et al. GPR81, a Cell-Surface receptor for Lactate, regulates intestinal homeostasis and protects mice from experimental colitis. J Immunol. 2018;200:1781–9. 10.4049/jimmunol.1700604. 29386257 10.4049/jimmunol.1700604 PMC5858928

[128] Iraporda C, Romanin DE, Bengoa AA, Errea AJ, Cayet D, Foligné B, et al. Local treatment with lactate prevents intestinal inflammation in the TNBS-Induced colitis model. Front Immunol. 2016;7:651. 10.3389/fimmu.2016.00651. 28082985 10.3389/fimmu.2016.00651 PMC5187354

[129] Sun S, Xu X, Liang L, Wang X, Bai X, Zhu L, et al. Lactic Acid-Producing probiotic Saccharomyces cerevisiae attenuates ulcerative colitis via suppressing macrophage pyroptosis and modulating gut microbiota. Front Immunol. 2021;12:777665. 10.3389/fimmu.2021.777665. 34899735 10.3389/fimmu.2021.777665 PMC8652295

[130] Franzosa EA, Sirota-Madi A, Avila-Pacheco J, Fornelos N, Haiser HJ, Reinker S, et al. Gut Microbiome structure and metabolic activity in inflammatory bowel disease. Nat Microbiol. 2019;4:293–305. 10.1038/s41564-018-0306-4. 30531976 10.1038/s41564-018-0306-4 PMC6342642

[131] Irvin C, Zafar I, Good J, Rollins D, Christianson C, Gorska MM, et al. Increased frequency of dual-positive TH2/TH17 cells in Bronchoalveolar lavage fluid characterizes a population of patients with severe asthma. J Allergy Clin Immunol. 2014;134:1175–e11861177. 10.1016/j.jaci.2014.05.038. 25042748 10.1016/j.jaci.2014.05.038 PMC4254017

[132] Vermeulen RP, Hoekstra M, Nijsten MW, van der Horst IC, van Pelt LJ, Jessurun GA, et al. Clinical correlates of arterial lactate levels in patients with ST-segment elevation myocardial infarction at admission: a descriptive study. Crit Care. 2010;14:R164. 10.1186/cc9253. 20825687 10.1186/cc9253 PMC3219257

[133] Fan M, Yang K, Wang X, Chen L, Gill PS, Ha T, et al. Lactate promotes endothelial-to-mesenchymal transition via Snail1 lactylation after myocardial infarction. Sci Adv. 2023;9:eadc9465. 10.1126/sciadv.adc9465. 36735787 10.1126/sciadv.adc9465 PMC9897666

[134] Gilsbach R, Schwaderer M, Preissl S, Grüning BA, Kranzhöfer D, Schneider P, et al. Distinct epigenetic programs regulate cardiac myocyte development and disease in the human heart in vivo. Nat Commun. 2018;9:391. 10.1038/s41467-017-02762-z. 29374152 10.1038/s41467-017-02762-z PMC5786002

[135] Zhang N, Zhang Y, Xu J, Wang P, Wu B, Lu S, et al. α-myosin heavy chain lactylation maintains sarcomeric structure and function and alleviates the development of heart failure. Cell Res. 2023;33:679–98. 10.1038/s41422-023-00844-w. 37443257 10.1038/s41422-023-00844-w PMC10474270

[136] Fillmore N, Levasseur JL, Fukushima A, Wagg CS, Wang W, Dyck JRB, et al. Uncoupling of Glycolysis from glucose oxidation accompanies the development of heart failure with preserved ejection fraction. Mol Med. 2018;24:3. 10.1186/s10020-018-0005-x. 30134787 10.1186/s10020-018-0005-x PMC6016884

[137] Cluntun AA, Badolia R, Lettlova S, Parnell KM, Shankar TS, Diakos NA, et al. The pyruvate-lactate axis modulates cardiac hypertrophy and heart failure. Cell Metab. 2021;33:629–e648610. 10.1016/j.cmet.2020.12.003. 33333007 10.1016/j.cmet.2020.12.003 PMC7933116

[138] Ma XM, Geng K, Wang P, Jiang Z, Law BY, Xu Y. MCT4-dependent lactate transport: a novel mechanism for cardiac energy metabolism injury and inflammation in type 2 diabetes mellitus. Cardiovasc Diabetol. 2024;23:96. 10.1186/s12933-024-02178-2. 38486199 10.1186/s12933-024-02178-2 PMC10941417

[139] Nalos M, Leverve X, Huang S, Weisbrodt L, Parkin R, Seppelt I, et al. Half-molar sodium lactate infusion improves cardiac performance in acute heart failure: a pilot randomised controlled clinical trial. Crit Care. 2014;18:R48. 10.1186/cc13793. 24666826 10.1186/cc13793 PMC4057379

[140] Johannsson E, Lunde PK, Heddle C, Sjaastad I, Thomas MJ, Bergersen LH, et al. Upregulation of the cardiac monocarboxylate transporter MCT1 in a rat model of congestive heart failure. Circulation: J Am Heart Association. 2001;104:729–34. 10.1161/hc3201.092286 11489783

[141] Jin ES, Sherry AD, Malloy CR. Lactate contributes to glyceroneogenesis and glyconeogenesis in skeletal muscle by reversal of pyruvate kinase. J Biol Chem. 2015;290:30486–97. 10.1074/jbc.M115.689174. 26491014 10.1074/jbc.M115.689174 PMC4683270

[142] San-Millán I, Brooks GA. Assessment of metabolic flexibility by means of measuring blood Lactate, Fat, and carbohydrate oxidation responses to exercise in professional endurance athletes and Less-Fit individuals. Sports Med. 2018;48:467–79. 10.1007/s40279-017-0751-x. 28623613 10.1007/s40279-017-0751-x

[143] Cardia RDE-C, Vieira-Gadducci L, Greve A, Santo JM, LACTATE CAN BE A MA, MARKER OF METABOLIC SYNDROME IN SEVERE OBESITY?. Arq Bras Cir Dig. 2021;34:e1579. 10.1590/0102-672020210001e1579. 34133526 10.1590/0102-672020210001e1579 PMC8195466

[144] Lin Y, Bai M, Wang S, Chen L, Li Z, Li C, et al. Lactate is a key mediator that links obesity to insulin resistance via modulating cytokine production from adipose tissue. Diabetes. 2022;71:637–52. 10.2337/db21-0535. 35044451 10.2337/db21-0535

[145] Feng T, Zhao X, Gu P, Yang W, Wang C, Guo Q, et al. Adipocyte-derived lactate is a signalling metabolite that potentiates adipose macrophage inflammation via targeting PHD2. Nat Commun. 2022;13:5208. 10.1038/s41467-022-32871-3. 36064857 10.1038/s41467-022-32871-3 PMC9445001

[146] Cai H, Wang X, Zhang Z, Chen J, Wang F, Wang L, et al. Moderate l-lactate administration suppresses adipose tissue macrophage M1 polarization to alleviate obesity-associated insulin resistance. J Biol Chem. 2022;298:101768. 10.1016/j.jbc.2022.101768. 35218776 10.1016/j.jbc.2022.101768 PMC8941214

[147] Yao Z, Yan Y, Zheng X, Wang M, Zhang H, Li H, et al. Dietary lactate supplementation protects against obesity by promoting adipose Browning in mice. J Agric Food Chem. 2020;68:14841–9. 10.1021/acs.jafc.0c05899. 33284607 10.1021/acs.jafc.0c05899

[148] Hargreaves M, Spriet LL. Skeletal muscle energy metabolism during exercise. Nat Metab. 2020;2:817–28. 10.1038/s42255-020-0251-4. 32747792 10.1038/s42255-020-0251-4

[149] Liu C, Wu J, Zhu J, Kuei C, Yu J, Shelton J, et al. Lactate inhibits lipolysis in fat cells through activation of an orphan G-protein-coupled receptor, GPR81. J Biol Chem. 2009;284:2811–22. 10.1074/jbc.M806409200. 19047060 10.1074/jbc.M806409200

[150] Qu Y, Chen S, Zhou L, Chen M, Li L, Ni Y, et al. The different effects of intramuscularly-injected lactate on white and brown adipose tissue in vivo. Mol Biol Rep. 2022;49:8507–16. 10.1007/s11033-022-07672-y. 35753026 10.1007/s11033-022-07672-y

[151] Chang SH, Jang J, Oh S, Yoon JH, Jo DG, Yun UJ, et al. Nrf2 induces Ucp1 expression in adipocytes in response to β3-AR stimulation and enhances oxygen consumption in high-fat diet-fed obese mice. BMB Rep. 2021;54:419–24. 10.5483/BMBRep.2021.54.8.023. 33691909 10.5483/BMBRep.2021.54.8.023 PMC8411042

[152] Evans L, Rhodes A, Alhazzani W, Antonelli M, Coopersmith CM, French C, et al. Surviving sepsis campaign: international guidelines for management of sepsis and septic shock 2021. Crit Care Med. 2021;49:e1063–143. 10.1097/ccm.0000000000005337. 34605781 10.1097/CCM.0000000000005337

[153] Hayashi Y, Endoh H, Kamimura N, Tamakawa T, Nitta M. Lactate indices as predictors of in-hospital mortality or 90-day survival after admission to an intensive care unit in unselected critically ill patients. PLoS ONE. 2020;15:e0229135. 10.1371/journal.pone.0229135. 32150560 10.1371/journal.pone.0229135 PMC7062275

[154] Sauer CM, Gómez J, Botella MR, Ziehr DR, Oldham WM, Gavidia G, et al. Understanding critically ill sepsis patients with normal serum lactate levels: results from U.S. And European ICU cohorts. Sci Rep. 2021;11:20076. 10.1038/s41598-021-99581-6. 34625640 10.1038/s41598-021-99581-6 PMC8501011

[155] Loftus RM, Finlay DK. Immunometabolism: cellular metabolism turns immune regulator. J Biol Chem. 2016;291:1–10. 10.1074/jbc.R115.693903. 26534957 10.1074/jbc.R115.693903 PMC4697146

[156] Caslin HL, Abebayehu D, Abdul Qayum A, Haque TT, Taruselli MT, Paez PA, et al. Lactic acid inhibits Lipopolysaccharide-Induced mast cell function by limiting Glycolysis and ATP availability. J Immunol. 2019;203:453–64. 10.4049/jimmunol.1801005. 31160535 10.4049/jimmunol.1801005 PMC6734564

[157] Luo Y, Li L, Chen X, Gou H, Yan K, Xu Y. Effects of lactate in immunosuppression and inflammation: progress and prospects. Int Rev Immunol. 2022;41:19–29. 10.1080/08830185.2021.1974856. 34486916 10.1080/08830185.2021.1974856

[158] Nolt B, Tu F, Wang X, Ha T, Winter R, Williams DL et al. Lactate and Immunosuppression in Sepsis. *Shock* 2018; 49: 120–125. 10.1097/shk.0000000000000958 10.1097/SHK.0000000000000958 PMC5757670 28767543

[159] Hoque R, Farooq A, Ghani A, Gorelick F, Mehal WZ. Lactate reduces liver and pancreatic injury in Toll-like receptor- and inflammasome-mediated inflammation via GPR81-mediated suppression of innate immunity. Gastroenterology. 2014;146:1763–74. 10.1053/j.gastro.2014.03.014. 24657625 10.1053/j.gastro.2014.03.014 PMC4104305

[160] Errea A, Cayet D, Marchetti P, Tang C, Kluza J, Offermanns S, et al. Lactate inhibits the Pro-Inflammatory response and metabolic reprogramming in murine macrophages in a GPR81-Independent manner. PLoS ONE. 2016;11:e0163694. 10.1371/journal.pone.0163694. 27846210 10.1371/journal.pone.0163694 PMC5112849

[161] Khatib-Massalha E, Bhattacharya S, Massalha H, Biram A, Golan K, Kollet O, et al. Lactate released by inflammatory bone marrow neutrophils induces their mobilization via endothelial GPR81 signaling. Nat Commun. 2020;11:3547. 10.1038/s41467-020-17402-2. 32669546 10.1038/s41467-020-17402-2 PMC7363928

[162] Wilkinson HN, Hardman MJ. Wound healing: cellular mechanisms and pathological outcomes. Open Biol. 2020;10:200223. 10.1098/rsob.200223. 32993416 10.1098/rsob.200223 PMC7536089

[163] Bacci S. Cellular mechanisms and therapies in wound healing: looking toward the future. Biomedicines. 2021;9. 10.3390/biomedicines9111611. 10.3390/biomedicines9111611 PMC8615875 34829840

[164] Tottoli EM, Dorati R, Genta I, Chiesa E, Pisani S, Conti B. Skin Wound Healing Process and New Emerging Technologies for Skin Wound Care and Regeneration. *Pharmaceutics* 2020; 12. 10.3390/pharmaceutics12080735 10.3390/pharmaceutics12080735 PMC7463929 32764269

[165] Amiri N, Golin AP, Jalili RB, Ghahary A. Roles of cutaneous cell-cell communication in wound healing outcome: an emphasis on keratinocyte-fibroblast crosstalk. Exp Dermatol. 2022;31:475–84. 10.1111/exd.14516. 34932841 10.1111/exd.14516

[166] Porporato PE, Payen VL, De Saedeleer CJ, Préat V, Thissen JP, Feron O, et al. Lactate stimulates angiogenesis and accelerates the healing of superficial and ischemic wounds in mice. Angiogenesis. 2012;15:581–92. 10.1007/s10456-012-9282-0. 22660894 10.1007/s10456-012-9282-0

[167] Hunt TK, Aslam R, Hussain Z, Beckert S. Lactate, with oxygen, incites angiogenesis. Adv Exp Med Biol. 2008;614:73–80. 10.1007/978-0-387-74911-2_9. 18290316 10.1007/978-0-387-74911-2_9

[168] Hait NC, Maiti A, Xu P, Qi Q, Kawaguchi T, Okano M, et al. Regulation of hypoxia-inducible factor functions in the nucleus by sphingosine-1-phosphate. Faseb J. 2020;34:4293–310. 10.1096/fj.201901734RR. 32017264 10.1096/fj.201901734RR PMC10112293

[169] Wu H, Chu Y, Sun S, Li G, Xu S, Zhang X, et al. Hypoxia-Mediated complement 1q binding protein regulates metastasis and chemoresistance in Triple-Negative breast cancer and modulates the PKC-NF-κB-VCAM-1 signaling pathway. Front Cell Dev Biol. 2021;9:607142. 10.3389/fcell.2021.607142. 33708767 10.3389/fcell.2021.607142 PMC7940382

[170] Milovanova TN, Bhopale VM, Sorokina EM, Moore JS, Hunt TK, Hauer-Jensen M, et al. Lactate stimulates vasculogenic stem cells via the thioredoxin system and engages an autocrine activation loop involving hypoxia-inducible factor 1. Mol Cell Biol. 2008;28:6248–61. 10.1128/mcb.00795-08. 18710947 10.1128/MCB.00795-08 PMC2577432

[171] Hunt TK, Conolly WB, Aronson SB, Goldstein P. Anaerobic metabolism and wound healing: an hypothesis for the initiation and cessation of collagen synthesis in wounds. Am J Surg. 1978;135:328–32. 10.1016/0002-9610(78)90061-2. 626315 10.1016/0002-9610(78)90061-2

[172] Weng HP, Cheng YY, Lee HL, Hsu TY, Chang YT, Shen YA. Enhanced Platelet-Rich plasma (ePRP) stimulates wound healing through effects on metabolic reprogramming in fibroblasts. Int J Mol Sci. 2021;22. 10.3390/ijms222312623. 10.3390/ijms222312623 PMC8657780 34884429

[173] Irizarry-Caro RA, McDaniel MM, Overcast GR, Jain VG, Troutman TD, Pasare C. TLR signaling adapter BCAP regulates inflammatory to reparatory macrophage transition by promoting histone lactylation. Proc Natl Acad Sci U S A. 2020;117:30628–38. 10.1073/pnas.2009778117. 33199625 10.1073/pnas.2009778117 PMC7720107

[174] Wu H, Liang W, Han M, Zhen Y, Chen L, Li H, et al. Mechanisms regulating wound healing: functional changes in biology mediated by lactate and histone lactylation. J Cell Physiol. 2023;238:2243–52. 10.1002/jcp.31122. 37743554 10.1002/jcp.31122

[175] Xie H, Hou S, Jiang J, Sekutowicz M, Kelly J, Bacskai BJ. Rapid cell death is preceded by amyloid plaque-mediated oxidative stress. Proc Natl Acad Sci U S A. 2013;110:7904–9. 10.1073/pnas.1217938110. 23610434 10.1073/pnas.1217938110 PMC3651444

[176] Sun N, Youle RJ, Finkel T. The mitochondrial basis of aging. Mol Cell. 2016;61:654–66. 10.1016/j.molcel.2016.01.028. 26942670 10.1016/j.molcel.2016.01.028 PMC4779179

[177] Liguori C, Chiaravalloti A, Sancesario G, Stefani A, Sancesario G, Mercuri NB, et al. Cerebrospinal fluid lactate levels and brain [18F]FDG PET hypometabolism within the default mode network in alzheimer’s disease. Eur J Nucl Med Mol Imaging. 2016;43:2040–9. 27221635 10.1007/s00259-016-3417-2

[178] Tauffenberger A, Fiumelli H, Almustafa S, Magistretti PJ. Lactate and pyruvate promote oxidative stress resistance through hormetic ROS signaling. Cell Death Dis. 2019;10:653. 10.1038/s41419-019-1877-6. 31506428 10.1038/s41419-019-1877-6 PMC6737085

[179] Jia L, Liao M, Mou A, Zheng Q, Yang W, Yu Z, et al. Rheb-regulated mitochondrial pyruvate metabolism of Schwann cells linked to axon stability. Dev Cell. 2021;56:2980–e29942986. 10.1016/j.devcel.2021.09.013. 34619097 10.1016/j.devcel.2021.09.013

[180] Yang L, Gilbertsen A, Xia H, Benyumov A, Smith K, Herrera J, et al. Hypoxia enhances IPF mesenchymal progenitor cell fibrogenicity via the lactate/GPR81/HIF1α pathway. JCI Insight. 2023;8. 10.1172/jci.insight.163820. 10.1172/jci.insight.163820 PMC9977506 36656644

[181] Sun Z, Ji Z, Meng H, He W, Li B, Pan X, et al. Lactate facilitated mitochondrial fission-derived ROS to promote pulmonary fibrosis via ERK/DRP-1 signaling. J Transl Med. 2024;22:479. 10.1186/s12967-024-05289-2. 38773615 10.1186/s12967-024-05289-2 PMC11106888

[182] Magistretti PJ, Allaman I. A cellular perspective on brain energy metabolism and functional imaging. Neuron. 2015;86:883–901. 10.1016/j.neuron.2015.03.035. 25996133 10.1016/j.neuron.2015.03.035

[183] Magistretti PJ, Allaman I. Lactate in the brain: from metabolic end-product to signalling molecule. Nat Rev Neurosci. 2018;19:235–49. 10.1038/nrn.2018.19. 29515192 10.1038/nrn.2018.19

[184] Chaudhari P, Madaan A, Rivera JC, Charfi I, Habelrih T, Hou X, et al. Neuronal GPR81 regulates developmental brain angiogenesis and promotes brain recovery after a hypoxic ischemic insult. J Cereb Blood Flow Metab. 2022;42:1294–308. 10.1177/0271678x221077499. 35107038 10.1177/0271678X221077499 PMC9207492

[185] Bajaffer A, Mineta K, Magistretti P, Gojobori T. Lactate-mediated neural plasticity genes emerged during the evolution of memory systems. Sci Rep. 2022;12:19238. 10.1038/s41598-022-23784-8. 36357482 10.1038/s41598-022-23784-8 PMC9649800

[186] Morland C, Andersson KA, Haugen ØP, Hadzic A, Kleppa L, Gille A, et al. Exercise induces cerebral VEGF and angiogenesis via the lactate receptor HCAR1. Nat Commun. 2017;8:15557. 10.1038/ncomms15557. 28534495 10.1038/ncomms15557 PMC5457513

[187] Cerina M, Levers M, Keller JM, Frega M. Neuroprotective role of lactate in a human in vitro model of the ischemic penumbra. Sci Rep. 2024;14:7973. 10.1038/s41598-024-58669-5. 38575687 10.1038/s41598-024-58669-5 PMC10994928

[188] Zhao Z, Liu Y, Ji H. Association between serum lactate and mortality in critically ill ischemic stroke patients based on MIMIC-IV data. Sci Rep. 2025;15:26155. 10.1038/s41598-025-11461-5. 40681728 10.1038/s41598-025-11461-5 PMC12274637

[189] Zhang XD, Zhang ZY, Zhao MP, Zhang XT, Wang N, Gao HZ, et al. Lactate dehydrogenase to albumin ratio and poor prognosis after thrombolysis in ischemic stroke patients: developing a novel nomogram. BMC Med Inf Decis Mak. 2025;25:166. 10.1186/s12911-025-02991-z. 10.1186/s12911-025-02991-z PMC12001606 40234875

[190] Nilsson OG, Brandt L, Ungerstedt U, Säveland H. Bedside detection of brain ischemia using intracerebral microdialysis: subarachnoid hemorrhage and delayed ischemic deterioration. Neurosurgery. 1999;45:1176–84. 10.1097/00006123-199911000-00032. discussion 1184 – 1175. 10549935 10.1097/00006123-199911000-00032

[191] Chen X, Threlkeld SW, Cummings EE, Juan I, Makeyev O, Besio WG, et al. Ischemia-reperfusion impairs blood-brain barrier function and alters tight junction protein expression in the ovine fetus. Neuroscience. 2012;226:89–100. 10.1016/j.neuroscience.2012.08.043. 22986172 10.1016/j.neuroscience.2012.08.043 PMC3490041

[192] Hamann GF, Liebetrau M, Martens H, Burggraf D, Kloss CU, Bültemeier G, et al. Microvascular basal lamina injury after experimental focal cerebral ischemia and reperfusion in the rat. J Cereb Blood Flow Metab. 2002;22:526–33. 10.1097/00004647-200205000-00004. 11973425 10.1097/00004647-200205000-00004

[193] Pierre K, Pellerin L. Monocarboxylate transporters in the central nervous system: distribution, regulation and function. J Neurochem. 2005;94:1–14. 10.1111/j.1471-4159.2005.03168.x. 15953344 10.1111/j.1471-4159.2005.03168.x

[194] Roumes H, Jollé C, Blanc J, Benkhaled I, Chatain CP, Massot P, et al. Lactate transporters in the rat barrel cortex sustain whisker-dependent BOLD fMRI signal and behavioral performance. Proc Natl Acad Sci U S A. 2021;118. 10.1073/pnas.2112466118. 10.1073/pnas.2112466118 PMC8617497 34782470

[195] Cauli B, Dusart I, Li D. Lactate as a determinant of neuronal excitability, neuroenergetics and beyond. Neurobiol Dis. 2023;184:106207. 10.1016/j.nbd.2023.106207. 37331530 10.1016/j.nbd.2023.106207

[196] Norrmén C, Figlia G, Pfistner P, Pereira JA, Bachofner S, Suter U. mTORC1 is transiently reactivated in injured nerves to promote c-Jun elevation and Schwann cell dedifferentiation. J Neurosci. 2018;38:4811–28. 10.1523/jneurosci.3619-17.2018. 29695414 10.1523/JNEUROSCI.3619-17.2018 PMC5956991

[197] Babetto E, Wong KM, Beirowski B. A glycolytic shift in Schwann cells supports injured axons. Nat Neurosci. 2020;23:1215–28. 10.1038/s41593-020-0689-4. 32807950 10.1038/s41593-020-0689-4 PMC8758250

[198] Berthet C, Lei H, Thevenet J, Gruetter R, Magistretti PJ, Hirt L. Neuroprotective role of lactate after cerebral ischemia. J Cereb Blood Flow Metab. 2009;29:1780–9. 10.1038/jcbfm.2009.97. 19675565 10.1038/jcbfm.2009.97

[199] Horn T, Klein J. Neuroprotective effects of lactate in brain ischemia: dependence on anesthetic drugs. Neurochem Int. 2013;62:251–7. 10.1016/j.neuint.2012.12.017. 23298645 10.1016/j.neuint.2012.12.017

[200] Koyama T, Shibata M, Moritani K. Ischemic postconditioning with lactate-enriched blood in patients with acute myocardial infarction. Cardiology. 2013;125:92–3. 10.1159/000350595. 23711672 10.1159/000350595

[201] Zhang FL, Chen XW, Wang YF, Hu Z, Zhang WJ, Zhou BW, et al. Microbiota-derived Tryptophan metabolites indole-3-lactic acid is associated with intestinal ischemia/reperfusion injury via positive regulation of YAP and Nrf2. J Transl Med. 2023;21:264. 10.1186/s12967-023-04109-3. 37072757 10.1186/s12967-023-04109-3 PMC10111656

[202] Li L, Li L, Li W, Chen T, Bin Z, Zhao L, et al. TAp73-induced phosphofructokinase-1 transcription promotes the Warburg effect and enhances cell proliferation. Nat Commun. 2018;9:4683. 10.1038/s41467-018-07127-8. 30409970 10.1038/s41467-018-07127-8 PMC6224601

[203] Zhao Z, Wu MS, Zou C, Tang Q, Lu J, Liu D, et al. Downregulation of MCT1 inhibits tumor growth, metastasis and enhances chemotherapeutic efficacy in osteosarcoma through regulation of the NF-κB pathway. Cancer Lett. 2014;342:150–8. 10.1016/j.canlet.2013.08.042. 24012639 10.1016/j.canlet.2013.08.042

[204] Roland CL, Arumugam T, Deng D, Liu SH, Philip B, Gomez S, et al. Cell surface lactate receptor GPR81 is crucial for cancer cell survival. Cancer Res. 2014;74:5301–10. 10.1158/0008-5472.Can-14-0319. 24928781 10.1158/0008-5472.CAN-14-0319 PMC4167222

[205] Brand A, Singer K, Koehl GE, Kolitzus M, Schoenhammer G, Thiel A, et al. LDHA-Associated lactic acid production blunts tumor immunosurveillance by T and NK cells. Cell Metab. 2016;24:657–71. 10.1016/j.cmet.2016.08.011. 27641098 10.1016/j.cmet.2016.08.011

[206] Zhang Y, Zhang X, Meng Y, Xu X, Zuo D. The role of Glycolysis and lactate in the induction of tumor-associated macrophages immunosuppressive phenotype. Int Immunopharmacol. 2022;110:108994. 10.1016/j.intimp.2022.108994. 35777265 10.1016/j.intimp.2022.108994

[207] Fischer K, Hoffmann P, Voelkl S, Meidenbauer N, Ammer J, Edinger M, et al. Inhibitory effect of tumor cell-derived lactic acid on human T cells. Blood. 2007;109:3812–9. 10.1182/blood-2006-07-035972. 17255361 10.1182/blood-2006-07-035972

[208] Xiong J, He J, Zhu J, Pan J, Liao W, Ye H, et al. Lactylation-driven METTL3-mediated RNA m(6)A modification promotes immunosuppression of tumor-infiltrating myeloid cells. Mol Cell. 2022;82:1660–e16771610. 10.1016/j.molcel.2022.02.033. 35320754 10.1016/j.molcel.2022.02.033

[209] Feng J, Yang H, Zhang Y, Wei H, Zhu Z, Zhu B, et al. Tumor cell-derived lactate induces TAZ-dependent upregulation of PD-L1 through GPR81 in human lung cancer cells. Oncogene. 2017;36:5829–39. 10.1038/onc.2017.188. 28604752 10.1038/onc.2017.188

[210] Cheng H, Qiu Y, Xu Y, Chen L, Ma K, Tao M, et al. Extracellular acidosis restricts one-carbon metabolism and preserves T cell stemness. Nat Metab. 2023;5:314–30. 10.1038/s42255-022-00730-6. 36717749 10.1038/s42255-022-00730-6 PMC9970874

[211] Mendler AN, Hu B, Prinz PU, Kreutz M, Gottfried E, Noessner E. Tumor lactic acidosis suppresses CTL function by Inhibition of p38 and JNK/c-Jun activation. Int J Cancer. 2012;131:633–40. 10.1002/ijc.26410. 21898391 10.1002/ijc.26410

[212] Ganapathy V, Thangaraju M, Prasad PD. Nutrient transporters in cancer: relevance to Warburg hypothesis and beyond. Pharmacol Ther. 2009;121:29–40. 10.1016/j.pharmthera.2008.09.005. 18992769 10.1016/j.pharmthera.2008.09.005

[213] Blaszczak W, Williams H, Swietach P. Autoregulation of H(+)/lactate efflux prevents monocarboxylate transport (MCT) inhibitors from reducing glycolytic lactic acid production. Br J Cancer. 2022;127:1365–77. 10.1038/s41416-022-01910-7. 35840734 10.1038/s41416-022-01910-7 PMC9519749

[214] Feng Q, Liu Z, Yu X, Huang T, Chen J, Wang J, et al. Lactate increases stemness of CD8 + T cells to augment anti-tumor immunity. Nat Commun. 2022;13:4981. 10.1038/s41467-022-32521-8. 36068198 10.1038/s41467-022-32521-8 PMC9448806

[215] Pérez-Tomás R, Pérez-Guillén I. Lactate in the tumor microenvironment: an essential molecule in cancer progression and treatment. Cancers (Basel). 2020;12. 10.3390/cancers12113244. 10.3390/cancers12113244 PMC7693872 33153193

[216] Sonveaux P, Copetti T, De Saedeleer CJ, Végran F, Verrax J, Kennedy KM, et al. Targeting the lactate transporter MCT1 in endothelial cells inhibits lactate-induced HIF-1 activation and tumor angiogenesis. PLoS ONE. 2012;7:e33418. 10.1371/journal.pone.0033418. 22428047 10.1371/journal.pone.0033418 PMC3302812

[217] Martínez-Zaguilán R, Seftor EA, Seftor RE, Chu YW, Gillies RJ, Hendrix MJ. Acidic pH enhances the invasive behavior of human melanoma cells. Clin Exp Metastasis. 1996;14:176–86. 10.1007/bf00121214. 8605731 10.1007/BF00121214

[218] Kato Y, Ozawa S, Miyamoto C, Maehata Y, Suzuki A, Maeda T, et al. Acidic extracellular microenvironment and cancer. Cancer Cell Int. 2013;13:89. 10.1186/1475-2867-13-89. 24004445 10.1186/1475-2867-13-89 PMC3849184

[219] Calcinotto A, Filipazzi P, Grioni M, Iero M, De Milito A, Ricupito A, et al. Modulation of microenvironment acidity reverses anergy in human and murine tumor-infiltrating T lymphocytes. Cancer Res. 2012;72:2746–56. 10.1158/0008-5472.Can-11-1272. 22593198 10.1158/0008-5472.CAN-11-1272

[220] Gottlob K, Majewski N, Kennedy S, Kandel E, Robey RB, Hay N. Inhibition of early apoptotic events by Akt/PKB is dependent on the first committed step of Glycolysis and mitochondrial hexokinase. Genes Dev. 2001;15:1406–18. 10.1101/gad.889901. 11390360 10.1101/gad.889901 PMC312709

[221] Wilson MC, Meredith D, Fox JE, Manoharan C, Davies AJ, Halestrap AP. Basigin (CD147) is the target for organomercurial Inhibition of monocarboxylate transporter isoforms 1 and 4: the ancillary protein for the insensitive MCT2 is EMBIGIN (gp70). J Biol Chem. 2005;280:27213–21. 10.1074/jbc.M411950200. 15917240 10.1074/jbc.M411950200

[222] Pan W, Tao C, Wu Y, Jiang F. A medicinal chemistry perspective on lactate dehydrogenase: current status and future directions. J Med Chem. 2025;68:16869–900. 10.1021/acs.jmedchem.5c00182. 40764921 10.1021/acs.jmedchem.5c00182

[223] Guan X, Bryniarski MA, Morris ME. In vitro and in vivo efficacy of the monocarboxylate transporter 1 inhibitor AR-C155858 in the murine 4T1 breast cancer tumor model. Aaps J. 2018;21:3. 10.1208/s12248-018-0261-2. 30397860 10.1208/s12248-018-0261-2 PMC6536115

[224] Dunbar EM, Coats BS, Shroads AL, Langaee T, Lew A, Forder JR, et al. Phase 1 trial of Dichloroacetate (DCA) in adults with recurrent malignant brain tumors. Invest New Drugs. 2014;32:452–64. 10.1007/s10637-013-0047-4. 24297161 10.1007/s10637-013-0047-4 PMC4455946

[225] Carapella CM, Paggi MG, Cattani F, Ciottoli GB, Floridi A, Iandolo B, et al. The potential role of Lonidamine (LND) in the treatment of malignant glioma. Phase II study. J Neurooncol. 1989;7:103–8. 10.1007/bf00149384. 2666593 10.1007/BF00149384

[226] Sun J, Hemler ME. Regulation of MMP-1 and MMP-2 production through CD147/extracellular matrix metalloproteinase inducer interactions. Cancer Res. 2001;61:2276–81. 11280798

[227] Bougatef F, Quemener C, Kellouche S, Naïmi B, Podgorniak MP, Millot G, et al. EMMPRIN promotes angiogenesis through hypoxia-inducible factor-2alpha-mediated regulation of soluble VEGF isoforms and their receptor VEGFR-2. Blood. 2009;114:5547–56. 10.1182/blood-2009-04-217380. 19837976 10.1182/blood-2009-04-217380

[228] Du M, Yu T, Zhan Q, Li H, Zou Y, Geng M, et al. Development of a novel lactate dehydrogenase A inhibitor with potent antitumor activity and immune activation. Cancer Sci. 2022;113:2974–85. 10.1111/cas.15468. 35722994 10.1111/cas.15468 PMC9459323

[229] Miholjcic TBS, Halse H, Bonvalet M, Bigorgne A, Rouanne M, Dercle L, et al. Rationale for LDH-targeted cancer immunotherapy. Eur J Cancer. 2023;181:166–78. 10.1016/j.ejca.2022.11.032. 36657325 10.1016/j.ejca.2022.11.032

[230] Knispel S, Gassenmaier M, Menzies AM, Loquai C, Johnson DB, Franklin C, et al. Outcome of melanoma patients with elevated LDH treated with first-line targeted therapy or PD-1-based immune checkpoint Inhibition. Eur J Cancer. 2021;148:61–75. 10.1016/j.ejca.2021.01.034. 33735811 10.1016/j.ejca.2021.01.034

[231] Verma S, Budhu S, Serganova I, Dong L, Mangarin LM, Khan JF, et al. Pharmacologic LDH Inhibition redirects intratumoral glucose uptake and improves antitumor immunity in solid tumor models. J Clin Invest. 2024;134. 10.1172/jci177606. 10.1172/JCI177606 PMC11364391 39225102

[232] Yan L, Wang Y, Hu H, Yang D, Wang W, Luo Z, et al. Physical exercise mediates cortical synaptic protein lactylation to improve stress resilience. Cell Metab. 2024;36:2104–e21172104. 10.1016/j.cmet.2024.07.018. 39163863 10.1016/j.cmet.2024.07.018

[233] Brooks GA. The lactate shuttle during exercise and recovery. Med Sci Sports Exerc. 1986;18:360–8. 10.1249/00005768-198606000-00019. 3523107 10.1249/00005768-198606000-00019

[234] Brooks GA. Lactate as a fulcrum of metabolism. Redox Biol. 2020;35:101454. 10.1016/j.redox.2020.101454. 32113910 10.1016/j.redox.2020.101454 PMC7284908

[235] Takahashi K, Kitaoka Y, Yamamoto K, Matsunaga Y, Hatta H. Oral lactate administration additively enhances endurance Training-Induced increase in cytochrome C oxidase activity in mouse soleus muscle. Nutrients. 2020;12. 10.3390/nu12030770. 10.3390/nu12030770 PMC7146285 32183387

[236] Netzahualcoyotzi C, Pellerin L. Neuronal and astroglial monocarboxylate transporters play key but distinct roles in hippocampus-dependent learning and memory formation. Prog Neurobiol. 2020;194:101888. 10.1016/j.pneurobio.2020.101888. 32693190 10.1016/j.pneurobio.2020.101888

[237] Gao V, Suzuki A, Magistretti PJ, Lengacher S, Pollonini G, Steinman MQ, et al. Astrocytic β2-adrenergic receptors mediate hippocampal long-term memory consolidation. Proc Natl Acad Sci U S A. 2016;113:8526–31. 10.1073/pnas.1605063113. 27402767 10.1073/pnas.1605063113 PMC4968707

[238] Zhou Z, Okamoto K, Onodera J, Hiragi T, Andoh M, Ikawa M, et al. Astrocytic cAMP modulates memory via synaptic plasticity. Proc Natl Acad Sci U S A. 2021;118. 10.1073/pnas.2016584118. 10.1073/pnas.2016584118 PMC7826339 33452135

[239] Jacobs RA, Meinild AK, Nordsborg NB, Lundby C. Lactate oxidation in human skeletal muscle mitochondria. Am J Physiol Endocrinol Metab. 2013;304:E686–694. 10.1152/ajpendo.00476.2012. 23384769 10.1152/ajpendo.00476.2012

[240] Morris D. Effects of oral lactate consumption on metabolism and exercise performance. Curr Sports Med Rep. 2012;11:185–8. 10.1249/JSR.0b013e31825da992. 22777328 10.1249/JSR.0b013e31825da992

[241] Bordoli C, Varley I, Sharpe GR, Johnson MA, Hennis PJ. Effects of oral lactate supplementation on Acid-Base balance and prolonged High-Intensity interval cycling performance. J Funct Morphol Kinesiol. 2024;9. 10.3390/jfmk9030139. 10.3390/jfmk9030139 PMC11348031 39189224

[242] Ewell TR, Bomar MC, Brown DM, Brown RL, Kwarteng BS, Thomson DP, et al. The influence of acute oral lactate supplementation on responses to cycle ergometer exercise: A Randomized, crossover pilot clinical trial. Nutrients. 2024;16. 10.3390/nu16162624. 10.3390/nu16162624 PMC11357576 39203761

[243] Luo F, Shao T, Liu X, Yang Q, Gai Y, Ma G, et al. Dose and age dependent effects of lactate supplementation in shaping gut microbiota. J Funct Foods. 2024;122:106467. 10.1016/j.jff.2024.106467.

[244] Scaldaferri F, Gerardi V, Lopetuso LR, Del Zompo F, Mangiola F, Boškoski I et al. Gut microbial flora, prebiotics, and probiotics in IBD: their current usage and utility. *Biomed Res Int* 2013; 2013: 435268. 10.1155/2013/435268 10.1155/2013/435268 PMC3749555 23991417

[245] Li D, Liu Z, Fan X, Zhao T, Wen D, Huang X, et al. Lactic acid Bacteria-Gut-Microbiota-Mediated intervention towards inflammatory bowel disease. Microorganisms. 2024;12. 10.3390/microorganisms12091864. 10.3390/microorganisms12091864 PMC11433943 39338538

[246] Kostic AD, Xavier RJ, Gevers D. The Microbiome in inflammatory bowel disease: current status and the future ahead. Gastroenterology. 2014;146:1489–99. 10.1053/j.gastro.2014.02.009. 24560869 10.1053/j.gastro.2014.02.009 PMC4034132

[247] Olekhnovich EI, Batotsyrenova EG, Yunes RA, Kashuro VA, Poluektova EU, Veselovsky VA, et al. The effects of Levilactobacillus brevis on the physiological parameters and gut microbiota composition of rats subjected to desynchronosis. Microb Cell Fact. 2021;20:226. 10.1186/s12934-021-01716-x. 34930242 10.1186/s12934-021-01716-x PMC8686522

[248] Deng M, Wu X, Duan X, Xu J, Yang X, Sheng X, et al. Lactobacillus paracasei L9 improves colitis by expanding butyrate-producing bacteria that inhibit the IL-6/STAT3 signaling pathway. Food Funct. 2021;12:10700–13. 10.1039/d1fo02077c. 34605504 10.1039/d1fo02077c

[249] Saadatzadeh A, Atyabi F, Fazeli MR, Dinarvand R, Jamalifar H, Abdolghaffari AH, et al. Biochemical and pathological evidences on the benefit of a new biodegradable nanoparticles of probiotic extract in murine colitis. Fundam Clin Pharmacol. 2012;26:589–98. 10.1111/j.1472-8206.2011.00966.x. 21771055 10.1111/j.1472-8206.2011.00966.x

[250] Darfeuille-Michaud A, Boudeau J, Bulois P, Neut C, Glasser AL, Barnich N, et al. High prevalence of adherent-invasive Escherichia coli associated with ileal mucosa in crohn’s disease. Gastroenterology. 2004;127:412–21. 10.1053/j.gastro.2004.04.061. 15300573 10.1053/j.gastro.2004.04.061

[251] Xiong XY, Pan XR, Luo XX, Wang YF, Zhang XX, Yang SH, et al. Astrocyte-derived lactate aggravates brain injury of ischemic stroke in mice by promoting the formation of protein lactylation. Theranostics. 2024;14:4297–317. 10.7150/thno.96375. 39113798 10.7150/thno.96375 PMC11303085

[252] Lagarde D, Jeanson Y, Barreau C, Moro C, Peyriga L, Cahoreau E, et al. Lactate fluxes mediated by the monocarboxylate transporter-1 are key determinants of the metabolic activity of beige adipocytes. J Biol Chem. 2021;296:100137. 10.1074/jbc.RA120.016303. 33268383 10.1074/jbc.RA120.016303 PMC7949083

[253] Sada N, Lee S, Katsu T, Otsuki T, Inoue T. Epilepsy treatment. Targeting LDH enzymes with a stiripentol analog to treat epilepsy. Science. 2015;347:1362–7. 10.1126/science.aaa1299. 25792327 10.1126/science.aaa1299

[254] Cai H, Chen X, Liu Y, Chen Y, Zhong G, Chen X, et al. Lactate activates trained immunity by fueling the Tricarboxylic acid cycle and regulating histone lactylation. Nat Commun. 2025;16:3230. 10.1038/s41467-025-58563-2. 40185732 10.1038/s41467-025-58563-2 PMC11971257

[255] Pan RY, He L, Zhang J, Liu X, Liao Y, Gao J, et al. Positive feedback regulation of microglial glucose metabolism by histone H4 lysine 12 lactylation in alzheimer’s disease. Cell Metab. 2022;34:634–e648636. 10.1016/j.cmet.2022.02.013. 35303422 10.1016/j.cmet.2022.02.013

[256] Guo Y, Chu L, Shui W, Hu H, Hao L, Wang D, et al. HISTONE LACTYLATION IN IMMUNE CELLS AND ITS PREDICTIVE ROLE IN SEPSIS PROGRESSION: A PROSPECTIVE OBSERVATIONAL STUDY. Shock. 2025. 10.1097/shk.0000000000002659. 40663442 10.1097/SHK.0000000000002659 PMC13132054

[257] Le A, Cooper CR, Gouw AM, Dinavahi R, Maitra A, Deck LM, et al. Inhibition of lactate dehydrogenase A induces oxidative stress and inhibits tumor progression. Proc Natl Acad Sci U S A. 2010;107:2037–42. 10.1073/pnas.0914433107. 20133848 10.1073/pnas.0914433107 PMC2836706

[258] Rellinger EJ, Craig BT, Alvarez AL, Dusek HL, Kim KW, Qiao J, et al. FX11 inhibits aerobic Glycolysis and growth of neuroblastoma cells. Surgery. 2017;161:747–52. 10.1016/j.surg.2016.09.009. 27919448 10.1016/j.surg.2016.09.009 PMC5369647

[259] Manerba M, Vettraino M, Fiume L, Di Stefano G, Sartini A, Giacomini E, et al. Galloflavin (CAS 568-80-9): a novel inhibitor of lactate dehydrogenase. ChemMedChem. 2012;7:311–7. 10.1002/cmdc.201100471. 22052811 10.1002/cmdc.201100471

[260] Farabegoli F, Vettraino M, Manerba M, Fiume L, Roberti M, Di Stefano G. Galloflavin, a new lactate dehydrogenase inhibitor, induces the death of human breast cancer cells with different glycolytic attitude by affecting distinct signaling pathways. Eur J Pharm Sci. 2012;47:729–38. 10.1016/j.ejps.2012.08.012. 22954722 10.1016/j.ejps.2012.08.012

[261] Vettraino M, Manerba M, Govoni M, Di Stefano G. Galloflavin suppresses lactate dehydrogenase activity and causes MYC downregulation in Burkitt lymphoma cells through NAD/NADH-dependent Inhibition of sirtuin-1. Anticancer Drugs. 2013;24:862–70. 10.1097/CAD.0b013e328363ae50. 23797802 10.1097/CAD.0b013e328363ae50

[262] Granchi C, Roy S, Giacomelli C, Macchia M, Tuccinardi T, Martinelli A, et al. Discovery of N-hydroxyindole-based inhibitors of human lactate dehydrogenase isoform A (LDH-A) as starvation agents against cancer cells. J Med Chem. 2011;54:1599–612. 10.1021/jm101007q. 21332213 10.1021/jm101007q

[263] Oshima N, Ishida R, Kishimoto S, Beebe K, Brender JR, Yamamoto K, et al. Dynamic imaging of LDH inhibition in tumors reveals rapid in vivo metabolic rewiring and vulnerability to combination therapy. Cell Rep. 2020;30:1798–e18101794. 10.1016/j.celrep.2020.01.039. 32049011 10.1016/j.celrep.2020.01.039 PMC7039685

[264] Kim EY, Chung TW, Han CW, Park SY, Park KH, Jang SB, et al. A novel lactate dehydrogenase Inhibitor, 1-(Phenylseleno)-4-(Trifluoromethyl) Benzene, suppresses tumor growth through apoptotic cell death. Sci Rep. 2019;9:3969. 10.1038/s41598-019-40617-3. 30850682 10.1038/s41598-019-40617-3 PMC6408513

[265] Di Magno L, Coluccia A, Bufano M, Ripa S, La Regina G, Nalli M, et al. Discovery of novel human lactate dehydrogenase inhibitors: Structure-based virtual screening studies and biological assessment. Eur J Med Chem. 2022;240:114605. 10.1016/j.ejmech.2022.114605. 35868126 10.1016/j.ejmech.2022.114605

